# Maternal dietary diversity and its correlates in a semi-urban municipality of Nepal: A cross-sectional study

**DOI:** 10.1371/journal.pgph.0005711

**Published:** 2026-08-25

**Authors:** Reshika Rimal, Ayusha Rimal, Pranil Man Singh Pradhan

**Affiliations:** 1 Institute of Medicine, Tribhuvan University, Kathmandu, Nepal; 2 Gandaki Medical College Teaching Hospital and Research Center, Pokhara, Nepal; African Population and Health Research Center, KENYA

## Abstract

Maternal dietary diversity is vital for the health of both mothers and children during lactation, yet it is often compromised in low- and middle-income countries. This cross-sectional study among 251 lactating mothers in Tarakeswor Municipality, Nepal, assessed dietary diversity using a 24-hour dietary recall and the Minimum Dietary Diversity for Women (MDD-W) indicator. Overall, 68.1% of mothers achieved the minimum dietary diversity (≥5 of 10 food groups), with a mean score of 5.03 ± 1.25. In multivariable analysis, higher odds of meeting MDD were observed among mothers with secondary or higher education (aOR = 7.5; 95% CI: 3.8–15.0), employment (aOR = 2.9; 95% CI: 1.4–5.8), joint or extended family structure (aOR = 3.7; 95% CI: 1.9–7.0), the highest wealth quintile (aOR = 4.2; 95% CI: 1.9–9.1), food-secure households (aOR = 4.5; 95% CI: 2.3–7.9), adequate nutrition knowledge (aOR = 5.2; 95% CI: 2.7–9.8), ≥ 4 antenatal care visits (aOR = 1.9; 95% CI: 1.0–3.4), and higher empowerment (aOR = 3.9; 95% CI: 1.9–7.8). These findings highlight substantial socioeconomic disparities in maternal dietary diversity and underscore the need for integrated, equity-focused nutrition interventions in rapidly urbanizing settings in low- and middle-income countries. These findings can inform targeted maternal nutrition programs in semi-urban municipalities of Nepal by prioritizing nutrition education, improving household food security, and integrating dietary diversity counseling into antenatal and postnatal care services.

## Introduction

Dietary diversity is defined as the number of different food groups consumed over a given reference period, which is a cornerstone of adequate nutrition and a critical determinant of women’s health during the reproductive period [1–3]. It serves as a reliable proxy for overall diet quality and micronutrient adequacy, both of which are essential for sustaining maternal and child health [4,5]. The Minimum Dietary Diversity for Women (MDD-W) indicator is defined as the consumption of at least five out of ten specified food groups in the previous 24 hours [6]. It is a validated measure used globally to assess the adequacy of women’s diets [7]. Ensuring that lactating mothers achieve this threshold is vital, as it directly influences the nutritional composition of breast milk, maternal recovery, and the growth and immune development of infants [8,9].

However, in many low- and middle-income countries (LMICs), including Nepal, achieving adequate dietary diversity among reproductive women remains a persistent challenge [10,11]. Maternal diets are often dominated by starchy staples such as rice, wheat, or maize, with limited inclusion of animal-source foods, legumes, fruits, and vegetables [12]. These patterns result from a complex interplay of economic constraints, limited food availability, and sociocultural norms that shape women’s access to and control over food resources [13,14]. According to national survey data, more than half of Nepali women of reproductive age do not meet the MDD-W threshold, highlighting widespread dietary inadequacy and vulnerability to micronutrient deficiencies [15].

Recent global evidence suggests that targeted nutrition interventions can improve women’s dietary diversity in low- and middle-income countries. Studies have shown that behavior change communication, nutrition education, and women’s empowerment programs can significantly increase the consumption of diverse and nutrient-rich foods among mothers and reproductive-age women [16]. In addition, nutrition-sensitive interventions such as conditional cash transfers, food voucher programs, and integrated maternal health services have demonstrated positive effects on dietary diversity and household food security in several LMIC settings [17,18]. These findings highlight the importance of combining nutrition education with broader social and economic support to improve maternal dietary practices [16–18].

One of the primary barriers to dietary diversity is household food insecurity, which refers to limited or uncertain access to sufficient and nutritious food [19]. In food-insecure households, economic hardship often forces families to rely on inexpensive staple foods, leading to monotonous diets that lack micronutrient density [20]. Beyond economic limitations**,** cultural beliefs and postpartum dietary taboos also influence maternal food consumption [21]. For instance, a study in Bhaktapur, Nepal, documented that lactating mothers commonly avoided nutrient-rich foods, such as fish, green leafy vegetables, and eggs, due to the belief that these foods might adversely affect the infant [22]. These practices, though deeply rooted in local traditions, can exacerbate nutritional risks during a physiologically demanding period [23].

Environmental and structural challenges further restrict access to a diverse diet [24]. Seasonal food shortages, particularly during winter months, and increasing food prices limit the availability and affordability of perishable foods such as fruits, vegetables, and animal products [25]. In Nepal, semi-urban municipalities are administratively classified as urban municipalities but continue to retain characteristics of both rural and urban environments, including mixed livelihoods, evolving food systems, and varying access to services [26]. In Nepal, semi-urban municipalities are typically characterized by mixed livelihoods, where households may rely on both subsistence agriculture and wage-based employment while experiencing increasing exposure to urban markets, infrastructure, and services [27]. Unlike rural areas, where food systems are largely agriculture-based, or urban areas where food access is predominantly market-driven, semi-urban populations often experience a hybrid food environment that combines elements of both systems [28]. Tarakeswor Municipality exemplifies this transition, with rapid population growth, expanding market access, and continued reliance on traditional household food practices. These settings frequently undergo rapid demographic and socioeconomic changes, including migration, expansion of food markets, and shifts in dietary patterns. As a result, access to diverse and nutritious foods may be shaped by both traditional practices and emerging urban influences [29,30].

Semi-urban and rural families are particularly affected, as they face both the economic pressures of urbanization and the resource limitations typical of rural settings [31]**.** This dynamic reflects a broader pattern across LMICs, where populations in transitional zones experience a “double burden” of undernutrition and emerging diet-related chronic diseases despite improvements in infrastructure and market access [32,33].

Despite government and development efforts to strengthen maternal and child nutrition services in Nepal**,** undernutrition among women remains a major public health concern [34]**.**The 2022 Nepal Demographic and Health Survey reported that 17% of women aged 15–49 years are underweight, with higher prevalence in rural and semi-urban areas [35]. While extensive research has examined maternal dietary patterns in rural and urban areas, semi-urban settings remain underexplored**.** These transitional environments are unique because they combine rural livelihoods and urban influences, creating a complex nutritional landscape shaped by rapid socioeconomic change, evolving food systems, and persistent inequality [36].

Understanding dietary diversity among lactating mothers in these contexts is vital for designing equitable nutrition programs. Semi-urban municipalities are home to a growing segment of Nepal’s population and represent a critical frontier for achieving Sustainable Development Goal 2, ending hunger and improving nutrition [37,38]. Yet, most national policies and interventions continue to generalize urban and rural patterns, overlooking the nuanced realities of semi-urban families.

Therefore, this study aimed to assess the prevalence of minimum dietary diversity and identify its associated sociodemographic, economic, and household-level determinants**,** including food security, among lactating mothers in a semi-urban municipality of Nepal. By addressing this evidence gap, the study seeks to inform locally tailored, equity-driven interventions to promote maternal dietary diversity and improve health outcomes in rapidly urbanizing LMIC settings. To our knowledge, this is among the few studies focusing specifically on lactating women in Nepal’s transitional urban contexts, where rural and urban determinants of nutrition intersect.

## Methods

### Study design and setting

This was a community-based cross-sectional study conducted between October 2020 and March 2021 in Tarakeswor Municipality, a semi-urban area located in Kathmandu District, Nepal. Data collection and participant recruitment took place from 09/10/2020 to 24/03/2021.

**Fig 1:**
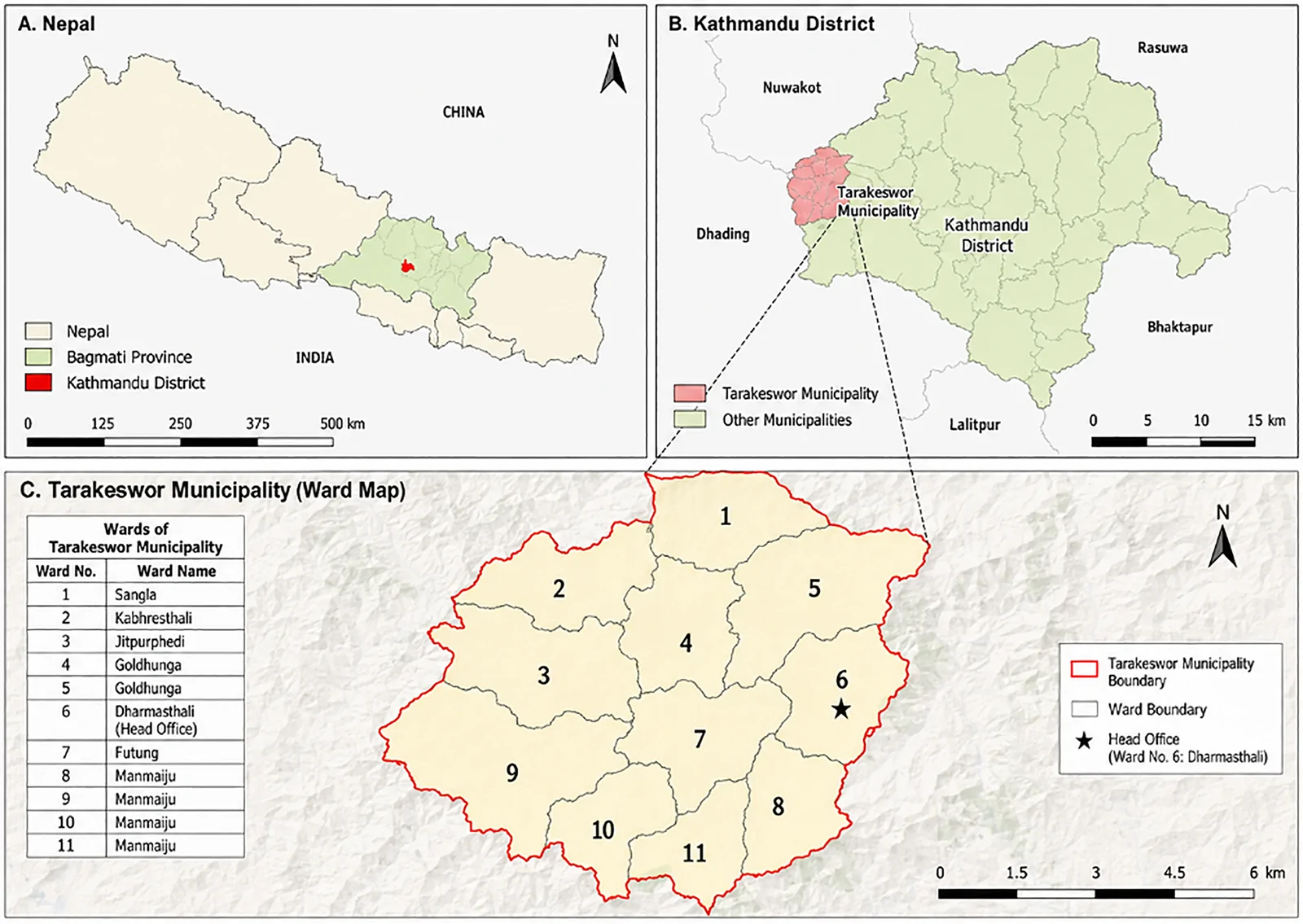
Location of Tarakeshwor Municipality in Kathmandu District, Bagmati Province, Nepal. **(A)** Nepal showing the location of Kathmandu District; **(B)** Kathmandu District showing Tarakeshwor Municipality; and **(C)** Tarakeshwor Municipality with its 11 administrative wards. The map was prepared using ArcGIS Pro (Esri Inc., Redlands, CA, USA). Administrative boundary data were obtained from **LocalBoundaries, Open Knowledge Nepal** (https://localboundries.oknp.org/), which is licensed under the **Creative Commons Attribution 4.0 International (CC BY 4.0)** license (https://creativecommons.org/licenses/by/4.0/). The figure was created by the authors.

The municipality consists of 11 administrative wards and reflects both urban and rural characteristics, with a mix of socio-economic and cultural backgrounds [39]. According to municipal records, the estimated population of Tarakeswor was approximately 80,000 in 2020 [40]. The geographical location of the study area is presented in Fig 1.

### Study population and eligibility criteria

The study population included lactating mothers with children under 24 months of age who were residing in the study area for at least six months. Eligible participants were women who were currently breastfeeding at the time of data collection. Mothers were excluded if they had a serious illness or were unable to respond to the interviewer-administered questionnaire due to physical or cognitive limitations.

A total of 700 eligible lactating mothers were identified across all 11 administrative wards of Tarakeswor Municipality through field enumeration conducted with the support of Female Community Health Volunteers (FCHVs) and local health facilities. A total of 700 eligible lactating mothers were identified across all 11 administrative wards of the Tarakeswor municipality, from which 251 mothers were selected using proportionate stratified random sampling [39]. Each ward served as a stratum, and the number of mothers selected was proportionate to the total number of lactating mothers identified in that ward. The ward-wise distribution and final sample allocation are detailed in the Sampling Method section.

### Sample size and sampling method

The sample size was determined using the single population proportion formula [41]:


$$\begin{array}{c} 𝐧=\;\frac{{𝐙}^{\text{2}}\cdot p\cdot (1-p)}{{𝐝}^{\text{2}}} \end{array}$$

*Z* = 1.96 (standard normal value for 95% CI)

*p* = estimated prevalence of minimum dietary diversity (45%) from a similar study done in the periurban area of Nepal [22]

*d* = margin of error (5%)


$$\begin{array}{c} \begin{array}{c} \text{n}=\frac{Z\text{2}\cdot p\cdot (\text{1}-p)}{{\text{d}}^{\text{2}}}\\ =\frac{\left(\text{1}.\text{96})\text{2}\cdot \text{0}.\text{45}\cdot (\text{1}-\text{0}.\text{45}\right)}{(\text{0}.\text{05}{)}^{\text{2}}}\\ =\text{ 380}.\text{48} \end{array} \end{array}$$

The finite population correction was applied using the estimated source population available from municipal records during the study planning phase (N = 2500). Subsequently, a field enumeration conducted with the support of Female Community Health Volunteers (FCHVs) and local health facilities identified 700 eligible lactating mothers across the municipality, which constituted the final sampling frame for participant selection.

Since the total number of lactating mothers in the study area was below 10,000, the sample size was adjusted using the finite population correction formula:


$$\begin{array}{c} \begin{array}{c} \text{n}\_\text{adjusted}=\frac{\text{n}}{\text{1}+(\frac{n-\text{1}}{N})}\\ =\frac{\text{380}.\text{84}}{\text{1}+(\frac{\text{3}\text{7}\text{9}.\text{4}\text{8}}{\text{2}\text{5}\text{0}\text{0}})}=\text{ 239 } \end{array} \end{array}$$

Finally, accounting for a 10% non-response rate:


$$\begin{array}{c} \text{Final N }=\text{239 }+\;(\text{239 }*\;\text{0}.\text{1})\;=\text{ 263} \end{array}$$

A comprehensive sampling frame was created in coordination with Female Community Health Volunteers (FCHVs) and local health facilities, drawing from immunization records, growth monitoring charts, and household registers maintained by FCHVs to identify eligible participants. FCHVs are trained female community members who serve as primary health liaisons between the government health system and local populations, playing a critical role in maternal and child health service delivery in Nepal [42]. The number of lactating mothers was determined in each ward, and the sample was proportionately allocated. Within each ward, participants were selected using simple random sampling to ensure equal probability of selection and minimize selection bias. The ward-wise distribution and final sample allocation are shown in **Table 1**.

**Table 1:** Ward-wise distribution of total lactating mothers and sample size selected (n = 263).

Ward Number	Total Lactating Mothers	Sample Size Selected
Ward 1	55	21
Ward 2	60	23
Ward 3	40	15
Ward 4	75	28
Ward 5	30	11
Ward 6	120	45
Ward 7	90	34
Ward 8	45	17
Ward 9	65	24
Ward 10	50	19
Ward 11	70	26
**Total**	**700**	**263**

Although 263 mothers were selected, only 251 completed the interviews, resulting in a response rate of 95.4%. Twelve mothers either declined to participate or were unavailable despite repeated follow-up visits. These individuals were excluded from the final analysis.

## Study variables

### Outcome variable

#### Dietary diversity.

Dietary diversity was assessed using the Minimum Dietary Diversity for Women (MDD-W) indicator developed by the FAO [6]. A 24-hour dietary recall method was used to record all foods and beverages consumed by participants. Dietary diversity was assessed using the Minimum Dietary Diversity for Women (MDD-W) indicator, which classifies foods into ten groups: (1) grains, white roots and tubers, and plantains; (2) pulses (beans, peas, and lentils); (3) nuts and seeds; (4) dairy products; (5) meat, poultry, and fish; (6) eggs; (7) dark green leafy vegetables; (8) other vitamin A-rich fruits and vegetables; (9) other vegetables; and (10) other fruits. Women who consumed foods from five or more of these food groups were considered to have achieved minimum dietary diversity, while those who consumed fewer than five groups were categorized as not achieving it [43].

#### Independent variables: Sociodemographic variables.

These included maternal age (in completed years), educational status (no formal education, primary, secondary, or higher), and employment status (employed or unemployed). Household type was classified as either joint or nuclear, and monthly household income was categorized into tertiles (low, middle, and high). These classifications were based on standard frameworks used in the Nepal Demographic and Health Survey [35].

#### Wealth quintile.

Household wealth status was assessed using a composite wealth index, constructed through Principal Component Analysis (PCA). Variables included household assets (e.g., radio, mobile phone, refrigerator), housing structure (floor and roof materials), type of cooking fuel, and access to electricity, drinking water, and sanitation. Based on PCA scores, households were categorized into five quintiles: poorest, poorer, middle, richer, and richest. This method followed the Nepal Demographic and Health Survey [35].

#### Nutrition knowledge.

Maternal knowledge of nutrition during lactation was assessed using ten structured questions, adapted from the Food and Agriculture Organization (FAO) Knowledge, Attitudes, and Practices (KAP) framework [44]. Questions covered topics such as dietary diversity, sources of essential nutrients, iron-folic acid supplementation, and the consequences of undernutrition. Each correct response received one point. Participants scoring ≥5 out of 10 were categorized as having adequate knowledge, while those scoring <5 were categorized as having inadequate knowledge. This classification was based on standard frameworks used in the Nepal Demographic and Health Survey [35].

### Household food insecurity access scale (HFIAS)

(HFIAS), a standardized tool developed by the Food and Nutrition Technical Assistance (FANTA) Project [45]. It includes nine structured questions that evaluate experiences related to uncertainty about food supply, insufficient food quality, and inadequate food intake over the previous 30 days. Each question was followed by a frequency-of-occurrence item scored as 0 (never), 1 (rarely), 2 (sometimes), or 3 (often), resulting in a total score ranging from 0 to 27, with higher scores indicating more severe food insecurity. Based on the total score, households were initially categorized into four groups: food secure, mildly food insecure, moderately food insecure, and severely food insecure. For analytical purposes, this variable was further dichotomized into two categories: food secure (including only food secure) and food insecure (combining mildly, moderately, and severely food insecure households). This method followed the Nepal Demographic and Health Survey [35].

### Women’s empowerment

Women’s empowerment was assessed using key decision-making indicators adapted from the Nepal Demographic and Health Survey (NDHS) [35]. Respondents were asked whether they participated in decision-making regarding their own health care, major household purchases, and visits to family or relatives. Responses were scored and summed, and participants were categorized into two groups: empowered (participated in one or more decisions) and not empowered (did not participate in any of the decisions). This dichotomous categorization was adopted to facilitate statistical analysis and because of the limited variability across individual decision-making domains. This variable was used to assess the role of autonomy and agency in influencing maternal dietary diversity.

#### Variable coding for regression analysis.

For the regression analyses, selected variables were recategorized to ensure adequate cell sizes within categories, enhance the stability of model estimates, and improve the interpretability of the findings. Age was dichotomized as ≤24 years and >24 years. Educational attainment was categorized as secondary or lower and SLC or above. Wealth quintiles were collapsed into three categories (lowest, middle, and highest). Household food security and women’s empowerment were analyzed as binary variables as described above. These categorizations were applied consistently throughout the logistic regression analyses.

### Study procedure

Data for this study were collected using a semi-structured interviewer-administered questionnaire that was developed by the researcher based on previously validated tools and guidelines. The questionnaire was initially prepared in English and translated into Nepali to ensure clarity, cultural appropriateness, and ease of understanding for the participants. During the development and translation process, attention was given to locally relevant food practices and commonly reported postpartum dietary restrictions to ensure that questions on dietary intake were culturally appropriate and understandable to participants.

The full questionnaire is available in the S1 File. It was pretested among a similar group of lactating mothers in a neighboring municipality, and minor modifications were made based on the feedback received to improve the clarity and flow of the questions. The pretesting process also helped ensure that culturally specific dietary practices or food avoidance behaviors did not affect the interpretation of dietary recall questions.

The data collectors were two trained medical students. They received detailed training on standardized interviewing techniques and ethical research practices for 2 days. Enumerator training also included role play and field simulation to ensure consistency in data collection. To minimize potential recall bias associated with the 24-hour dietary recall, trained interviewers used probing questions and locally familiar food examples to assist participants in accurately recalling all foods and beverages consumed during the previous day. Standardized interviewing procedures were followed to improve recall accuracy. The final version of the questionnaire was piloted, refined, and used with 15 lactating mothers in a neighboring ward. Necessary modifications were made to improve clarity.

## Data analysis

Data were entered into EpiData and analyzed using SPSS version 26. Descriptive statistics, including means, standard deviations, and proportions, were used to summarize participant characteristics. Bivariate logistic regression was conducted to assess the association between independent variables and dietary diversity. Variables with p-values < 0.20 in the bivariate analysis were entered into the multivariable logistic regression model. Adjusted odds ratios (aORs) with 95% confidence intervals (CIs) were reported. All variables included in the final model were tested for multicollinearity, and none were found to exceed the threshold (VIF < 2). Model fit was assessed using the Hosmer–Lemeshow goodness-of-fit test and was acceptable (p = 0.48), indicating a good fit to the observed data. Statistical significance was set at p < 0.05.

### Ethics approval and consent to participate

Ethical approval for the study was obtained from the Institutional Review Committee (IRC) of the Institute of Medicine, Tribhuvan University, Nepal (Approval No. 86(6–1) E2/077/078). Written informed consent was obtained from all participants. For participants below the age of 20, written assent was obtained from the participant, and consent was obtained from a parent or guardian, following IRC ethical protocols. Anonymity and confidentiality were ensured throughout the study. This study was conducted in accordance with the ethical principles outlined in the Declaration of Helsinki.

## Results

### Participant characteristics

Table 2 summarizes the demographic, household, and health-related characteristics of the 251 lactating mothers included in the study. Among the 251 lactating mothers included in the study, most were aged 18–25 years (69.7%), with smaller proportions aged 26–35 years (28.3%) and above 35 years (2.0%). More than half of the respondents belonged to upper-caste groups (54.6%), followed by disadvantaged Janajati (26.7%) and Dalit communities (17.1%). The majority of participants identified as Hindu (95.6%). Three-fourths of the mothers lived in joint or extended family settings (75.3%), and most households were male-headed (68.5%). Land ownership was common, with 70.9% of households owning land.

**Table 2:** Key characteristics of the study population (n = 251).

Characteristics	n (%)
**Age Group**	
18–25 years	175 (69.7)
26–35 years	71 (28.3)
>35 years	5 (2.0)
**Ethnicity**	
Dalit	43 (17.1)
Disadvantaged Janajati	67 (26.7)
Upper Caste	137 (54.6)
Religious Minorities	4 (1.6)
**Religion**	
Hindu	240 (95.6)
Non-Hindu	11 (4.4)
**Family Type**	
Nuclear family	62 (24.7)
Joint/Extended family	189 (75.3)
**Head of Household**	
Male	172 (68.5)
Female	79 (31.5)
**Land Ownership**	
Owns land	178 (70.9)
Does not own land	73 (29.1)
**Education**	
No formal education	8 (3)
Primary education	30 (12)
Secondary education	138 (55)
College and above	75 (30)
**Employment Status**	
Employed	118 (47.0)
Unemployed	133 (53.0)
**Wealth Quintile**	
Lowest	48 (19.1)
Second	44 (17.5)
Middle	49 (19.5)
Fourth	53 (21.1)
Highest	57 (22.7)
**Parity**	
Primiparous	145 (57.7)
Multiparous	106 (42.3)
**ANC Visits**	
<4 visits	193 (76.9)
≥4 visits	58 (23.1)
**Women’s Empowerment**	
Empowered	108 (43.0)
Not Empowered	143 (57.0)
**Food Security**	
Food secure	171 (68.1)
Food insecure	80 (31.9)
**Dietary Diversity**	
Minimum dietary diversity achieved	171 (68.1)
Not achieved	80 (31.9)

Regarding education, 3% of mothers had no formal schooling, 12% had completed primary education, over half (55%) had secondary education, and 30% had attained college-level or higher education. Nearly half of the women were employed (47%). Wealth distribution was relatively even across quintiles, with 22.7% in the highest and 19.1% in the lowest quintile. More than half of the mothers were primiparous (57.7%).

In terms of healthcare utilization, only 23.1% had completed four or more antenatal care (ANC) visits. Regarding empowerment, 43% of women were categorized as empowered. Household food security was reported by 68.1% of participants. A similar proportion of mothers (68.1%) achieved minimum dietary diversity, with a mean score of 5.03 (SD ± 1.25).

### Dietary diversity among lactating mothers

The primary outcome of interest was dietary diversity, assessed using the Minimum Dietary Diversity for Women (MDD-W) indicator. Among the 251 lactating mothers, 68.1% (n = 171) met the minimum dietary diversity threshold by consuming foods from at least five out of ten defined food groups in the previous 24 hours. The mean dietary diversity score was 5.03 (SD ± 1.25). However, 31.9% (n = 80) of participants did not achieve minimum dietary diversity, indicating potential nutritional inadequacies in nearly one-third of the population.

To further explore the types of foods consumed, **Table 3** presents the proportion of lactating mothers who consumed each of the ten food groups within the past 24 hours based on the MDD-W framework. All participants consumed starchy staples such as rice, maize, and potatoes, which are culturally ingrained and widely accessible. Similarly, pulses were consumed by nearly all mothers (98.8%), reflecting their affordability and routine inclusion in traditional Nepali meals.

**Table 3:** Proportion of lactating mothers who consumed each of the 10 food groups within the past 24 hours, based on the MDD-W framework (N = 251).

Food Group	Frequency (n)	Percentage (%)
Grains, white roots, and tubers	251	100.0
Pulses (beans, peas, lentils)	248	98.8
Nuts and seeds	45	17.9
Milk and milk products	195	77.7
Eggs	73	29.1
Meat, poultry, and fish	94	37.5
Dark green leafy vegetables	132	52.6
Vitamin A-rich fruits and vegetables	39	15.5
Other vegetables	201	80.1
Other fruits	148	58.9

In contrast, the intake of several nutrient-dense food groups was substantially lower. Only 29.1% of mothers consumed eggs, 17.9% consumed nuts and seeds, and just 15.5% consumed vitamin A-rich fruits and vegetables most mothers meeting the minimum dietary diversity threshold, the low consumption of these micronutrient-rich foods suggests potential micronutrient gaps among lactating mothers and indicates a possible risk of “hidden hunger.”

### Factors associated with minimum dietary diversity among lactating mothers

Table 4 presents the multivariable logistic regression results for factors associated with achieving minimum dietary diversity among lactating mothers in Tarakeswor Municipality, Nepal. In the adjusted model, several sociodemographic and maternal characteristics remained significant predictors.

**Table 4:** Multivariable logistic regression of factors associated with minimum dietary diversity among lactating mothers in Tarakeswor Municipality, Nepal (n = 251).

Variables	Not Diverse n (%)	Diverse n (%)	Crude Odds Ratio (95% CI)	Adjusted Odds Ratio (95% CI)
**Age Groups**				
≤24 years	63 (36.0)	112(64.0)	Ref	Ref
>24 years	36 (47.5)	40 (52.5)	1.60 (0.95–2.65)	1.30 (0.70–2.20)
**Ethnicity**				
Dalit	26 (57.0)	20 (43.0)	Ref	Ref
Janajati	35 (37.0)	60 (63.0)	2.10 (1.10–4.00)*	1.80 (0.90–3.50)
Brahmin/Chhetri	32 (25.0)	96 (75.0)	3.80 (1.90–7.20)**	3.10 (1.50–6.50)*
**Family Type**				
Nuclear	72 (50.0)	72 (50.0)	Ref	Ref
Joint/Extended	49 (21.0)	184 (79.0)	4.50 (2.70–7.70)**	3.70 (1.90–7.00)*
**Education Level**				
Secondary or Lower	43 (72.0)	17 (28.0)	Ref	Ref
SLC or Above	78 (30.0)	182 (70.0)	9.00 (4.60–17.50)**	7.50 (3.80–15.00) **
**Employment**				
Unemployed	97 (46.0)	114 (54.0)	Ref	Ref
Employed	24 (20.0)	96 (80.0)	3.40 (1.80–6.60) *	2.90 (1.40–5.80) *
**Wealth Status**				
Lowest	42 (56.0)	33 (44.0)	Ref	Ref
Middle	38 (39.0)	58 (61.0)	2.10 (1.00–4.20)	1.80 (0.90–3.60)
Highest	13 (19.0)	55 (81.0)	5.00 (2.30–10.60) **	4.20 (1.90–9.10) *
**Food Security**				
Food Insecure	48 (65.0)	26 (35.0)	Ref	Ref
Food Secure	73 (26.0)	207 (74.0)	5.10 (2.80–8.90)**	4.50 (2.30–7.90)**
**Nutrition Knowledge**				
Inadequate	62 (58.0)	45 (42.0)	Ref	Ref
Adequate	59 (23.0)	198 (77.0)	6.00 (3.10–11.00)**	5.20 (2.70–9.80)**
**ANC Visits**				
<4	75 (52.2)	27 (47.8)	Ref	Ref
≥4	28 (34.2)	71 (65.8)	2.00 (1.10–3.60) *	1.90 (1.00–3.40)*
**Women Empowerment**				
Not Empowered	64 (44.8)	79 (55.2)	Ref	Ref
Empowered	16 (14.8)	92 (85.2)	4.67 (2.51–8.67) **	3.90 (1.90–7.80)**

cOR = Crude Odds Ratio; aOR = Adjusted Odds Ratio; Ref = Reference category.

*Significance levels: *p < 0.05, **p < 0.001.

Mothers residing in joint or extended families had significantly higher odds of meeting the minimum dietary diversity compared to those living in nuclear families (aOR = 3.7; 95% CI: 1.9–7.0). Similarly, those with secondary or higher education were substantially more likely to achieve dietary diversity than mothers with no formal or only primary education (aOR = 7.5; 95% CI: 3.8–15.0). Employment status was also an important determinant - employed women had almost three times higher odds of achieving minimum dietary diversity compared to unemployed women (aOR = 2.9; 95% CI: 1.4–5.8).

Household socioeconomic position showed a clear gradient effect. Women from the highest wealth quintile were over four times more likely to achieve adequate dietary diversity than those in the lowest quintile (aOR = 4.2; 95% CI: 1.9–9.1). Similarly, food security was strongly associated; mothers from food-secure households had 4.5-fold greater odds of achieving dietary diversity compared with those from food-insecure households (aOR = 4.5; 95% CI: 2.3–7.9).

Maternal nutrition knowledge emerged as another key factor. Women with adequate knowledge had over five times higher odds of meeting dietary diversity than those with inadequate knowledge (aOR = 5.2; 95% CI: 2.7–9.8).Moreover, attending four or more antenatal care (ANC) visits was associated with better dietary diversity (aOR = 1.9; 95% CI: 1.0–3.4; p = 0.048). Finally, women’s empowerment significantly increased the likelihood of achieving dietary diversity compared to those who were not empowered (aOR = 3.9; 95% CI: 1.9–7.8).

## Discussion

This study provides important evidence on maternal dietary diversity in a transitional urban setting in Nepal, where rural and urban dynamics coexist and interact. As low- and middle-income countries (LMICs) experience rapid urbanization, understanding maternal nutrition in these hybrid environments is essential for designing equitable public health interventions [46]. Tarakeswor Municipality represents such a transitional zone, exhibiting infrastructural growth while still grappling with socioeconomic disparities [40]. These unique characteristics create a hybrid food environment in which improved physical access to markets coexists with persistent economic constraints, traditional dietary practices, and unequal access to nutritious foods, all of which may influence dietary diversity among lactating mothers.Our findings thus offer insights not only for Nepal but also for other LMICs facing similar urban transformations. This cross-sectional study found that a substantial proportion of lactating mothers in Tarakeswor Municipality did not meet the minimum dietary diversity recommended by the MDD-W framework. Specifically, 68.1% of mothers achieved minimum dietary diversity (≥5 food groups), while 31.9% did not, indicating the presence of potential nutritional inadequacies in nearly one-third of the population.

Dietary diversity among lactating mothers in South Asia remains suboptimal despite its critical importance for maternal and child health [47–49]. Evidence from Nepal illustrates this concern: in remote areas, only about 28% of lactating women meet the minimum dietary diversity (MDD-W), while even in the more developed Kathmandu Valley, less than 45% reach this threshold [22,50]. These figures reflect broader trends observed across semi-urban South Asia, where maternal diet quality is shaped by complex interactions among socioeconomic, cultural, and behavioral factors [33,51,52].

In our study of lactating mothers in Tarakeswor Municipality, the mean Dietary Diversity Score (DDS) was 5.03 (±1.25), with 68% of mothers meeting the MDD-W threshold. This is higher than what has been reported in some rural Nepali settings, such as Bajhang (DDS = 4.1) [50], but lower than in neighboring countries like Bangladesh and Vietnam, where robust maternal nutrition programs exist [49,53]. Furthermore, structural barriers such as urban poverty and limited access to healthy diets have been identified as persistent challenges to achieving dietary adequacy in LMICs [31]. Despite improved access to food markets and health services, structural barriers such as poverty, low education, and gender norms remain important challenges to dietary diversity [54,55]. Although a majority of mothers in this study achieved the minimum dietary diversity threshold, the low consumption of eggs, nuts and seeds, and vitamin A-rich fruits and vegetables observed in the results suggests that micronutrient adequacy may still be limited. This pattern may suggest potential micronutrient inadequacies, whereby individuals may meet caloric requirements while consuming insufficient amounts of essential vitamins and minerals [56]. Similar concerns have been reported in several LMIC settings where dietary diversity scores may appear adequate, yet diets remain limited in nutrient-dense foods [57]. This finding highlights the importance of considering not only the number of food groups consumed but also the nutritional quality of foods included in maternal diets. Previous studies in Nepal have reported deficiencies of several key micronutrients among women of reproductive age, particularly iron, vitamin A, vitamin B12, zinc, iodine, and folate. These deficiencies have been associated with maternal anemia, impaired immune function, adverse pregnancy and lactation outcomes, and suboptimal infant growth and development. Therefore, improving dietary diversity through greater consumption of nutrient-dense foods may help reduce the burden of hidden hunger and improve maternal and child health [22].

Our findings confirm several key predictors of dietary diversity. Maternal education emerged as a powerful determinant: women with secondary or higher education were 7.5 times more likely to meet the MDD-W threshold compared to those with no or primary education. This is consistent with national survey data (NDHS, 2022) [35]and studies from across South Asia and Africa [47,58,59]. In a study conducted in Baglung Municipality, women with higher education had significantly greater dietary diversity, reinforcing the association between educational attainment and greater awareness, decision-making capacity, and access to a varied diet.[10].

Similarly, employment status was positively associated with dietary diversity. Employed mothers were nearly three times more likely to achieve the MDD-W threshold. Employment may be associated with greater income and autonomy, which could facilitate access to diverse and nutrient-dense foods. In a study conducted in Nepal, employed women had nearly five times the odds of achieving dietary diversity [10]. These findings highlight the need to promote economic opportunities for women as part of nutrition interventions.

Household wealth and food security were also critical. Mothers from the highest wealth quintile had more than four times higher odds of achieving dietary diversity than those from the lowest quintile, which is consistent with Bennett’s Law that income growth leads to more diverse diets [60]. Food-secure households were almost four times more likely to meet the MDD-W threshold, aligning with findings from Ethiopia, Bangladesh, and Nepal [47,50,61]. In Baglung Municipality, Nepal, women in the highest wealth group had more than three times the odds of achieving dietary diversity, and food insecurity was closely associated with monotonous diets [10].

Family structure was also associated with dietary diversity. Women living in joint or extended families were significantly more likely to achieve adequate dietary diversity (AOR = 3.7), possibly due to shared food resources and household support systems. While some African studies show the opposite effect due to intra-household food allocation challenges in larger families where limited resources must be divided among more members, South Asian contexts often demonstrate the benefits of communal support [10,62–64]. In these settings, extended family structures may buffer food insecurity by pooling income, sharing meals, and providing social or caregiving support to women during lactation and child-rearing.

Nutrition knowledge was one of the strongest predictors of dietary diversity in our study (AOR = 5.2). Women with higher nutrition knowledge were more likely to achieve minimum dietary diversity. This finding aligns with global evidence showing that education and counseling interventions have been associated with improved dietary outcomes [65,66].

Lastly, health service utilization, particularly antenatal care (ANC), was positively associated with dietary diversity. Women who had four or more ANC visits had nearly twice the odds of achieving dietary diversity, although the association did not reach strong statistical significance. This finding is consistent with literature from Ethiopia and South Asia; however, given the borderline statistical significance observed in this study, the association should be interpreted with caution. It shows that ANC visits may provide opportunities for nutrition education and behavior change.[49,50,61,67].

While ethnicity was significant in the bivariate analysis, it was not retained in the multivariable model. This may reflect that, in semi-urban settings like Tarakeswor, educational and economic factors outweigh ethnic disparities that are more pronounced in rural regions. A similar study in Nepal observed that socioeconomic and empowerment factors had stronger predictive value than ethnicity [10,22]. Although caste and religion were not significant predictors in the final model, sociocultural factors may still influence maternal dietary practices through cultural food preferences, dietary restrictions, household norms, and health-seeking behaviors. Future studies may further explore these relationships using qualitative or mixed-methods approaches.

These findings also highlight the need for integrated interventions that address both structural and behavioral barriers associated with maternal dietary diversity. [17]. Structural approaches may include strengthening household food security through social protection programs, livelihood support for women, and improved access to affordable nutrient-rich foods through local markets and agricultural initiatives [68]. At the same time, behavioral interventions such as nutrition education, counseling during antenatal and postnatal care, and community-based awareness programs may help address knowledge gaps and culturally rooted food taboos that influence maternal diets [69]. Evidence from LMICs suggests that combining nutrition-sensitive interventions with behavior change communication has been associated with better dietary practices and micronutrient intake than isolated strategies [16,17]. Examples of effective interventions include individualized and group-based nutrition counseling during antenatal and postnatal care, community-based behavior change communication delivered through community health workers, women’s support groups, conditional cash transfer and social protection programs, livelihood and income-generation initiatives, home gardening, and food supplementation or food voucher programs, which have been associated with improved dietary diversity and maternal nutritional outcomes in LMICs.

Overall, our findings align with regional research and highlight the importance of socioeconomic, household, and behavioral factors associated with dietary diversity.The consistency of these findings across studies suggests that multi-sectoral strategies addressing these factors can effectively enhance maternal nutrition in Nepal and similar LMIC contexts. These findings support national nutrition policies such as Nepal’s Multi-Sector Nutrition Plan and can inform targeted interventions in comparable settings undergoing rapid urban transitions [34]. Specifically, these findings suggest that policymakers should prioritize context-specific strategies that integrate maternal nutrition education, improved access to affordable nutrient-rich foods, social protection, and women’s economic empowerment to reduce hidden hunger and improve maternal and child health outcomes in rapidly urbanizing settings. Implementation of these strategies should build on existing community health systems, including Female Community Health Volunteers (FCHVs), and incorporate locally available foods, culturally appropriate nutrition education, and community engagement to ensure that interventions are contextually relevant, acceptable, and sustainable.

### Conclusion

This study showed that although 68.1% of lactating mothers in Tarakeswor Municipality met the minimum dietary diversity (MDD-W ≥ 5), nearly one-third did not, highlighting persistent nutritional vulnerabilities even in areas with access to urban services. Food insecurity was an important factor associated with inadequate dietary diversity, alongside maternal education, employment status, household wealth, nutrition knowledge, and decision-making autonomy.

As one of the few studies focusing on lactating mothers in a semi-urban, transitional municipality of Nepal, this research adds new evidence that urban proximity alone does not ensure dietary adequacy; social and economic empowerment appear to be important factors associated with maternal nutrition.

Public health efforts should therefore integrate equity-focused and multi-sectoral interventions that strengthen women’s education, economic participation, and household food security, alongside routine nutrition counseling during antenatal and postpartum care. Addressing these interconnected factors may be essential for advancing Nepal’s maternal health and nutrition goals and achieving Sustainable Development Goals 2 (Zero Hunger) and 3 (Good Health and Well-Being). Future longitudinal, intervention, and implementation studies are warranted to further examine the complex relationships between dietary diversity, micronutrient adequacy (including hidden hunger), and maternal and child health outcomes, and to evaluate the effectiveness of nutrition interventions in diverse settings across Nepal. Policymakers should prioritize integrating maternal nutrition education into routine maternal health services, expanding social protection and food security programs for vulnerable households, and promoting community-based initiatives that enhance women’s access to affordable, nutrient-rich foods in rapidly urbanizing municipalities.

### Strengths and limitations

This study is among the first to examine maternal dietary diversity specifically among lactating mothers in a semi-urban, transitional municipality of Nepal, where rural and urban dynamics intersect. Most previous research in Nepal has focused either on rural or metropolitan settings; by contrast, this study contributes new evidence from an understudied population experiencing rapid socioeconomic transition.

A key strength lies in its community-based design using proportionate stratified random sampling across all municipal wards, ensuring representativeness and minimizing selection bias. The study also utilized validated international tools the Minimum Dietary Diversity for Women (MDD-W) and Household Food Insecurity Access Scale (HFIAS)—allowing comparability with global and national datasets. Moreover, by incorporating variables such as nutrition knowledge, empowerment, and antenatal care utilization, it provides a more holistic understanding of the social determinants of maternal diet quality in Nepal.

However, several limitations should be noted. The cross-sectional design precludes causal inference. The use of a single 24-hour dietary recall may not capture habitual intake and is subject to recall bias. In addition, a single 24-hour recall may not adequately capture day-to-day variation in food consumption and does not allow for quantitative assessment of nutrient adequacy.

In addition, dietary patterns in Nepal can vary across seasons due to fluctuations in the availability and affordability of fruits, vegetables, and other nutrient-rich foods. Because the data were collected at a single time point, seasonal variation in food availability may not have been fully captured. Cultural dietary practices, including postpartum food taboos that may influence maternal food choices, were also not quantitatively assessed in this study. Additionally, although several socioeconomic and behavioral factors were included in the multivariable analysis, residual confounding may remain because potentially relevant variables such as maternal BMI, postpartum duration, breastfeeding intensity, household food expenditure, maternal morbidity, and specific cultural dietary practices were not assessed. Additionally, because the study was conducted in a single semi-urban municipality, the findings may not be generalizable to all regions of Nepal with different ecological or cultural contexts.

Measures of food security, empowerment, knowledge, and dietary intake were self-reported, which may introduce social desirability bias. Additionally, because the study was conducted in a single semi-urban municipality, the findings may not be generalizable to other ecological or cultural settings in Nepal. Seasonal variation and cultural dietary restrictions were not systematically captured. In addition, women’s empowerment was assessed using a limited set of household decision-making indicators and categorized dichotomously. Although adapted from the Nepal Demographic and Health Survey, this approach may not fully capture the multidimensional and context-specific nature of empowerment, including economic, social, and mobility-related dimensions.

## Supporting information

S1 Table Food groups used to construct the minimum dietary diversity for women of reproductive age (MDD-W). Description of the ten food groups used to calculate the Minimum Dietary Diversity for Women indicator according to the FAO MDD-W framework.
(DOCX)

S2 Table Variance inflation factor (VIF) values of independent variables included in the multivariable logistic regression model. Assessment of multicollinearity among explanatory variables included in the final regression model.
(DOCX)

S1 File Study questionnaire and informed consent form. Structured questionnaire and consent form used to collect information on sociodemographic characteristics, maternal nutrition knowledge, household food security, and 24-hour dietary recall among lactating mothers.
(DOCX)

S2 File STROBE checklist for cross-sectional studies. Completed STROBE checklist accompanying this manuscript. Adapted from the STROBE Statement (https://www.strobe-statement.org/) and distributed under the Creative Commons Attribution 4.0 International (CC BY 4.0) licence.
(DOCX)

S1 Data De-identified SPSS dataset supporting the findings of this study. The dataset contains the de-identified participant-level data underlying the results reported in this manuscript.
(SAV)
