# Supplementary material for: Maternal dietary diversity and its correlates in a semi-urban municipality of Nepal: A cross-sectional study
Source: PLOS Glob Public Health. 2026 Aug 25;6(8):e0005711. doi: 10.1371/journal.pgph.0005711 (PMC13505919; doi:10.1371/journal.pgph.0005711)
Supplement: Supplementary file 1 [file pgph.0005711.s001.docx]

S1 Table. **Food groups used to construct Minimum Dietary Diversity for Women of Reproductive Age**

| **S.N.** | **Food Group** | **Examples (Rephrased and Simplified)** |
| --- | --- | --- |
| **1** | **Grains, white roots and tubers, and plantains** | Common staple foods such as rice, wheat, maize, millet, sorghum, or foods prepared from these grains; also white potato, cassava, yam, plantain, and dishes prepared from these starchy roots or tubers. |
| **2** | **Pulses (beans, peas, lentils)** | Mature dried beans, dried peas, lentils, or foods made using these pulses. |
| **3** | **Nuts and seeds** | Edible nuts (e.g., peanuts, tree nuts) and various edible seeds consumed as snacks or ingredients. |
| **4** | **Dairy** | Milk, yogurt, cheese, and other processed or fermented dairy products. |
| **5** | **Meat, poultry, and fish** | Meat from animals such as goat, buffalo, pork, beef, or poultry; organ meats (e.g., liver, kidney); fish, shellfish, or other aquatic animals (fresh, dried, or processed). |
| **6** | **Eggs** | Eggs from chickens or other birds, consumed in any form. |
| **7** | **Dark green leafy vegetables** | Locally available leafy greens rich in vitamins, such as spinach, kale, amaranth leaves, mustard greens, or cassava leaves. |
| **8** | **Other vitamin A–rich fruits and vegetables** | Orange or yellow vegetables like pumpkin, carrot, or squash; and vitamin A–rich fruits such as ripe mango, papaya, or similar seasonal varieties. |
| **9** | **Other vegetables** | Vegetables not included above, such as tomatoes, onions, cucumbers, gourds, or other commonly consumed local varieties. |
| **10** | **Other fruits** | Fruits not listed in other categories, including seasonal, wild, cultivated fruits, or 100% natural fruit juices made from these fruits. |
