# Supplementary material for: Maternal dietary diversity and its correlates in a semi-urban municipality of Nepal: A cross-sectional study
Source: PLOS Glob Public Health. 2026 Aug 25;6(8):e0005711. doi: 10.1371/journal.pgph.0005711 (PMC13505919; doi:10.1371/journal.pgph.0005711)
Supplement: Supplementary file 2 [file pgph.0005711.s002.docx]

S4 Table. Variance Inflation Factor (VIF) values of independent variables included in the multivariable logistic regression model

| **S.N.** | **Variable** | **VIF** |
| --- | --- | --- |
| 1 | Maternal age | 1.18 |
| 2 | Ethnicity | 1.32 |
| 3 | Family type | 1.21 |
| 4 | Maternal education | 1.44 |
| 5 | Maternal employment | 1.17 |
| 6 | Household wealth status | 1.38 |
| 7 | Household food security | 1.25 |
| 8 | Nutrition knowledge | 1.31 |
| 9 | ANC visits | 1.19 |
