# Supplementary material for: Maternal dietary diversity and its correlates in a semi-urban municipality of Nepal: A cross-sectional study
Source: PLOS Glob Public Health. 2026 Aug 25;6(8):e0005711. doi: 10.1371/journal.pgph.0005711 (PMC13505919; doi:10.1371/journal.pgph.0005711)
Supplement: Supplementary file 3 [file pgph.0005711.s003.docx]

**Annex I: Informed Consent (Above 18 Years)**

**Tribhuwan University**
**Maharajgunj Medical Campus**
**Department of Community Medicine and Public Health**

**INFORMATION SHEET**

Namaste, my name is **Reshika Rimal**. I am a **Bachelor in Public Health student** at Maharajgunj Medical Campus, Institute of Medicine, Tribhuwan University, Kathmandu. As part of the requirement for the completion of my Bachelor’s degree, I am conducting a research study titled:**“Dietary diversity and its associated factors among lactating mothers in Tarakeswor Municipality.”**

This study aims to assess the dietary diversity of lactating mothers and identify factors associated with it in Tarakeswor Municipality. You will be asked questions about your demographic background, socio-economic status, health-related information, dietary practices, knowledge about appropriate nutrition during lactation, food taboos, household food security, and women's empowerment. The interview will take around 25–30 minutes.

There are no risks involved, and there are no direct benefits to you. However, your participation may help improve maternal nutrition programs and benefit other mothers in the future. Your participation is **completely voluntary**, and you can **withdraw at any time**. Your identity will be kept **strictly confidential**, and the information will be used for research purposes only.

**Consent**

I have read and understood the information above and agree voluntarily to participate in the study.

**Signature of the participant**: _______________________
**Name of the Participant**: _______________________
**Date**: _______________________

**Signature of the Witness**: _______________________
**Name of the Witness**: _______________________
**Date**: _______________________

**Signature of the Investigator**: _______________________
**Name of the Investigator: Reshika Rimal**
**Date**: _______________________

**Annex II: Informed Consent (Below 18 Years)**

**Tribhuwan University**
**Maharajgunj Medical Campus**
**Department of Community Medicine and Public Health**

**INFORMATION SHEET**

Namaste, my name is **Reshika Rimal**, a **Bachelor in Public Health student** at Maharajgunj Medical Campus, Institute of Medicine, Tribhuwan University. As part of my academic requirement, I am conducting a study entitled:

**“Dietary diversity and its associated factors among lactating mothers in Tarakeswor Municipality.”**

I kindly request your permission to include your daughter-in-law/wife in this study. She will be asked questions regarding her background, dietary practices, household food security, and more. The interview will take about 25–30 minutes. There are no expected risks or direct benefits, but your support may help guide policies to improve maternal nutrition.

Participation is voluntary. She can stop participating at any time without explanation. Her responses will be kept confidential and used only for research.

**Consent**

I confirm I have read and understood the above, and I agree for my daughter-in-law/wife to participate in the study.

**Signature of Guardian (or Thumbprint)**: _______________________
**Date**: _______________________

**Signature of the Participant**: _______________________
**Name of the Participant**: _______________________
**Date**: _______________________

**Signature of the Witness**: _______________________
**Name of the Witness**: _______________________
**Date**: _______________________

**Signature of the Investigator**: _______________________
**Name of the Investigator: Reshika Rimal**
**Date**: _______________________

#### Questionnaire in English

**Tribhuwan University**

**Maharajgunj Medical Campus, Institute of Medicine Maharajgunj, Kathmandu**

Dietary diversity and its associated factors among Lactating women in Tarakeswor municipality, Baglung: a descriptive cross-sectional community based study

| Instruction: Please tick the appropriate answer and fill in the blank where appropriate. | |
| --- | --- |
| ID NO: | Date of interview: |
| Ward no.: | Time of interview:………………………...  am/pm |

| Q.N  . | Question | Response | Code | Skip/Re marks |
| --- | --- | --- | --- | --- |
| A. **Socio-demographic information** | |  | | |
| A1 | What is your name and cast/ethnicity? |  |  |  |
| A2 | What is your age? |  |  |  |
| A6 | What is your religion? | ☐ Hindu  ☐ Buddhist  ☐ Islam  ☐ Christian  ☐ Others…. | 1  2  3  4  99 |  |
| A7 | Did you ever attend school? | ☐ Yes  ☐ No | Yes=1 No=0 |  |
| A8 | What is the highest level of your education you have completed? | …………………………. |  |  |
| A9 | What is the highest level of education your husband/partner has completed? | ……………………… |  |  |
| A10 | What is your occupation, that is, what kind of work do you mainly do? | ☐ Agriculture  ☐ Service  ☐ Business  ☐ Labor work ☐ Housewife  ☐ Other (specify)…... | 1  2  3  4  5  99 |  |
| A11 | Earning status of the women | ☐ Cash only  ☐ In-kind only  ☐ Both cash and in-kind  ☐ Not paid | 1  2  3  4 |  |
| A12 | What is the major occupation of your husband/partner? | ☐ Agriculture  ☐ Service  ☐ Business | 1  2  3  4 |  |

|  |  | ☐ Labor work ☐ Foreign employment ☐ Other (specify)…... | 5  99 | |  |
| --- | --- | --- | --- | --- | --- |
| A13 | How many members are there in your family? | ..................... |  | |  |
| A14 | What is your type of family? | ☐ Nuclear  ☐ Joint  ☐ Extended | 1  2  3 | |  |
| A15 | Who is the household head of your family? | ☐ Male  ☐ Female | 1  2 | |  |
| **B. Lactating Women health related information** | | | | | |
| B1 | How many of times have you been pregnant (including current pregnancy) | ………………………… |  | |  |
| B2 | Number of miscarriages: | ................................... |  | |  |
| B3 | Number of Children: | ................................... |  | |  |
| B4 | Last menstrual period: | ............................... |  | |  |
| B5 | Did you receive ANC checkup for your pregnancy? | ☐Yes  ☐ No | Yes=1 No=0 | |  |
| B6 | If yes, how many months pregnant were you when you first received antenatal care for this pregnancy | ………………  Don‟t know |  | |  |
| B7 | If yes, how many times did you receive ANC for your pregnancy? | ☐ 1  ☐ 2  ☐ 3  ☐ 4  ☐>4 |  | |  |
| B8 | Are you currently taking iron tablets? | ☐ Yes  ☐ No | 1  0 | |  |
| B9 | Are you currently taking a multivitamin tonic (energy bottle)? | ☐ Yes  ☐ No | 1  0 | |  |
| **C. Socio-economic information** | | | | | |
| C1 | Does your household have?  A television  A mobile telephone?  A landline telephone?  A refrigerator?  A bed?  A sofa?  A cupboard?  A computer? A fan?  A clock  A dhiki/janto | ☐ Yes ☐ No  ☐ Yes ☐ No  ☐ Yes ☐ No  ☐ Yes ☐ No  ☐ Yes ☐ No  ☐ Yes ☐ No  ☐ Yes ☐ No  ☐ Yes ☐ No  ☐ Yes ☐ No  ☐ Yes ☐ No  ☐ Yes ☐ No | Yes=1  No=0 |  | |

| C2 | What type of fuel does your household mainly use for cooking? | ☐ Electricity  ☐ LPG  ☐ Biogas  ☐ Kerosene  ☐ Wood  ☐ Animal dung  ☐ Others….(specify) | 1  2  3  4  5  6  99 |  |
| --- | --- | --- | --- | --- |
| C3 | Do you have a separate room, which is used, as a kitchen? | ☐ Yes  ☐ No | 1  0 |  |
| C4 | Main material of the floor | ☐ Earth/dung  ☐ Cemented  ☐ Ceramic tiles  ☐ Finished floor/parquet  ☐ Other (specify)………… | 1  2  3  4  99 | Observe and write |
| C5 | Main material of the roof | ☐ Thatched roof  ☐ Galvanized sheet  ☐ Ceramic tiles ☐Cement  ☐ Other (specify)………. | 1  2  3  4  99 | Observe and write |
| C6 | Main materials of the exterior walls | ☐Tin  ☐Mud and stone/brick  ☐Wood/bamboo  ☐Concrete  ☐Other (specify)………… | 1  2  3  4  99 | Observe and write |
| C7 | How many rooms in this household are used for sleeping? | ……..rooms |  |  |
| C8 | Does any member of this  household own  A bicycle/ rickshaw?  A motorcycle or motor scooter?  A three wheel tempo?  A tractor?  A car or truck? | ☐ Yes ☐ No  ☐ Yes ☐ No  ☐ Yes ☐ No  ☐ Yes ☐ No  ☐ Yes ☐ No | Yes=1  No=0 |  |
| C9 | Does any member of this household own any agricultural land? | ☐ Yes  ☐ No | 1  0 | If 0= skip to Q.N.  C11 |
| C10 | If yes, how many aana/ropani of agricultural land do members of this household own? | ☐ Aana………… ☐ Ropani………. | 1  2 |  |
| C11 | Does this household own any livestock, herds, other farm animals or poultry? | ☐ Yes  ☐ No | 1  0 |  |
| C12 | How many of the following animals do this household own? | ☐ Buffalo ……………… ☐ Cow and bull ………..  ☐ Goat ………………… |  | If 0= skip to Q.N. C13 |

|  |  | | ☐ Chicken/duck ……….. ☐ Pig ………………….. |  |  | |
| --- | --- | --- | --- | --- | --- | --- |
| C13 | Does your household own any kitchen garden? | | ☐ Yes  ☐ No |  | If 0= skip to Q.N.  D1 | |
| C14 | What have you grown in your kitchen garden? | | ....................  …………………………. |  |  | |
| C15 | Does your kitchen garden product is sufficient for your family consumption? | | ☐ Yes  ☐ No | 1  0 |  | |
| C16 | In the past four weeks, how often did your household bought vegetables for consumption? | | ☐ Never  ☐ Rarely  ☐ Sometimes  ☐ Often | 1  2  3  4 |  | |
| **D. Dietary diversity questionnaire** | | | | | | |
| Now I‟d like to ask you to describe everything that you ate or drank yesterday and the day before yesterday during the day or night, whether you ate it at home or anywhere else. Please include all foods and drinks, any snacks or small meals, as well as any main meals. Remember to include all foods you may have eaten while preparing meals or preparing food for others. Please also include food you ate even if it was eaten elsewhere, away from your home. Also specify the portion of the each food you consumed. Let‟s start with the first food or drink consumed yesterday | | | | | | |
| D1 | | Did you have anything to eat or drink when you woke? | ☐ Yes  ☐ No | Yes=1 No=0 | | If 0= skip to Q.N. D3 |
| D2 | | If yes, what and what was the estimated amount? Anything else?* | ………………………  ……………………….  ………………………... |  | |  |
| D3 | | Did you have anything to eat or drink later in the morning? | ☐ Yes  ☐ No | 1  0 | | If 0= skip to Q.N. D5 |
| D4 | | If yes, what and what was the estimated amount? Anything  else?* | ……………………….. ………………………… ……………………….. |  | |  |
| D5 | | Did you have anything to eat or drink during the afternoon? | ☐ Yes  ☐ No | 1  0 | | If 0= skip to Q.N. D7 |
| D6 | | If yes, what and what was the estimated amount? Anything  else?* | …………………………  …………………………..  ………………………….. |  | |  |
| D7 | | Did you have anything to eat in the evening? | ☐ Yes  ☐ No | 1  0 | | If 0= skip to Q.N. D9 |
| D8 | | If yes, what and what was the estimated amount? Anything  else?* | …………………………. …………………………… …………………………... |  | |  |
| D9 | | Did you have anything else to eat or drink in the evening before going to bed or during | ☐ Yes  ☐ No | 1  0 | | If 0= skip to  Q.N. 1.1 |

|  | the night? |  | | | |  |  |
| --- | --- | --- | --- | --- | --- | --- | --- |
| D10 | If yes, what and what was the estimated amount? Anything  else?* | …………………………… …………………………….  …………………………… | | | |  |  |
|  | Food categories | | How many times have you eaten ___________ in the past 24 hours? | | | | |
|  |  |  | 00=None  01-98=No of times  99=Don‟t know | | | | |
| 1 | **Cereals** | | No. times | of | Amount (portion size) | | |
| 1.1 | Rice bhat/ rice roti | |  |  |  | | |
| 1.2 | Millets (Dhido/roti) | |  |  |  | | |
| 1.3 | Wheat/buckwheat dhido/roti | |  |  |  | | |
| 1.4 | Corn dhido/bhat/roti | |  |  |  | | |
| 1.5 | Porridge, oat bran, oat meal | |  |  |  | | |
| 2 | **White roots and tubers** | |  |  |  | | |
| 2.1 | Potato | |  |  |  | | |
| 2.2 | Yam | |  |  |  | | |
| 2.3 | Turnip | |  |  |  | | |
| 3 | **Pusles (beans, peas and lentils)** | |  |  |  | | |
| 3.1 | Soyabean | |  |  |  | | |
| 3.2 | Chickpea (chana dal) | |  |  |  | | |
| 3.3 | Black gram | |  |  |  | | |
| 3.4 | Green gram | |  |  |  | | |
| 3.5 | French beans | |  |  |  | | |
| 3.6 | Peas (dry) | |  |  |  | | |
| 3.7 | Horse gram | |  |  |  | | |
| 3.8 | Cow Pea | |  |  |  | | |
| 3.9 | Lentil (dal, pulses) | |  |  |  | | |
| 4 | **Nuts and seeds** | |  |  |  | | |
| 4.1 | Almond | |  |  |  | | |
| 4.2 | Peanut, Walnut, cashew | |  |  |  | | |
| 4.3 | Pumpkin seed | |  |  |  | | |
| 4.4 | Sunflower seed | |  |  |  | | |
| 5 | **Milk and milk products** | |  |  |  | | |
| 5.1 | Milk as a drink | |  |  |  | | |
| 5.2 | Yoghurt/Curd | |  |  |  | | |
| 5.3 | Cheese | |  |  |  | | |
| 5.4 | Khuwa (Bhainsiko Dudhko) | |  |  |  | | |
| 5.5 | Paneer | |  |  |  | | |
| 6 | **Meat poultry and fish** | |  |  |  | | |
| 6.1 | Liver, Kidney, heart or other organ meats | |  |  |  | | |
| 6.2 | Chicken or duck | |  |  |  | | |
| 6.3 | Goat | |  |  |  | | |
| 6.4 | Fish | |  |  |  | | |
| 6.5 | Pork | |  |  |  | | |

| 6.6 | Buff |  |  |
| --- | --- | --- | --- |
| 7 | **Eggs** |  |  |
| 7.1 | Chicken egg |  |  |
| 7.2 | Duck egg |  |  |
| 8 | **Dark green leafy vegetables** |  |  |
| 8.1 | Amaranth leaves |  |  |
| 8.2 | Mustard leaves |  |  |
| 8.3 | Rape leaves |  |  |
| 8.4 | Colocasia leaves |  |  |
| 8.5 | Spinach |  |  |
| 8.6 | Broccoli |  |  |
| 8.7 | Coriander leaves |  |  |
| 8.8 | Pumpkin greens |  |  |
| 9 | **Other vitamin A rich vegetables and fruits** |  |  |
| 9.1 | Carrots |  |  |
| 9.2 | Pumpkin |  |  |
| 9.3 | Squash (orange- or dark yellow-fleshed only) |  |  |
| 9.4 | Sweet potato |  |  |
| 9.5 | Mango |  |  |
| 9.6 | Papaya |  |  |
| 9.7 | Peach |  |  |
| 10 | **Other vegetables** |  |  |
| 10.1 | Eggplant / brinjal |  |  |
| 10.2 | Cabbage |  |  |
| 10.3 | Cauliflower |  |  |
| 10.4 | Cucumber |  |  |
| 10.5 | Mushroom |  |  |
| 10.6 | Onion |  |  |
| 10.7 | Radish |  |  |
| 10.8 | Tomato |  |  |
| 10.9 | Okra |  |  |
| 10.10 | Lettuce |  |  |
| 10.11 | Bamboo shoot |  |  |
| 10.12 | Peas/beans (when eaten as fresh pods) |  |  |
| 10.13 | Parwal |  |  |
| 10.14 | Bitter gourd |  |  |
| 11 | **Other fruits** |  |  |
| 11.1 | Apple |  |  |
| 11.2 | Banana |  |  |
| 11.3 | Coconut flesh |  |  |
| 11.4 | Guava |  |  |
| 11.5 | Lemon |  |  |
| 11.6 | Pears |  |  |
| 11.7 | Pomegranate |  |  |
| 11.8 | Pineapple |  |  |
| 12 | **Other processed food** |  |  |

| 12.1 | Biscuits, plain sweets | | |  |  | | | |
| --- | --- | --- | --- | --- | --- | --- | --- | --- |
| 12.2 | Noodles, pasta | | |  |  | | | |
| 12.3 | Chocolate | | |  |  | | | |
| 12.4 | Cake all varieties | | |  |  | | | |
| 12.5 | Ice-cream | | |  |  | | | |
| 12.6 | Cold drinks | | |  |  | | | |
| 12.7 | Momo/ chowmein | | |  |  | | | |
|  | Others | | |  |  | | | |
| Was last day a special day, like a celebration, feast day, fasting, sickness etc in which you ate special food or more or less than usual or did not eat because of fasting? | | | How many times have you eaten ___________ in the past one day? | | | 0=No 1=Yes  9=Don‟t know | | |
| **E. Knowledge on appropriate nutrition during Lactation** | | | | | | | | |
| E1 | How should a Lactation woman eat in comparison to a non lactating woman? | ☐Eat more food each day  ☐Eat more times each day  ☐Eat only what she craves  ☐The same as in non-  Lactation | | | | | 1  2  3  4 | Multipl e  respons  e |
| E2 | Have you heard about iron rich food? | ☐Yes  ☐No | | | | | 1  0 | If 0= skip to Q.N. F4 |
| E3 | If yes, can you tell me the source of iron rich food? | ………………………………  …  ………………………………  … | | | | |  |  |
| E4 | How long should a woman consume iron supplement during lactation ? | ………………………………  …. | | | | |  |  |
| E5 | Have you heard about calcium rich food? | ☐Yes  ☐No | | | | | 1  0 |  |
| E6 | If yes, can you tell me the source of calcium rich food? | ………………………………..  ………………………………  … | | | | |  |  |
| E7 | In your opinion, what type of salt a lactating woman should | ☐Open/ Dhikke salt  ☐Black salt | | | | | 1  2 |  |

|  | consume? | ☐Iodized salt  ☐Others  ☐Don‟t know | | 3  4  5 |  |
| --- | --- | --- | --- | --- | --- |
| E8 | If a lactating woman is undernourished, do you think there will be health risk to the women? | ☐Yes  ☐No | | 1  0 | If 0= skip to Q.N. F8 |
| E9 | If yes, what are the health risks for the women? | ☐Anemia  ☐Pre-eclampsia  ☐Risk of infection  ☐Risk of dying  ☐Others | | 1  2  3  4  5 | Multipl e  respons  e |
| E10 | When a lactating woman is undernourished do you think there will be health risk to the newborn? | ☐Yes  ☐No | | 1  0 | If 0= skip to Q.N.  G1 |
| E11 | If yes, What do you think are the effect of maternal under nutrition on fetal weight? | ☐Low birth weight (small baby) ☐Still birth  ☐No effect on fetal weight  ☐Don‟t know | | 1  2  3  4 |  |
| **F.Information regarding household food security** | | | | | |
| F1 | In the past four weeks, did you worry that your household would not have enough food? | | ☐ Yes  ☐ No | Yes=1 No=0 | If  0=skip to Q G2 |
| F1.1 | How often did this happen? | | ☐ Rarely (1 -2 times)  ☐ Sometimes (3 -10 times)  ☐ Often (>10 times) | 1  2  3 |  |
| F2 | In the past four weeks, were you or any household member not able to eat the kinds of foods you preferred because of a lack of resources? | | ☐ Yes  ☐ No |  | If  0=skip to Q.N.  G3 |
| F2.1 | How often did this happen? | | ☐ Rarely (1 -2 times)  ☐ Sometimes (3 -10 times)  ☐ Often (>10 times) | 1  2  3 |  |
| F3 | In the past four weeks, did you or any household member have to eat a limited variety of foods due to a lack of resources? | | ☐ Yes  ☐ No |  | If  0=skip to Q.N.  G4 |
| F3.1 | How often did this happen? | | ☐ Rarely (1 -2 times)  ☐ Sometimes (3 -10 times)  ☐ Often (>10 times) | 1  2  3 |  |
| F4 | In the past four weeks, did you or any household member have to eat some foods that you really did not want to eat because of a | | ☐ Yes  ☐ No |  | If  0=skip to Q.N.  G5 |

|  | | lack of resources to obtain other types of food? |  |  |  |
| --- | --- | --- | --- | --- | --- |
| G4.1 | | How often did this happen? | ☐ Rarely (1 -2 times)  ☐ Sometimes (3 -10 times)  ☐ Often (>10 times) | 1  2  3 |  |
| G5 | | In the past four weeks, did you or any household member have to eat a smaller meal than you felt you needed because there was not enough food? | ☐ Yes  ☐ No |  | If  0=skip to Q.N.  G6 |
| G5.1 | | How often did this happen? | ☐ Rarely (1 -2 times)  ☐ Sometimes (3 -10 times)  ☐ Often (>10 times) | 1  2  3 |  |
| G6 | | In the past four weeks, did you or any other household member have to eat fewer meals in a day because there was not enough food? | ☐ Yes  ☐ No |  | If  0=skip to Q.N.  G7 |
| G6.1 | | How often did this happen? | ☐ Rarely (1 -2 times)  ☐ Sometimes (3 -10 times)  ☐ Often (>10 times) | 1  2  3 |  |
| G7 | | In the past four weeks, was there ever no food to eat of any kind in your household because of lack of resources to get food? | ☐ Yes  ☐ No |  | If  0=skip to Q.N.  G8 |
| G7.1 | | How often did this happen? | ☐ Rarely (1 -2 times)  ☐ Sometimes (3 -10 times)  ☐ Often (>10 times) | 1  2  3 |  |
| G8 | | In the past four weeks, did you or any household member go to sleep at night hungry because there was not enough food? | ☐ Yes  ☐ No |  | If  0=skip to Q.N.  G9 |
| G8.1 | | How often did this happen? | ☐ Rarely (1 -2 times)  ☐ Sometimes (3 -10 times)  ☐ Often (>10 times) | 1  2  3 |  |
| G9 | | In the past four weeks, did you or any household member go a whole day and night without eating anything because there was not enough food? | ☐ Yes  ☐ No |  | If 0= skip to Q.N.  H1 |
| G9.1 | | How often did this happen? | ☐ Rarely (1 -2 times)  ☐ Sometimes (3 -10 times)  ☐ Often (>10 times) | 1  2  3 |  |
| **H. Information on women’s empowerment** | | | | | |
| H1 | Do you belong to any group?  Please specify.  Mother‟s group | | ☐Yes ☐ No | Yes=1 |  |
|  | Saving/ co-operative group Women‟s group | | ☐ Yes ☐ No  ☐ Yes ☐ No | No=0 |  |
| H2 | Do you own this or any other house either alone or jointly with someone else? | | ☐ Alone only  ☐ Jointly only  ☐ Both alone and jointly  ☐ Does not own | 1  2  3  4 |  |
| H3 | Do you own any land either alone or jointly with someone else? | | ☐ Alone only  ☐ Jointly only  ☐ Both alone and jointly  ☐ Does not own | 1  2  3  4 |  |
| H4 | Who usually makes decisions  about health care for yourself | | ☐ Participant only  ☐ Husband only  ☐ Participant and husband jointly  ☐ Other (specify)…........... | 1  2  3    99 |  |
| H5 | Who usually makes decisions about making major household purchases? | | ☐ Participant only  ☐ Husband only  ☐ Participant and husband jointly  ☐ Other (specify)…........... | 1  2  3    99 |  |
| H6 | Who usually makes decisions about visits to your family or relatives? | | ☐ Participant only  ☐ Husband only  ☐ Participant and husband jointly  ☐ Other (specify)…........... | 1  2  3    99 |  |

“Thank you for your valuable time, information and cooperation”
