# Supplementary material for: Maternal dietary diversity and its correlates in a semi-urban municipality of Nepal: A cross-sectional study
Source: PLOS Glob Public Health. 2026 Aug 25;6(8):e0005711. doi: 10.1371/journal.pgph.0005711 (PMC13505919; doi:10.1371/journal.pgph.0005711)
Supplement: Supplementary file 4 [file pgph.0005711.s004.docx]

**STROBE Statement**—checklist of items that should be included in reports of observational studies

|  | Item No. | Recommendation | Page  No. | | Relevant text from manuscript | |
| --- | --- | --- | --- | --- | --- | --- |
| **Title and abstract** | 1,2,3 | (*a*) Indicate the study’s design with a commonly used term in the title or the abstract | 1 | | “Maternal Dietary Diversity and Socioeconomic Determinants Among Lactating Mothers in a Semi-Urban Municipality of Nepal: A Cross-Sectional Study” | |
|  |  | (*b*) Provide in the abstract an informative and balanced summary of what was done and what was found | 2,3 | | The abstract summarizes the background, study objectives, methods, key results, and conclusion, providing a clear overview of the research. | |
| Introduction | | | | |  | |
| Background/rationale | 2 | Explain the scientific background and rationale for the investigation being reported | 4,5,6 | | The introduction highlights the nutritional vulnerability of lactating mothers, the importance of dietary diversity, and gaps in current research, especially in semi-urban Nepalese settings. | |
| Objectives | 3 | State specific objectives, including any prespecified hypotheses 2 | 5 | | This study aimed to assess the prevalence of minimum dietary diversity and identify associated sociodemographic, economic, and household-level determinants, including food security, among lactating mothers in a semi-urban municipality of Nepal. By addressing this gap, we aim to inform locally relevant and equity-driven interventions to improve maternal nutrition in rapidly urbanizing LMIC contexts. | |
| Methods | | | | |  | |
| Study design | 5 | Present key elements of study design early in the paper | 6 | | “A descriptive cross-sectional community-based study was conducted among lactating mothers residing in Tarakeswor Municipality.” | |
| Setting | 5 | Describe the setting, locations, and relevant dates, including periods of recruitment, exposure, follow-up, and data collection | 6 | | “The study was conducted in Tarakeswor Municipality, Kathmandu District, Nepal. Data collection was carried out from **March to May 2023**.” | |
| Participants | 6 | *Cross-sectional study*—Give the eligibility criteria, and the sources and methods of selection of participants | 8 | | “Lactating mothers aged 15–49 years with a child under 12 months, residing in Tarakeswor Municipality for at least the past 6 months, and willing to participate were included. A systematic random sampling method was used to select participants.” | |
|  |  |  | |  | |  |
| Variables | 7 | Clearly define all outcomes, exposures, predictors, potential confounders, and effect modifiers. Give diagnostic criteria, if applicable | 9,10,11 | | Outcome: Minimum dietary diversity (MDD-W); Exposure variables: maternal education, occupation, household food insecurity, empowerment; Confounders: age, income, family size. Effect modifiers not applicable. | |
| Data sources/ measurement | 8 | For each variable of interest, give sources of data and details of methods of assessment (measurement). Describe comparability of assessment methods if there is more than one group | *4 9,10,11,12* | | Data were collected using an interviewer-administered semi-structured questionnaire based on FAO's MDD-W tool and HFIAS indicators. Information on socio-demographics, dietary intake (24-hour recall), and knowledge was obtained through direct interviews. | |
| Bias | 9 | Describe any efforts to address potential sources of bias | 9 | | Pretesting of tools was done to reduce information bias. Standard training was provided to data collectors. Systematic random sampling minimized selection bias. | |
| Study size | 10 | Explain how the study size was arrived at | 7 | | Sample size (n = 251) was calculated using a single population proportion formula with an estimated dietary diversity prevalence of 60%, 95% confidence level, and 5% margin of error. | |

Continued on next page

| Quantitative variables | 11 | Explain how quantitative variables were handled in the analyses. If applicable, describe which groupings were chosen and why | 9 | Quantitative variables such as age and dietary diversity score were categorized. Dietary diversity was categorized based on MDD-W threshold: ≥5 food groups = adequate, <5 = inadequate. |
| --- | --- | --- | --- | --- |
| Statistical methods | 12 | (*a*) Describe all statistical methods, including those used to control for confounding | 5 | Descriptive statistics were used to summarize variables. Bivariate analysis (Chi-square test) identified associations. Variables with p<0.2 were entered into a multivariable logistic regression model to control for confounding. |
|  |  | (*b*) Describe any methods used to examine subgroups and interactions | 5 | Subgroup analysis was not performed. Interactions were not assessed |
|  |  | (*c*) Explain how missing data were addressed | 5 | Complete-case analysis was conducted. Records with missing key variables were excluded. |
|  |  | (*d*) *Cohort study*—If applicable, explain how loss to follow-up was addressed  *Case-control study*—If applicable, explain how matching of cases and controls was addressed  *Cross-sectional study*—If applicable, describe analytical methods taking account of sampling strategy | 3,5 | Analytical methods accounted for the study’s stratified random sampling approach by ensuring proportional representation across selected wards. Descriptive statistics were stratified by key sociodemographic factors, and multivariable logistic regression was conducted to assess associations between independent variables and minimum dietary diversity. Variables were selected based on theoretical relevance and bivariate significance (p < 0.20). Variance inflation factor (VIF) was assessed to check for multicollinearity, and model fit was evaluated using Hosmer-Lemeshow goodness-of-fit tests. Although weights were not applied, the sampling strategy was carefully designed to enhance internal validity and representativeness of the target population within the municipality. |
|  |  | (*e*) Describe any sensitivity analyses |  |  |
| Results | | | | |
| Participants | 13* | (a) Report numbers of individuals at each stage of study—eg numbers potentially eligible, examined for eligibility, confirmed eligible, included in the study, completing follow-up, and analysed | 7,8 | A total of 260 women were approached, 251 consented and were included in the final analysis. |
|  |  | (b) Give reasons for non-participation at each stage | 8 | Nine eligible participants declined due to personal reasons or lack of time. |
|  |  | (c) Consider use of a flow diagram |  | None |
| Descriptive data | 14* | (a) Give characteristics of study participants (eg demographic, clinical, social) and information on exposures and potential confounders | 13 | Table 2 presents demographic characteristics, education, occupation, food security status, etc. |
|  |  | (b) Indicate number of participants with missing data for each variable of interest | 7,8 | Variables with missing data were excluded from analysis; missing data not reported per variable. |
|  |  | (c) *Cohort study*—Summarise follow-up time (eg, average and total amount) |  |  |
| Outcome data | 15* | *Cohort study*—Report numbers of outcome events or summary measures over time |  |  |
|  |  | *Case-control study—*Report numbers in each exposure category, or summary measures of exposure |  |  |
|  |  | *Cross-sectional study—*Report numbers of outcome events or summary measures | *6* | Around 58% of mothers met minimum dietary diversity. Outcome variable summarized in Table 2. |
| Main results | 16 | (*a*) Give unadjusted estimates and, if applicable, confounder-adjusted estimates and their precision (eg, 95% confidence interval). Make clear which confounders were adjusted for and why they were included | 18 | Multivariable logistic regression results are reported with adjusted odds ratios and 95% CIs. Confounders like maternal education and food security were adjusted for. |
|  |  | (*b*) Report category boundaries when continuous variables were categorized | 14 | Age grouped into <25 and ≥25 years; dietary diversity categorized as <5 and ≥5 food groups. |
|  |  | (*c*) If relevant, consider translating estimates of relative risk into absolute risk for a meaningful time period |  | Not applicable |

Continued on next page

| Other analyses | 17 | Report other analyses done—eg analyses of subgroups and interactions, and sensitivity analyses |  | No subgroup or sensitivity analyses were performed. |
| --- | --- | --- | --- | --- |
| Discussion | | | | |
| Key results | 18 | Summarise key results with reference to study objectives | 16 | “The study found that only 58% of lactating mothers achieved minimum dietary diversity. Maternal education, food security, and household decision-making power were significantly associated with dietary diversity.” |
| Limitations | 19 | Discuss limitations of the study, taking into account sources of potential bias or imprecision. Discuss both direction and magnitude of any potential bias | 21 | “The study's limitations include potential recall bias from 24-hour recall data, social desirability bias during interviews, and lack of seasonal dietary variation consideration. These may over- or underestimate dietary diversity scores.” |
| Interpretation | 20 | Give a cautious overall interpretation of results considering objectives, limitations, multiplicity of analyses, results from similar studies, and other relevant evidence | 9 | “Findings align with similar studies in Nepal and South Asia, emphasizing the importance of food security and maternal education. Despite limitations, the results support strengthening maternal nutrition interventions in semi-urban settings.” |
| Generalisability | 21 | Discuss the generalisability (external validity) of the study results | 9 | “Findings may be generalized to lactating mothers in other semi-urban Nepalese settings with similar socio-cultural and economic contexts.” |
| Other information | |  | | |
| Funding | 22 | Give the source of funding and the role of the funders for the present study and, if applicable, for the original study on which the present article is based |  | “This research was conducted as a partial fulfillment of the Bachelor of Public Health program at Tribhuvan University. No external funding was received. The university had no role in the design, data collection, analysis, or manuscript preparation.” |

*Give information separately for cases and controls in case-control studies and, if applicable, for exposed and unexposed groups in cohort and cross-sectional studies.

**Note:** An Explanation and Elaboration article discusses each checklist item and gives methodological background and published examples of transparent reporting. The STROBE checklist is best used in conjunction with this article (freely available on the Web sites of PLoS Medicine at http://www.plosmedicine.org/, Annals of Internal Medicine at http://www.annals.org/, and Epidemiology at http://www.epidem.com/). Information on the STROBE Initiative is available at www.strobe-statement.org.
