# Supplementary material for: Maternal dietary diversity and its correlates in a semi-urban municipality of Nepal: A cross-sectional study
Source: PLOS Glob Public Health. 2026 Aug 25;6(8):e0005711. doi: 10.1371/journal.pgph.0005711 (PMC13505919; doi:10.1371/journal.pgph.0005711)
Supplement: Supplementary file 5 [file pgph.0005711.s006.docx]

Section by Section Review:

Title and Abstract

Strengths: Title is clear, consise, and reflects the study's scope. Abstract summarizes objectives, methods, and key findings well.

Weakness: Abstract could emphasize the 'policy implication' of maternal dietary diversity' more strongly.

Recommendation: Add a sentence highlighting how findings can inform nutrition programs in semi-urban Nepal.

Introduction

Strengths: Provides strong rationale, situated the study within global maternal nutrition challenges, and cities relevant literature.

Weakness: Background could integrate more recent global evidence on dietary diversity interventions.

Recommendations: Expand discussion of how semi-urban contexts differ from rural/urban and why this matters for policy. Is there any operational definition of semi-urban ??

Methods

Strengths: Clear description of study design, sampling, and used of validated tools.

Weakness: Limited detail on how cultural dietary taboos were accounted for. As there were more explanation of relation between dietary diversity and cultural taboos in introduction.

Recommendations: Clarify potential biases (e.g. recall bias in 24-hour dietary recall) and how they were minimized.

Results

Strength: Results are well-presented with tables and clear statistics. Strong use of adjusted odds ratios.

Weakness: Some findings (e.g. low consumption of Vitamin A-rich foods) could be contextualized more deeply.

Recommendation: Highlight implications of "hidden hunger" and micronutrient gaps despite overall dietary diversity.

Discussion

Strengths: Thoughtful interpretation of socio-economic determinants.

Weakness: Limitation section is brief. It would be better if seasonal variation and cultural practices given more emphasis.

Recommentation: Expand on how interventions can address both structural (wealth, food security) and behavioral (knowledge, taboos) barriers.

Conclusion

Strength: Aligns with findings and emphasis equity-focused interventions

Weakness: Could be more action-oriented, suggesting specific strategies for policymakers.
