# Supplementary material for: Maternal dietary diversity and its correlates in a semi-urban municipality of Nepal: A cross-sectional study
Source: PLOS Glob Public Health. 2026 Aug 25;6(8):e0005711. doi: 10.1371/journal.pgph.0005711 (PMC13505919; doi:10.1371/journal.pgph.0005711)
Supplement: Supplementary file 6 [file pgph.0005711.s008.docx]

**Response to Reviewers**

Dear Editor and Reviewers,

Thank you for the opportunity to revise our manuscript titled **“**Maternal dietary diversity and its correlates in a semi-urban municipality of Nepal: A cross-sectional study. We sincerely appreciate the insightful comments and suggestions provided by the editor and reviewers. These comments have helped us improve the clarity and policy relevance of the manuscript.

We have carefully revised the manuscript and addressed each comment point-by-point. All changes have been incorporated into the revised manuscript and highlighted in the tracked-changes version.

Below we provide detailed responses to each comment

| **Reviewer Comment** | **Author Response** |
| --- | --- |
| **Title and Abstract:** The abstract is clear but could emphasize policy implications of maternal dietary diversity more strongly. | Thank you for this helpful suggestion. We have revised the Abstract to more clearly emphasize the policy implications of our findings for maternal nutrition programs in Nepal. Specifically, we added a sentence highlighting the importance of strengthening nutrition education, improving household food security, and integrating dietary diversity counseling into antenatal and postnatal care services in semi-urban municipalities (Abstract, Page 2, Lines 29–31). |
| **Introduction:** Expand discussion of how semi-urban contexts differ from rural and urban areas and why this matters for policy. Provide an operational definition of semi-urban. | Thank you for this valuable suggestion. We have revised the Introduction to provide a clearer description of semi-urban contexts and how they differ from rural and fully urban environments. Specifically, we added text explaining that semi-urban areas represent transitional settings characterized by mixed livelihoods, where households often rely on both subsistence agriculture and wage-based employment while experiencing increasing exposure to urban markets, infrastructure, and services. We also highlight how these areas often have hybrid food environments that combine elements of both rural and urban food systems, which can influence access to diverse and nutritious foods. This clarification strengthens the policy relevance of the study by explaining why semi-urban settings are important contexts for maternal nutrition interventions. The revised text has been incorporated in the Introduction (Introduction, Page 4-5, Lines 76–86). |
| **Introduction:** Include more recent global evidence on dietary diversity interventions. | Thank you for this helpful suggestion. We have revised the Introduction to incorporate recent global evidence on interventions that improve dietary diversity among women in low- and middle-income countries. Specifically, we added discussion of behavior change communication, nutrition education, women’s empowerment programs, and nutrition-sensitive interventions such as conditional cash transfers, food voucher programs, and integrated maternal health services, which have demonstrated positive effects on dietary diversity and household food security. These additions strengthen the global context of the study and highlight evidence-based strategies relevant for maternal nutrition policies in Nepal. The revised text has been incorporated in the Introduction (Introduction, Page 3-4, Lines 55–63). |
| **Methods:** Clarify how cultural dietary taboos were accounted for in the study. | Thank you for this important comment. We have revised the Study Procedure subsection of the Methods to clarify how cultural dietary practices were considered during questionnaire development and pretesting. Specifically, we added text explaining that during the development and translation process, attention was given to locally relevant food practices and commonly reported postpartum dietary restrictions to ensure that questions on dietary intake were culturally appropriate and clearly understood by participants. In addition, the pretesting process helped ensure that culturally specific dietary practices or food avoidance behaviors did not affect the interpretation of dietary recall questions. This clarification has been incorporated in the Methods section (Page 12, Lines 219–227). |
| **Methods:** Clarify potential biases such as recall bias in the 24-hour dietary recall and how they were minimized. | Thank you for this valuable suggestion. We have revised the Methods section to clarify steps taken to minimize potential recall bias associated with the 24-hour dietary recall. Specifically, trained interviewers used probing questions and locally familiar food examples to assist participants in accurately recalling foods and beverages consumed during the previous day. Standardized interviewing procedures were also followed to improve recall accuracy. This clarification has been added to the Study Procedure subsection of the Methods (Page 13, Lines 230–235). |
| **Results:** Some findings such as low consumption of Vitamin A-rich foods could be contextualized further. | Thank you for this helpful suggestion. To maintain the appropriate structure of the manuscript, explanatory sentences discussing the implications of low consumption of vitamin A–rich foods were removed from the Results section. The contextual interpretation of this finding has been incorporated into the Discussion section, where we highlight that low intake of nutrient-dense foods such as eggs, nuts and seeds, and vitamin A-rich fruits and vegetables may indicate potential micronutrient gaps and a risk of “hidden hunger” among lactating mothers (Page 23, Lines 360–367). |
| **Results:** Highlight implications of hidden hunger and micronutrient gaps. | Thank you for this helpful suggestion. We have revised the Results section to more clearly highlight the implications of the low consumption of micronutrient-rich foods observed in the study. Specifically, we added a sentence noting that, although most mothers met the minimum dietary diversity threshold, the low intake of nutrient-dense foods such as eggs, nuts and seeds, and vitamin A–rich fruits and vegetables suggests potential micronutrient gaps and a possible risk of “hidden hunger” among lactating mothers. To maintain the appropriate structure of the manuscript, explanatory interpretation of this finding was removed from the Results section and incorporated into the Discussion. The revised text has been included in the Results (Page 18, Lines 296–299) and further discussed in the Discussion (Page 23, Lines 360–367). |
| **Discussion:** The limitation section is brief. Seasonal variation and cultural practices should be emphasized more. | Thank you for this helpful suggestion. We have revised and expanded the Strengths and Limitations section to further emphasize the potential influence of seasonal variation and cultural dietary practices on maternal dietary diversity. Specifically, we added text noting that dietary patterns in Nepal may vary across seasons due to fluctuations in the availability and affordability of fruits, vegetables, and other nutrient-rich foods, and that the single time-point data collection may not fully capture this variation. We also clarified that cultural dietary practices, including postpartum food taboos that may influence maternal food choices, were not quantitatively assessed in this study. Additionally, we noted that because the study was conducted in a single semi-urban municipality, the findings may not be generalizable to other ecological or cultural contexts in Nepal. These revisions have been incorporated in the Strengths and Limitations section (Page 28, Lines 468–475). |
| **Discussion:** Expand discussion of interventions addressing both structural (wealth, food security) and behavioral (knowledge, taboos) barriers. | Thank you for this valuable suggestion. We have expanded the Discussion section to more clearly describe interventions addressing both structural and behavioral barriers to improving maternal dietary diversity. Specifically, we added text discussing structural approaches such as strengthening household food security through social protection programs, livelihood support for women, and improved access to affordable nutrient-rich foods through local markets and agricultural initiatives. We also elaborated on behavioral strategies, including nutrition education, counseling during antenatal and postnatal care, and community-based awareness programs to address knowledge gaps and culturally rooted food taboos that influence maternal diets. These additions help provide a more comprehensive interpretation of the findings and highlight potential policy and programmatic responses. The revisions have been incorporated in the Discussion section (Page 26, Lines 414–423). |
| **Conclusion:** The conclusion could be more action-oriented and suggest strategies for policymakers. | Thank you for this helpful suggestion. We have revised the Conclusion section to make it more action-oriented and to highlight practical strategies for policymakers. Specifically, we added text emphasizing the need to integrate maternal nutrition education into routine maternal health services, expand social protection and food security programs for vulnerable households, and promote community-based initiatives that improve women’s access to affordable, nutrient-rich foods in rapidly urbanizing municipalities. These revisions strengthen the policy relevance of the study and provide clearer recommendations for improving maternal nutrition in semi-urban settings. The revised text has been incorporated in the Conclusion section (Page 27, Lines 447–450). |
