# Supplementary material for: Maternal dietary diversity and its correlates in a semi-urban municipality of Nepal: A cross-sectional study
Source: PLOS Glob Public Health. 2026 Aug 25;6(8):e0005711. doi: 10.1371/journal.pgph.0005711 (PMC13505919; doi:10.1371/journal.pgph.0005711)
Supplement: Supplementary file 7 [file pgph.0005711.s009.docx]

#Reviewer 3 comments:

Comment 1: Sample Size Inconsistency and Population Correction

Response:
Thank you for this important observation. We agree that the distinction between the population estimate used for sample size calculation and the final sampling frame was not sufficiently clear in the original manuscript. To address this concern, we have added a clarification in the “Sample Size and Sampling Method” section. Specifically, we now state that the finite population correction was based on the estimated source population available from municipal records during the study planning phase (N = 2500). We further clarified that subsequent field enumeration conducted with the support of Female Community Health Volunteers (FCHVs) and local health facilities identified 700 eligible lactating mothers across the municipality, which constituted the final sampling frame for participant selection. These revisions have been incorporated into the Methods section (Lines 145–149).

Comment 2: Operationalization of Women's Empowerment

Response:
Thank you for this valuable comment. We agree that women’s empowerment is a multidimensional and context-dependent construct. To address this concern, we revised the Methods section (Lines 221–223) to clarify the rationale for dichotomizing the empowerment variable, noting that this approach was adopted to facilitate statistical analysis and due to limited variability across individual decision-making domains. We also revised the Strengths and Limitations section (Lines 479–484) to acknowledge that the dichotomous measure may not fully capture the multidimensional and context-specific nature of women’s empowerment. We appreciate the suggestion to report individual empowerment domains separately; however, empowerment was operationalized as a composite variable for the primary analysis, and therefore the summary measure was retained.

Comment 3: Cross-sectional Design and Causal Language

Response:
Thank you for this comment. We agree that causal interpretations should be avoided in a cross-sectional study. Accordingly, we revised the Discussion (Lines 364–365, 379–385, 391, 403, 413–419, 432–440, and 445–446) and Conclusion (Lines 458–472) to use more cautious language and ensure that findings are presented as associations rather than causal relationships.

Comment 4: Dietary Assessment Limitations

Response:
Thank you for this valuable comment. We have expanded the Strengths and Limitations section to provide a more comprehensive discussion of the limitations of the dietary assessment method. Specifically, we now acknowledge the potential influence of day-to-day dietary variation and the inability of a single 24-hour recall to quantitatively assess nutrient adequacy (Lines 499–501). We also clarified the potential effects of seasonal variation (Lines 502–505) and social desirability bias associated with self-reported measures, including dietary intake (Lines 509–510).

Comment 5: Potential Residual Confounding

Response:
Thank you for this important observation. We agree that residual confounding may be present because some potentially relevant variables were not assessed in this study. We have revised the Strengths and Limitations section (Lines 507–511) to explicitly acknowledge that factors such as maternal BMI, postpartum duration, breastfeeding intensity, household food expenditure, maternal morbidity, and specific cultural dietary practices were not included in the analysis and may have contributed to residual confounding.

Comment 6: Interpretation of "Hidden Hunger"

Response:
Thank you for this valuable comment. We agree that the term "hidden hunger" may imply confirmed micronutrient deficiencies, which were not directly assessed in this study. Accordingly, we revised the Discussion section (Lines 364–366) and replaced the term with more cautious wording, stating that the observed dietary pattern "may suggest potential micronutrient inadequacies."

Comment 7: ANC Visits and Statistical Interpretation

Response:
Thank you for this important observation. We agree that the association between ANC visits and dietary diversity should be interpreted cautiously given the borderline confidence interval. We revised the Discussion section (Lines 418–425) to acknowledge the marginal statistical significance of the association and to avoid presenting ANC attendance as a definitive predictor of dietary diversity.

Comment 8: Clarify Time Period of Data Collection

Response:
Thank you for identifying this inconsistency. We reviewed and revised the Methods section (Lines 115–117) to ensure consistency regarding the study period and data collection dates. The manuscript now clearly states that the study was conducted between October 2020 and March 2021, with data collection and participant recruitment taking place from 09/10/2020 to 24/03/2021.

Comment 9: Food Group Description

Response:
Thank you for this helpful suggestion. We revised the Methods section (Lines 181–186) to present the ten MDD-W food groups more clearly and consistently according to the FAO classification. The food groups are now listed separately using standard MDD-W terminology.

Comment 10: Terminology Consistency

Response:
Thank you for this suggestion. We reviewed the manuscript and confirmed that the terminology is used consistently throughout. “MDD-W” is used when referring to the Minimum Dietary Diversity for Women indicator, while “DDS” refers to the Dietary Diversity Score. We also performed a final review to ensure consistency in the use of these terms across the manuscript.

Comment 11: Repetition in Discussion

Response:
Thank you for this helpful suggestion. We reviewed the Discussion section and condensed repetitive statements related to semi-urban transition, socioeconomic determinants, and the broader LMIC context to improve readability while preserving the interpretation of key findings. Relevant revisions were made in the Discussion section (Lines 373–375 and 464–465).

Comment 12:Reference Formatting

Thank you for this careful review. We thoroughly examined the reference list and revised it to improve consistency, completeness, and adherence to PLOS formatting requirements. Specifically, we corrected incomplete citations, added missing bibliographic details where available (including page ranges, publication information, and access dates for web-based sources), standardized capitalization and journal formatting, and revised organizational references to ensure consistent presentation. In addition, we reviewed all URLs and updated citation formatting throughout the reference list to align with the journal’s guidelines.

#Reviewer 4:

Comment 1: How do the dietary diversity and health outcomes of older lactating mothers differ from their younger counterparts, and what are the implications for public health interventions? Furthermore, the significant proportion of participants belonging to upper-caste groups and identifying as Hindu warrants an examination of the potential impact of sociocultural factors on dietary diversity and health outcomes. Specifically, what role do caste and religious affiliations play in shaping food choices and access to healthcare services in this semi-urban setting?

Response:
Thank you for this insightful comment. The primary objective of this study was to identify factors associated with minimum dietary diversity among lactating mothers rather than conduct detailed subgroup analyses by age, caste, or religion. The study did not assess maternal or infant health outcomes; therefore, comparisons of health outcomes between older and younger lactating mothers were beyond the scope of the present analysis. Nevertheless, maternal age may influence dietary practices through differences in experience, autonomy, and socioeconomic circumstances and merits further investigation in future studies.

Regarding sociocultural factors, caste/ethnicity and religion were examined as sociodemographic variables; however, they were not retained as significant predictors in the final multivariable model. We acknowledge that sociocultural factors may still influence maternal dietary practices through cultural food preferences, dietary restrictions, household norms, and health-seeking behaviors. To address this point, we added a statement in the Discussion section (Lines 453–456) acknowledging the potential influence of these factors and recommending further research using qualitative or mixed-methods approaches.

Comment 2: The study's findings on household characteristics, including the high percentage of male-headed households and land ownership, also merit closer examination. How do these factors influence the decision-making power and autonomy of lactating mothers in terms of their dietary choices and healthcare utilization? Additionally, the relatively even wealth distribution across quintiles may mask underlying inequalities in access to resources and healthcare services. What are the underlying mechanisms driving the observed patterns of healthcare utilization, including the low percentage of mothers who completed four or more antenatal care visits? A more nuanced analysis of the interplay between wealth, education, and empowerment is necessary to understand the complex dynamics at play. For example, how do the 43% of women categorized as empowered differ from their non-empowered counterparts in terms of their dietary diversity, healthcare utilization, and overall health outcomes?

Response:
Thank you for this thoughtful comment. The primary objective of this study was to identify factors associated with minimum dietary diversity among lactating mothers rather than to conduct detailed pathway or interaction analyses examining the relationships among household characteristics, empowerment, wealth, education, and healthcare utilization. While male-headed households, land ownership, wealth, education, and women’s empowerment may influence dietary practices and healthcare utilization through complex social and economic mechanisms, a comprehensive examination of these pathways was beyond the scope of the present study.

Nevertheless, we agree that decision-making autonomy, access to resources, and socioeconomic position are important determinants of maternal nutrition and health service utilization. We have expanded the Discussion section to acknowledge the potential interplay among these factors and recommend future studies using larger samples and mixed-methods approaches to further explore these relationships. The study did not assess maternal health outcomes; therefore, comparisons of overall health outcomes between empowered and non-empowered women were beyond the scope of the present analysis.

Comment 3: What are the key factors contributing to the observed mean dietary diversity score of 5.03, and how do these factors vary across different socioeconomic and demographic subgroups? Moreover, the finding that only 23.1% of mothers completed four or more antenatal care visits highlights a critical gap in healthcare utilization. What are the underlying barriers to accessing antenatal care services, and how can these be addressed through targeted interventions? A more detailed analysis of the relationships between dietary diversity, healthcare utilization, and health outcomes is necessary to inform evidence-based policy and programmatic interventions. For instance, how do the dietary diversity scores of lactating mothers relate to their nutritional status, and what are the implications for the health and well-being of both mothers and their infants? A deeper understanding of the complex interplay between sociocultural, economic, and environmental factors is necessary to develop effective strategies for promoting maternal dietary diversity and improving health outcomes in this semi-urban municipality of Nepal.

Thank you for this valuable comment. The factors associated with dietary diversity were examined through multivariable logistic regression, which identified maternal education, employment status, household wealth, food security, family structure, nutrition knowledge, and antenatal care utilization as important factors associated with achieving minimum dietary diversity. However, detailed subgroup analyses across socioeconomic and demographic groups were beyond the scope of the present study.We agree that the low proportion of mothers completing four or more antenatal care visits warrants attention. Although the present study did not specifically investigate barriers to antenatal care utilization, factors such as socioeconomic constraints, limited awareness, accessibility challenges, and competing household responsibilities may contribute and should be explored in future research. In addition, this study did not collect data on maternal nutritional status, micronutrient biomarkers, infant health outcomes, or detailed environmental determinants. Therefore, the relationships between dietary diversity, nutritional status, and health outcomes could not be directly assessed. We have emphasized in the Discussion that maternal dietary diversity is influenced by a complex interplay of socioeconomic, behavioral, and healthcare-related factors and that future longitudinal and mixed-methods studies are needed to better understand these relationships and inform targeted interventions.

Comment 4: The study's reliance on a cross-sectional design limits its ability to establish causal relationships between the predictor variables and maternal dietary diversity. How do the authors plan to address potential temporal relationships between these variables? Moreover, the sample size of 251 lactating mothers, although adequate for descriptive analysis, may not be sufficient to detect subtle yet significant correlations between the variables of interest. Did the authors conduct a priori power calculations to determine the required sample size?

Response:
Thank you for this comment. We acknowledge that the cross-sectional design limits the ability to establish temporal or causal relationships, and this has been noted in the Strengths and Limitations section. Regarding sample size, an a priori sample size calculation was performed using the single population proportion formula, with finite population correction and adjustment for non-response. Details have been clarified in the Methods section.

Comment 5: The demographic characteristics of the study population, as presented in Table 2, reveal a notable skewness in the age distribution, with 69.7% of participants falling within the 18-25 year age range. How do the authors explain this uneven distribution, and what implications might it have on the representativeness of the sample? Furthermore, the dichotomization of certain variables, such as household food security and empowerment, may oversimplify the complexities of these constructs. Would the authors consider using more nuanced, continuous measures of these variables?

Response:
Thank you for this valuable comment. The age distribution observed in the study reflects the characteristics of the sampled population of lactating mothers in Tarakeswor Municipality during the study period. Participants were selected through proportionate stratified random sampling, which supports the representativeness of the sample. We agree that dichotomization of food security and empowerment may not fully capture the complexity of these constructs. Accordingly, we have clarified the rationale for dichotomization in the Methods section and acknowledged this limitation in the Strengths and Limitations section.

Comment 6: The study's use of a dietary diversity score, with a mean of 5.03 (SD ± 1.25), raises questions about the construct validity of this measure. How did the authors select the specific food groups included in the score, and what evidence supports the chosen cut-off point for minimum dietary diversity? Additionally, the lack of information about the timing and frequency of dietary assessments may introduce recall bias and limit the accuracy of the dietary data. Can the authors provide more details about their dietary assessment methodology and how they minimized potential sources of error?

Response:
Thank you for this comment. Dietary diversity was assessed using the internationally validated Minimum Dietary Diversity for Women (MDD-W) indicator, which comprises 10 FAO-defined food groups. We have revised the Methods section to more clearly describe the food group classifications and the rationale for the MDD-W cutoff (≥5 food groups). We also clarified the dietary assessment methodology and expanded the Strengths and Limitations section to acknowledge the potential influence of recall bias and other limitations associated with a single 24-hour dietary recall.

Comment 7: The findings regarding the predictors of maternal dietary diversity, such as education and employment status, are intriguing but require further exploration. How do the authors propose to tease apart the relative contributions of these variables, given the high correlation between education and employment status? Moreover, the study's results indicate that only 23.1% of participants completed four or more antenatal care visits, which is concerning given the importance of ANC in promoting maternal and child health. What strategies do the authors suggest for improving ANC utilization and, by extension, maternal dietary diversity in this population?

Response:
Thank you for this insightful comment. Education and employment are closely related socioeconomic factors, and their independent contributions may be difficult to fully disentangle in a cross-sectional study. Although both variables were included in the multivariable model, further research using longitudinal designs may help better understand their relative contributions to maternal dietary diversity. Regarding antenatal care utilization, we agree that the low proportion of mothers completing four or more ANC visits is an important public health concern. Strengthening nutrition education and counseling during maternal health services, improving awareness of ANC benefits, and addressing barriers to healthcare access may help improve ANC utilization and maternal dietary diversity.

Comment 8: “Recent global evidence suggests that targeted nutrition interventions can improve women’s dietary diversity in low- and middle-income countries” Add the following reference:
2025.Digital technology to advance global health by reviewing nutrition interventions and assessing needs in urban Syria. Discover Food 8, 416.

Response:
Thank you for the suggested reference. We reviewed the recommended article and appreciate its relevance to nutrition interventions in low- and middle-income settings. However, we retained references that were more directly related to maternal nutrition and dietary diversity among women of reproductive age, which are more closely aligned with the focus of the present study.

Comment 9: The manuscript's discussion of the study's implications for public health policy and practice is somewhat cursory. How do the authors envision their findings informing the development of targeted interventions to enhance maternal dietary diversity and improve health outcomes in this and similar populations? What specific recommendations do they propose for policymakers, healthcare providers, and other stakeholders to address the complex factors influencing maternal dietary diversity in this context?

Response:
Thank you for this valuable comment. We agree that translating research findings into actionable recommendations is important. The Discussion and Conclusion sections have been strengthened to further emphasize the implications of our findings for policy and practice. Specifically, we highlight the importance of strengthening maternal nutrition education and counseling through antenatal and postnatal care services, improving household food security through social protection and livelihood support programs, promoting women’s education and economic participation, and enhancing access to affordable nutrient-rich foods. We also emphasize the need for multi-sectoral collaboration among policymakers, healthcare providers, and community stakeholders to address the socioeconomic and behavioral factors associated with maternal dietary diversity.

Comment 10: The authors employed the Minimum Dietary Diversity for Women (MDD-W) indicator to assess dietary diversity among lactating mothers. However, it is essential to acknowledge that this indicator has been criticized for its limited ability to capture the nuances of dietary diversity, particularly in resource-poor settings. The MDD-W indicator focuses on the consumption of 10 predefined food groups, which may not accurately reflect the dietary habits of lactating mothers in Nepal. I would like to know whether the authors considered using alternative indicators, such as the Dietary Diversity Score (DDS) or the Food Variety Score (FVS), which may provide a more comprehensive understanding of dietary diversity.

Thank you for this important comment. We selected the Minimum Dietary Diversity for Women (MDD-W) indicator because it is an internationally validated and widely recommended measure for assessing dietary diversity and micronutrient adequacy among women of reproductive age, including lactating mothers. The MDD-W framework has been extensively used in global and national nutrition research, allowing comparability across studies and settings. We acknowledge that no single indicator can fully capture all dimensions of dietary intake. Dietary Diversity Score (DDS) was also calculated and reported in the study (mean DDS = 5.03 ± 1.25); however, MDD-W was chosen as the primary outcome because of its established cutoff and public health relevance. We have acknowledged the limitations of dietary assessment and recommend that future studies consider complementary dietary indicators to provide a more comprehensive assessment of dietary quality.

Comment 11: The results indicate that 31.9% of participants did not achieve minimum dietary diversity, suggesting potential nutritional inadequacies. However, the authors do not provide a clear explanation for this finding. I would like to inquire about the potential factors contributing to this lack of dietary diversity, such as socioeconomic status, education level, or access to healthcare services. Additionally, it would be beneficial to know whether the authors collected data on the participants' dietary habits during pregnancy, as this may have implications for their dietary diversity during lactation. The results also show that the intake of nutrient-dense food groups, such as eggs, nuts and seeds, and vitamin A-rich fruits and vegetables, was substantially lower. This raises concerns about potential micronutrient gaps among lactating mothers. I would like to ask whether the authors considered assessing the micronutrient status of the participants, using biomarkers such as hemoglobin or serum retinol, to confirm the presence of micronutrient deficiencies.

Thank you for this valuable comment. The factors associated with lower dietary diversity were explored in the multivariable analysis and included lower maternal education, unemployment, lower household wealth, food insecurity, limited nutrition knowledge, and lower utilization of antenatal care services. These findings have been further emphasized in the Discussion section.The study did not collect information on dietary habits during pregnancy; therefore, the potential influence of prenatal dietary practices on dietary diversity during lactation could not be assessed. In addition, micronutrient biomarkers such as hemoglobin or serum retinol were not measured. As a result, micronutrient deficiencies could not be directly assessed, and the findings were interpreted cautiously as potential micronutrient inadequacies rather than confirmed deficiencies. We have acknowledged this limitation in the revised manuscript and recommend future studies incorporating biochemical assessments to better evaluate micronutrient status.

Comment 12: Table 3 presents the proportion of lactating mothers who consumed each of the 10 food groups within the past 24 hours. However, it is unclear whether the authors accounted for the potential seasonal variability in food availability and consumption patterns. Nepal is a country with diverse geography and climate, which may impact the availability and accessibility of certain food groups. I would like to know whether the authors collected data during a specific season or whether they accounted for seasonal variations in their analysis.

Response:
Thank you for this important observation. Data collection was conducted between October 2020 and March 2021. Seasonal variation was not explicitly assessed or controlled for in the analysis. We acknowledge that the availability and accessibility of certain foods may vary across seasons and could influence dietary diversity. To address this concern, we have expanded the Strengths and Limitations section to acknowledge seasonal variation as a potential limitation of the study and recommend that future studies incorporate repeated measurements across different seasons.

Comment 13: The authors highlight the potential risk of "hidden hunger" due to the low consumption of micronutrient-rich foods. While this is an important concern, it is essential to consider the broader context of maternal nutrition in Nepal. I would like to inquire about the authors' plans to disseminate their findings to relevant stakeholders, such as policymakers, healthcare providers, and community leaders, to inform the development of targeted nutrition interventions.
Note 1: What alternative indicators were considered for assessing dietary diversity, and why was the MDD-W indicator chosen? What factors contributed to the lack of dietary diversity among 31.9% of participants, and how did these factors impact the results? Were data collected on the participants' dietary habits during pregnancy, and if so, how did these data inform the analysis? Were biomarkers used to assess the micronutrient status of participants, and if so, what were the results? How did the authors account for seasonal variability in food availability and consumption patterns? What plans do the authors have to disseminate their findings to relevant stakeholders, and how will these findings inform the development of targeted nutrition interventions?
Note 2: The study's finding that 31.9% of participants did not achieve minimum dietary diversity raises questions about the underlying factors contributing to these nutritional inadequacies. Is the low consumption of micronutrient-rich foods, such as eggs, nuts and seeds, and vitamin A-rich fruits and vegetables, a result of economic constraints, limited access to markets, or cultural preferences? Furthermore, the study's reliance on a 24-hour dietary recall may introduce recall bias, particularly among participants with limited literacy or numeracy skills. How did the researchers account for potential biases in the data collection process, and what measures were taken to ensure the accuracy and reliability of the dietary recall data?

Thank you for this valuable comment. Factors associated with lower dietary diversity included lower maternal education, unemployment, lower household wealth, food insecurity, limited nutrition knowledge, and lower ANC utilization. Dietary habits during pregnancy, micronutrient biomarkers, and seasonal variation were not assessed and have been acknowledged as study limitations. The MDD-W indicator was selected because it is an internationally validated measure for assessing dietary diversity among women of reproductive age. We agree that dissemination of these findings is important and believe they can help inform nutrition education, food security, and maternal health interventions for lactating mothers in Nepal.

Comment 14: Comment 14: Does the MDD-W indicator adequately account for the cultural significance of starchy staples, such as rice, maize, and potatoes, which are consumed by all participants, but may contribute to a high glycemic load and potentially exacerbate micronutrient deficiencies?

Response:
Thank you for this important comment. The MDD-W indicator is designed to assess dietary diversity and serve as a proxy for micronutrient adequacy rather than evaluate glycemic load or the nutritional quality of individual foods. While starchy staples are an important component of diets in Nepal and were widely consumed among participants, the MDD-W framework emphasizes consumption across multiple food groups beyond staple foods. We acknowledge that MDD-W does not fully capture all aspects of dietary quality and recommend that future studies incorporate complementary dietary indicators to provide a more comprehensive assessment.

Comment 15: How do the study's results inform our understanding of the effectiveness of existing nutrition interventions, such as the National Nutrition Policy and the Nepal Health Sector Strategy? What policy and programmatic reforms are needed to address the micronutrient gaps identified in this study, and how can these reforms be tailored to the specific cultural, economic, and environmental contexts of semi-urban municipalities in Nepal?

Response:
Thank you for this important comment. While this study did not directly evaluate the effectiveness of existing national nutrition policies, the findings may inform policy and programmatic priorities by highlighting the need to strengthen maternal nutrition counseling, household food security, women’s education and economic participation, and access to affordable nutrient-rich foods. We have emphasized that interventions should be tailored to the semi-urban context of Nepal, where cultural practices, economic constraints, and changing food environments may jointly influence maternal dietary diversity.

Comment 16: The manuscript's conclusion that nearly one-third of the population is at risk of nutritional inadequacies due to low dietary diversity warrants further investigation into the social and economic determinants of dietary diversity. What role do factors such as women's empowerment, household income, and access to education and healthcare play in shaping dietary diversity among lactating mothers? How do these factors intersect with cultural and environmental factors, such as food availability and agricultural practices, to influence dietary diversity and nutrition outcomes?

Thank you for this valuable comment. Our findings suggest that women's empowerment, household wealth, education, food security, nutrition knowledge, and healthcare utilization are important factors associated with maternal dietary diversity. We acknowledge that these factors likely interact with broader cultural, social, and environmental influences, including food availability and local dietary practices. We have expanded the Discussion section to emphasize the complex and multifactorial nature of maternal dietary diversity and recommend further research to better understand these interrelationships in semi-urban settings.

Comment 17: The authors should provide more detailed information on the sampling frame and the representativeness of the study sample. How was the sample of 251 lactating mothers selected, and what efforts were made to ensure that the sample is representative of the broader population of lactating mothers in Tarakeswor Municipality?

Thank you for this important comment. We have expanded the Methods section to provide additional details regarding the sampling frame and participant selection process. A comprehensive sampling frame of eligible lactating mothers was developed through field enumeration conducted with the support of Female Community Health Volunteers (FCHVs) and local health facilities. Participants were then selected using proportionate stratified random sampling across all 11 wards of Tarakeswor Municipality, ensuring representation from each ward and minimizing selection bias. These details have been clarified in the revised manuscript.

Comment 18: The authors should provide a clearer justification for the specific cutoff points used to define minimum dietary diversity. How did the authors determine that the chosen cutoff points accurately capture the nuances of dietary diversity among lactating mothers?

Thank you for this important comment. The cutoff of consuming at least five out of ten food groups was based on the internationally validated Minimum Dietary Diversity for Women (MDD-W) indicator developed by FAO and FHI 360. This threshold has been shown to serve as a proxy indicator of minimum micronutrient adequacy among women of reproductive age and is widely used in global nutrition research. We have clarified this rationale in the Methods section and selected the MDD-W cutoff to ensure consistency and comparability with national and international studies.

Comment 19: The multivariable logistic regression model adjusts for several sociodemographic and maternal characteristics. Nevertheless, it is unclear whether the authors controlled for other potential confounding variables, such as access to healthcare services, exposure to nutrition counseling, or the presence of comorbidities. Did the authors consider using propensity score matching or inverse probability weighting to address potential confounding?

Response:
Thank you for this important comment. The multivariable model adjusted for the sociodemographic and maternal characteristics available in the dataset. However, we acknowledge that some potentially relevant variables, such as exposure to nutrition counseling, comorbidities, and other healthcare-related factors, were not assessed and may have contributed to residual confounding. This limitation has been acknowledged in the revised manuscript. Propensity score matching and inverse probability weighting were not applied because the primary objective of the study was to identify factors associated with dietary diversity rather than estimate causal effects.

Comment 20: The significant association between women's empowerment and dietary diversity is noteworthy. However, the authors should consider conducting a mediation analysis to elucidate the mechanisms underlying this relationship. For instance, does access to education or employment opportunities mediate the relationship between women's empowerment and dietary diversity?

Response:
Thank you for this insightful suggestion. We agree that understanding the pathways linking women’s empowerment and dietary diversity is important. However, the primary objective of this study was to identify factors associated with maternal dietary diversity rather than examine causal mechanisms. Therefore, mediation analysis was beyond the scope of the present study. We have highlighted this as an important area for future research, particularly using longitudinal study designs that are better suited to investigating potential mediating relationships among empowerment, education, employment, and dietary diversity.

Comment 21: The authors used a self-reported measure of maternal nutrition knowledge. However, this may be subject to social desirability bias or recall bias. Did the authors consider using a more objective measure of nutrition knowledge, such as a standardized questionnaire or a performance-based assessment?

Response:
Thank you for this important comment. Maternal nutrition knowledge was assessed using a structured interviewer-administered questionnaire developed for the study. We acknowledge that self-reported measures may be subject to social desirability and recall bias. This limitation has been acknowledged in the revised manuscript. Future studies may benefit from using standardized and validated nutrition knowledge assessment tools to provide a more objective evaluation.

Comment 22: “Understanding dietary diversity among lactating mothers in these contexts is vital for designing equitable nutrition programs” Add the following reference:
2025. Culinary chronicles of ancient Ebla: A multidisciplinary exploration of diet, nutrition, and health in a 3rd millennium BCE Syrian civilization. Journal of Anthropological Archaeology 78, 101689.

Response:
Thank you for the suggested reference. We reviewed the recommended article; however, we did not incorporate it because it is not directly related to maternal nutrition, dietary diversity, or contemporary public health nutrition in low- and middle-income countries. We retained references that are more closely aligned with the objectives and context of the present study.

Comment 23: The association between attending four or more antenatal care visits and better dietary diversity is intriguing. However, the authors should explore the potential mechanisms underlying this relationship, such as the provision of nutrition counseling or the distribution of food supplements during ANC visits.

Response:
Thank you for this valuable comment. We agree that the relationship between antenatal care utilization and dietary diversity may operate through several mechanisms, including exposure to nutrition counseling, health education, and other maternal health services received during ANC visits. We have expanded the Discussion section to acknowledge these potential pathways while noting that the present study was not designed to directly assess the underlying mechanisms of this association.

Comment 24: The strong association between food security and dietary diversity is not surprising. Nevertheless, the authors should consider examining the relationship between food security and dietary diversity in more detail, including the potential impact of food insecurity on dietary quality and nutrient intake.

Response:
Thank you for this valuable comment. We agree that food security and dietary diversity are closely related and that food insecurity may influence both dietary quality and nutrient intake. In the present study, household food security was identified as a significant predictor of minimum dietary diversity. However, detailed assessment of nutrient intake and dietary quality was beyond the scope of the study. We have expanded the Discussion section to further highlight the importance of food security as a key determinant of maternal dietary diversity and recommend additional research to explore these relationships in greater detail.

Comment 25: The utilization of multivariable logistic regression to identify the correlates of minimum dietary diversity is commendable, but the modelling approach may have been enhanced by incorporating interaction terms to account for potential synergies between sociodemographic and maternal characteristics. For instance, the interplay between employment status and household socioeconomic position may have yield more nuanced insights into the determinants of dietary diversity.
Note 3: The study's reliance on self-reported data for variables such as food security and maternal nutrition knowledge may introduce biases, as respondents' perceptions of these constructs may not align with objective measures. Therefore, it is essential to consider the development of more robust instruments for assessing these variables, such as the use of standardized questionnaires or observational methods. Moreover, the finding that women's empowerment is a significant predictor of dietary diversity raises questions about the underlying mechanisms driving this relationship. Is it the case that empowered women are more likely to prioritize their own nutritional needs, or do they possess greater autonomy to make decisions about food choices within their households?
Note 4: The generalizability of the findings to other contexts, particularly in urban or rural areas with distinct sociocultural and economic profiles, is uncertain. Consequently, it is crucial to replicate this study in diverse settings to substantiate the results and identify potential variations in the factors associated with dietary diversity. Ultimately, a more comprehensive understanding of the complex interplay between maternal characteristics, household factors, and dietary diversity is essential for devising effective strategies to promote optimal nutrition among lactating mothers in Nepal and beyond.
Note 5: How can healthcare providers and policymakers leverage the association between women's empowerment and dietary diversity to inform the development of interventions aimed at promoting maternal nutrition? What role can community-based initiatives, such as nutrition education programs or support groups, play in enhancing dietary diversity among lactating mothers? By exploring these questions, the study could have provided a more concrete foundation for translating its findings into actionable recommendations for improving maternal health outcomes.

Response:
Thank you for these valuable suggestions. The primary objective of this study was to identify factors associated with minimum dietary diversity rather than examine interaction effects or causal pathways. Therefore, interaction terms were not included in the final model. We acknowledge that relationships among employment, socioeconomic position, empowerment, and dietary diversity may be complex and warrant further investigation in future studies.

We also recognize that self-reported measures of food security, nutrition knowledge, and empowerment may be subject to reporting bias, and this limitation has been acknowledged in the revised manuscript. The mechanisms linking women’s empowerment and dietary diversity were not directly assessed; however, greater decision-making autonomy may influence access to and choices regarding nutritious foods. Finally, we agree that the generalizability of the findings may be limited and have emphasized the need for replication in diverse settings. The Discussion and Conclusion sections have been strengthened to highlight the potential role of nutrition education, community-based programs, women’s empowerment initiatives, and multi-sectoral interventions in improving maternal dietary diversity.

Comment 26: The mean DDS was reported as 5.03 (±1.25), with 68% of mothers meeting the MDD-W threshold. However, the process of calculating the DDS and the specific food groups considered under the MDD-W framework are not adequately described. Could the authors provide a detailed explanation of their methodology, including how they handled potential variations in food group classifications and portion sizes?

Response:
Thank you for this comment. We revised the Methods section to provide a clearer description of how DDS and MDD-W were calculated. Foods reported in the 24-hour dietary recall were classified into the 10 FAO-defined MDD-W food groups, and the DDS was calculated as the total number of food groups consumed. Women consuming foods from five or more groups were classified as achieving MDD-W. Because MDD-W is based on food group consumption rather than portion size, portion sizes were not used in determining whether a food group was counted.

Comment 27: The study found that maternal education, employment status, household wealth, and food security were significant predictors of dietary diversity. Yet, the mechanisms through which these factors influence dietary choices are complex and multifaceted. How do the authors suggest policymakers and practitioners address these socioeconomic determinants to improve maternal dietary diversity, especially in semi-urban settings where access to resources can be limited and uneven?

Response:
Thank you for this valuable comment. Our findings suggest that improving maternal dietary diversity requires addressing broader socioeconomic determinants, including education, employment opportunities, household food security, and economic well-being. We recommend strengthening nutrition education and counseling, supporting women’s economic participation, expanding food security and social protection programs, and improving access to affordable nutrient-rich foods. These approaches may be particularly important in semi-urban settings where resources and services are often unevenly distributed. We have further emphasized these implications in the Discussion and Conclusion sections.

Comment 28: What specific nutrition education strategies do the authors recommend integrating into antenatal and postnatal care services to enhance maternal dietary diversity, and how can these strategies be tailored to the semi-urban context of Nepal?

Response:
Thank you for this important comment. We recommend integrating practical nutrition education into antenatal and postnatal care services, including counseling on the importance of dietary diversity, locally available nutrient-rich foods, appropriate maternal nutrition during lactation, and strategies for improving household food choices within existing resource constraints. In semi-urban settings such as Tarakeswor Municipality, these interventions could be delivered through routine maternal health services, Female Community Health Volunteers (FCHVs), community awareness programs, and culturally appropriate educational materials tailored to local dietary practices and food availability.

Comment 29: How do the authors account for the potential role of cultural beliefs and practices in shaping maternal dietary diversity in semi-urban Nepal, and what implications does this have for the design of culturally sensitive nutrition interventions?

Response:
Thank you for this important comment. We acknowledge that cultural beliefs, food preferences, and dietary practices may influence maternal dietary diversity in semi-urban Nepal. Although these factors were not directly assessed in the present study, they may affect food choices and consumption patterns during lactation. We have highlighted in the Discussion and Limitations sections that future research should further explore cultural influences on maternal diets. Nutrition interventions should be culturally sensitive, community-based, and tailored to local dietary practices and food environments to improve their effectiveness and acceptability.

Comment 30: The study highlights the need for multi-sectoral strategies that address both structural and behavioral barriers to improving maternal dietary diversity. What specific policy recommendations do the authors have for the government of Nepal and other low- and middle-income countries facing similar challenges, particularly in terms of integrating nutrition-sensitive interventions with social protection programs and livelihood support for women?

Response:
Thank you for this valuable comment. Based on our findings, we recommend strengthening nutrition-sensitive interventions through integration with social protection programs, food security initiatives, women's education, and livelihood support schemes. Policymakers should prioritize improving access to affordable nutrient-rich foods, expanding nutrition education through maternal health services, and promoting women's economic participation and decision-making autonomy. Multi-sectoral collaboration among health, agriculture, education, and social welfare sectors may help address the structural and behavioral factors influencing maternal dietary diversity in Nepal and similar low- and middle-income country settings.

Comment 31: “Similarly, employment status was positively associated with dietary diversity” Add the following reference:
2024. A cross-sectional study on demographic characteristics, nutritional knowledge, and supplement use patterns. International Journal of Kinesiology & Sports Science 12 (4), 21-30.

Response:
Thank you for the suggested reference. We reviewed the recommended article; however, we retained references that were more directly related to maternal nutrition, dietary diversity, and the socioeconomic determinants of dietary practices among women of reproductive age. These references were considered more closely aligned with the objectives and context of the present study.

Comment 32: How do the intersecting dynamics of urbanization, poverty, and gender norms influence maternal dietary practices, and what are the implications for the design of effective nutrition interventions?

Response:
Thank you for this insightful comment. We acknowledge that urbanization, poverty, and gender norms interact in complex ways to influence maternal dietary practices. While urbanization may improve access to food markets and health services, economic constraints and gender-related barriers may limit women’s ability to access and utilize these resources. These findings support the need for nutrition interventions that address both structural factors, such as food security and economic opportunities, and behavioral factors, including nutrition knowledge and women’s empowerment. We have further emphasized these considerations in the Discussion section.

Comment 33: What are the most effective strategies for delivering nutrition education and counseling to lactating mothers, and how can these interventions be integrated into existing healthcare systems and community-based programs? Additionally, how can policymakers and practitioners balance the need for culturally sensitive and context-specific interventions with the need for scalable and sustainable solutions that can be replicated across diverse settings?

Response:
Thank you for this valuable comment. We recommend integrating nutrition education and counseling into routine antenatal, postnatal, and community health services using approaches that are practical, culturally appropriate, and locally relevant. Female Community Health Volunteers (FCHVs), healthcare providers, and community-based programs can play important roles in delivering consistent nutrition messages and promoting healthy dietary practices among lactating mothers. To ensure both effectiveness and sustainability, interventions should be adapted to local cultural and dietary contexts while maintaining core evidence-based nutrition principles that can be scaled and replicated across similar settings.

#Reviewer 5:

Abstract:
The abstract represents adjusted odds ratios from Table 4, as it appears internally inconsistent. Check Table 4 and rerun calculations and then write abstract again

Response:

Thank you for this careful observation. Following your comment, we rechecked Table 4, the original regression outputs, and the Abstract. During this review, we identified errors in the transcription and presentation of some frequencies and percentages in Table 4. However, the crude odds ratios, adjusted odds ratios, confidence intervals, and statistical significance values reported in the manuscript were generated from the original dataset and remained unchanged.

Table 4 has been revised to correct the frequency presentation and ensure consistency with the study sample and variable distributions. We also reviewed and revised the Abstract to ensure that all reported adjusted odds ratios and confidence intervals accurately reflect the final multivariable regression model.

Comment:
The operational definition of semi-urban should be based on Nepalese administrative classification or a recognized urbanization framework, not a general website. The paragraph on semi-urban food environments is useful but should be linked more directly to the study municipality and available local data. A study area map can be more useful to visualize the study area, and the research gap should be more clarified.

Response:

Thank you for this constructive comment. We revised the Introduction to provide a clearer description of semi-urban municipalities within Nepal's administrative and urbanization context and strengthened the discussion of semi-urban food environments. We also linked these concepts more directly to Tarakeswor Municipality by highlighting its transitional characteristics, including mixed livelihoods, expanding market access, and continued reliance on traditional household food practices (Lines 76–91). Furthermore, we clarified the research gap by emphasizing the limited evidence available on maternal dietary diversity among lactating mothers in Nepal's semi-urban settings and the need for context-specific evidence from transitional municipalities (Lines 103–111). In addition, a study area map has been added as Figure 1 in the Methods section to improve visualization of the study location and provide greater geographic context for the study setting.

Comment:
In line 145, although 700 eligible lactating mothers were identified across 11 wards, the finite population correction formula uses N = 2500. This is not explained. There are also calculation errors related to adjusted n. The authors must clarify the true sampling frame, redo the calculation, and revise the sampling section accordingly. In addition, line 128 states that 251 mothers were selected, but Table 1 states that 263 were selected and 251 completed interviews.

Response:
Thank you for this important observation. We revised the Sample Size and Sampling Method section to clarify the distinction between the estimated source population and the final sampling frame. The finite population correction was applied using the estimated source population available from municipal records during the study planning phase (N = 2500). Subsequently, field enumeration conducted with the support of Female Community Health Volunteers and local health facilities identified 700 eligible lactating mothers, which constituted the final sampling frame for participant selection.We also corrected the participant flow to avoid confusion. The manuscript now clearly states that 263 mothers were initially selected using proportionate stratified random sampling, and 251 completed the interviews and were included in the final analysis. The sample size calculation, Table 1, and the sampling description have been revised for consistency.

Comment:
The study period and data collection period are not matched and use a specific date format.

Response:

Thank you for identifying this inconsistency. We reviewed and revised the Study Design and Setting section (Lines 115–117) to ensure consistency between the overall study period and the participant recruitment/data collection period. The manuscript now clearly states that the study was conducted between October 2020 and March 2021, while participant recruitment and data collection took place from 9 October 2020 to 24 March 2021. We also standardized the date format throughout the manuscript to improve clarity and consistency.

Comment:
MDD-W food groups are incorrectly described in the Methods. Although Table 3 uses the correct-looking groups, in Line 169, "oils and fats" is included. Please check the FAO standard MDD-W 10 food groups.

Response:

Thank you for identifying this error. We reviewed the description of the MDD-W indicator and revised the Methods section (Lines 181–186) to ensure consistency with the FAO Minimum Dietary Diversity for Women (MDD-W) guidelines. The incorrect reference to "oils and fats" has been removed, and the ten standard MDD-W food groups are now presented using the correct FAO classification. Table 3 and the corresponding text have been checked for consistency throughout the manuscript.

Comment:
The research can add more independent variables such as premarital conditions, number of children, age of giving birth, number of family members, etc. Education, age, wealth, food security, and empowerment categories should be described exactly as used in regression.

Response:

Thank you for this valuable suggestion. We agree that additional variables such as age at first birth, household size, parity, and other reproductive or household characteristics may influence maternal dietary diversity. However, the analysis was limited to variables available in the study dataset and those considered most relevant based on existing literature and study objectives. We have acknowledged this as a limitation and recommend that future studies include a broader range of sociodemographic and reproductive factors.

In addition, we revised the Methods section and Table 4 to clearly describe the categorization and coding of education, age, wealth status, food security, and women’s empowerment variables exactly as used in the regression analysis. These revisions improve the transparency and reproducibility of the analytical approach.

Comment:
Table 4 appears mathematically impossible. The final sample size is n = 251, and Table 2 reports dietary diversity as 171 diverse and 80 not diverse. Several frequencies reported in Table 4 exceed the study sample size or are inconsistent with Table 2. There is also inconsistent reporting of variable coding. Before providing regression findings, the authors should clearly explain all recoding and collapsing choices.

Response:

Thank you for this careful observation. We thoroughly reviewed Table 4 and identified errors in the transcription and presentation of some frequencies and percentages during table preparation. These errors have been corrected to ensure consistency with the study sample size (n = 251) and the distributions reported in Table 2. Importantly, the crude odds ratios, adjusted odds ratios, confidence intervals, and statistical significance values were generated from the original dataset and remained unchanged.

To improve transparency, we added a new subsection entitled “Variable Coding for Regression Analysis” (Lines 247–254). This section explicitly describes the recategorization and collapsing of variables used in the regression analyses, including age, educational attainment, wealth status, food security, and women’s empowerment. These categorizations were applied consistently throughout the logistic regression analyses and are now aligned with the categories presented in Table 4.

Comment:
The number of predictors compared to the number of outcome events may cause the regression model to be overfitted. The authors should describe the precise variables included in the model, provide justification for the model definition, and consider a more economical model.

Response:

Thank you for this important comment. We have revised the Statistical Analysis section to clearly describe the variables included in the multivariable logistic regression model and the rationale for their selection. Variables were selected based on prior literature, biological plausibility, and their relevance to maternal dietary diversity. To improve transparency, we also clarified the coding and categorization of variables used in the regression analysis. While we acknowledge the potential risk of overfitting associated with a moderate sample size, the number of predictors included was considered appropriate relative to the number of outcome events and the study objectives. This limitation has been acknowledged in the revised manuscript.

Comment:
Inconsistent interpretation of statistical significance. The ANC result should be reported with aOR, CI, and additionally an exact p-value to support interpretation of statistical significance.

Response:

Thank you for this helpful suggestion. We have revised the Results section (Lines 353–355) to report the association between antenatal care attendance and minimum dietary diversity using the adjusted odds ratio, 95% confidence interval, and exact p-value. Specifically, mothers attending four or more ANC visits had higher odds of achieving minimum dietary diversity (aOR = 1.9; 95% CI: 1.0–3.4; p = 0.048). This revision improves the transparency and interpretation of the statistical findings.

Conclusion:
Even though education and nutrition knowledge have larger reported adjusted odds ratios than food security, you stated food insecurity was the strongest barrier. So, careful justification is needed.

Response:

Thank you for this important observation. We agree that the original wording could imply that food insecurity was the strongest determinant of dietary diversity. We have revised the Conclusion to use more balanced language and to emphasize that food insecurity is one of several important factors associated with maternal dietary diversity, alongside education, employment status, household wealth, nutrition knowledge, and women’s empowerment. The revised conclusion now reflects the overall pattern of findings rather than emphasizing a single determinant.

Comment:
Careful checking of the reference list is necessary. A number of references, such as the citation for Bennett's Law and URLs with tracking artifacts, are weak, inconsistent, or incomplete.

Response:

Thank you for this valuable observation. We carefully reviewed and revised the reference list to improve its accuracy, consistency, and completeness. Several references were updated to include full bibliographic information, incomplete citations were corrected, and formatting was standardized according to journal guidelines. Non-scholarly or weak sources were replaced with more authoritative references where appropriate, including replacing the previous website-based citation used to describe semi-urban settings with official Nepal Census sources. We also removed irrelevant references, corrected citation details, and reviewed all URLs to eliminate inconsistencies and ensure accessibility. These revisions have strengthened the quality and reliability of the reference list.
