# Supplementary material for: Maternal dietary diversity and its correlates in a semi-urban municipality of Nepal: A cross-sectional study
Source: PLOS Glob Public Health. 2026 Aug 25;6(8):e0005711. doi: 10.1371/journal.pgph.0005711 (PMC13505919; doi:10.1371/journal.pgph.0005711)
Supplement: Supplementary file 8 [file pgph.0005711.s010.docx]

Response to reviewers

Comment 34: The manuscript provides a critical foundation for understanding the complex relationships between dietary diversity, nutrition, and health among lactating mothers in Nepal. However, to fully capture the nuances of these relationships and to inform effective policy and programmatic responses, further research is needed to address the methodological, theoretical, and contextual limitations of the study. By doing so, researchers and policymakers can work together to develop more effective and sustainable solutions to address the pressing issue of "hidden hunger" and promote optimal nutrition and health outcomes among lactating mothers and their children.

We sincerely thank the reviewer for this valuable suggestion. We agree that additional research is needed to further understand the complex relationships among dietary diversity, micronutrient adequacy (including hidden hunger), and maternal and child health outcomes. Accordingly, we have strengthened the **Conclusion** by explicitly recommending future longitudinal, intervention, and implementation research to address these issues and evaluate the effectiveness of nutrition interventions across diverse settings in Nepal. We have also emphasized the contribution of the current study as baseline evidence to inform future research and public health policy. **These revisions have been incorporated in the Conclusion (Lines 469–472).**

Comment 35: The study's findings may not be generalizable to other contexts, particularly in urban or rural areas with distinct sociocultural and economic profiles. The authors should discuss the potential limitations of their study in terms of external validity and the need for further research to replicate their findings in diverse settings.

We sincerely thank the reviewer for this important comment. We agree that the findings should be interpreted in light of the study's contextual setting. Accordingly, we have strengthened the Strengths and Limitations section by explicitly acknowledging that the study was conducted in a single semi-urban municipality and that the findings may not be generalizable to other urban, rural, or ecologically and culturally diverse settings in Nepal. We have also emphasized the need for future studies to replicate and validate these findings in different geographic, socioeconomic, and cultural contexts. These revisions have been incorporated in the Strengths and Limitations section (Lines 500–514) and the Conclusion (Lines 469–472).

Comment 36: The study employed a cross-sectional design, which, while useful for establishing associations, does not allow for the determination of causality. How do the authors plan to address the potential limitations of their study design in terms of implying causal relationships between the observed correlates and maternal dietary diversity?

We sincerely thank the reviewer for this important comment. We agree that the cross-sectional design of our study does not permit causal inference and allows only the identification of associations between maternal dietary diversity and the observed correlates. Accordingly, we have strengthened the Strengths and Limitations section by explicitly stating that the cross-sectional design precludes establishing temporal or causal relationships. Throughout the manuscript, we have also carefully used associative language (e.g., "associated with" rather than "caused by") when interpreting our findings. Furthermore, we have recommended future longitudinal and intervention studies to better evaluate causal relationships between maternal dietary diversity and its determinants. These revisions have been incorporated in the Strengths and Limitations section (Lines 488–490) and the Conclusion (Lines 469–472).

Comment 37: Given the cross-sectional nature of the study, longitudinal research designs could provide valuable insights into how maternal dietary diversity changes over time and in response to interventions. What directions do the authors propose for investigating the dynamics of maternal dietary diversity in semi-urban settings, and how can such research contribute to the development of effective, sustainable nutrition programs?

We sincerely thank the reviewer for this valuable suggestion. We agree that longitudinal research is important for understanding changes in maternal dietary diversity over time and for evaluating the effectiveness of nutrition interventions. This has been addressed in the revised Conclusion (Lines 469–472), where we recommend future longitudinal, intervention, and implementation studies to further investigate maternal dietary diversity, micronutrient adequacy (including hidden hunger), and maternal and child health outcomes, as well as to evaluate the effectiveness of nutrition interventions across diverse settings in Nepal. We also note that such research could provide stronger evidence to inform the development of effective and sustainable maternal nutrition programs and policies.

Comment 38: The study's findings also highlight the need for more research on the relationships between dietary diversity, micronutrient intake, and maternal and child health outcomes in semi-urban settings. What are the potential consequences of "hidden hunger" and micronutrient deficiencies for maternal and child health, and how can nutrition interventions be designed to address these needs? Moreover, how can the study's findings be used to inform the development of national nutrition policies and programs, such as Nepal's Multi-Sector Nutrition Plan, and what are the implications for the design of targeted interventions in comparable settings undergoing rapid urban transitions?

We sincerely thank the reviewer for this thoughtful and constructive comment. We agree that understanding the relationships between dietary diversity, micronutrient adequacy (including hidden hunger), and maternal and child health outcomes is essential for developing effective nutrition interventions and informing public health policies. Accordingly, we have strengthened the Discussion by emphasizing the implications of our findings for evidence-based policy and practice. Specifically, we highlight that the findings can inform Nepal's Multi-Sector Nutrition Plan by supporting context-specific, multi-sectoral interventions that integrate maternal nutrition education, improved access to affordable nutrient-rich foods, social protection, and women's economic empowerment to reduce hidden hunger and improve maternal and child health outcomes in rapidly urbanizing settings. We also recommend future longitudinal, intervention, and implementation research to further investigate these relationships and evaluate the effectiveness of nutrition interventions. These revisions have been incorporated in the Discussion (Lines 451–456) and Conclusion (Lines 469–476).

Comment 39: The study relies on a cross-sectional design, which may not be suitable for establishing causal relationships between dietary diversity and its determinants. The authors should justify the choice of study design and discuss the potential limitations of using a cross-sectional approach.

We sincerely thank the reviewer for this important comment. We agree that a cross-sectional design does not permit causal inference. The study employed a community-based cross-sectional design because it was appropriate for estimating the prevalence of minimum dietary diversity and identifying factors associated with dietary diversity among lactating mothers. We have clarified this rationale in the Strengths and Limitations section and have explicitly acknowledged that the cross-sectional design precludes establishing causal or temporal relationships.

Comment 40: The study uses the Minimum Dietary Diversity for Women (MDD-W) indicator, which is defined as the consumption of at least five out of ten specified food groups in the previous 24 hours. However, the authors do not provide sufficient information on how the food groups were defined and measured. For instance, how were the food groups classified, and what specific foods were included in each group?

We sincerely thank the reviewer for this valuable comment. The requested information is already provided in the Dietary Diversity subsection of the Methods. As described, dietary diversity was assessed using the FAO Minimum Dietary Diversity for Women (MDD-W) indicator based on a 24-hour dietary recall. The manuscript specifies that foods were classified into the ten MDD-W food groups (grains, white roots and tubers and plantains; pulses; nuts and seeds; dairy products; meat, poultry and fish; eggs; dark green leafy vegetables; other vitamin A-rich fruits and vegetables; other vegetables; and other fruits), and women consuming foods from five or more of these food groups were classified as achieving minimum dietary diversity. Please see the Dietary Diversity subsection of the Methods (Lines 179–188).

Comment 42: The study highlights the importance of combining nutrition education with broader social and economic support to improve maternal dietary practices. However, the authors do not provide sufficient evidence to support this claim. What specific nutrition education programs and social and economic support interventions have been shown to be effective in improving dietary diversity among lactating mothers in semi-urban settings?

We sincerely thank the reviewer for this valuable comment. We agree that providing examples of effective nutrition education and social and economic support interventions strengthens the interpretation of our findings**. Accordingly, we have expanded the Discussion** by including examples of evidence-based interventions reported in low- and middle-income countries, including individualized and group-based nutrition counseling during antenatal and postnatal care, community-based behavior change communication delivered through community health workers, women's support groups, conditional cash transfer and social protection programs, livelihood and income-generation initiatives, home gardening, and food supplementation or food voucher programs. These interventions have been associated with improved dietary diversity and maternal nutritional outcomes and support our recommendation for integrated, multi-sectoral approaches to improving maternal nutrition. **These revisions have been incorporated in the Discussion (Lines 452–458).**

Comment 43: The study notes that household food insecurity is a primary barrier to dietary diversity, but the authors do not provide sufficient information on how food insecurity was measured. What specific indicators were used to assess food insecurity, and how were these indicators validated?

We sincerely thank the reviewer for this valuable comment. The requested information is already provided in the Household Food Insecurity Access Scale (HFIAS) subsection of the Methods. Household food insecurity was assessed using the validated Household Food Insecurity Access Scale (HFIAS), a standardized instrument developed by the Food and Nutrition Technical Assistance (FANTA) Project. The manuscript describes the nine structured questions assessing uncertainty about food supply, insufficient food quality, and inadequate food intake over the previous 30 days, the standardized scoring procedure (0–27), and the categorization of households according to the HFIAS guidelines. For the regression analysis, households were further dichotomized into food-secure and food-insecure groups. Please see the HFIAS subsection of the Method.

Comment 44: The study also highlights the importance of understanding dietary diversity among lactating mothers in semi-urban settings, but the authors do not provide sufficient context on the specific semi-urban municipality of Nepal that was studied. What are the unique characteristics of this municipality, and how do these characteristics impact dietary diversity among lactating mothers?

Response to Comment 44:

We sincerely thank the reviewer for this valuable comment. We agree that providing additional context regarding the study setting strengthens the interpretation of our findings. Accordingly, we have expanded the Discussion to further describe the unique characteristics of Tarakeswor Municipality as a rapidly urbanizing, semi-urban setting where improved physical access to markets coexists with persistent socioeconomic constraints, traditional dietary practices, and unequal access to nutritious foods. We also clarify how these contextual characteristics create a hybrid food environment that may influence dietary diversity among lactating mothers. These revisions provide additional context for interpreting our findings and their relevance to other rapidly urbanizing settings in Nepal and similar low- and middle-income countries.

Comment 45: The study aims to inform locally tailored, equity-driven interventions to promote maternal dietary diversity and improve health outcomes in rapidly urbanizing LMIC settings. However, the authors do not provide sufficient information on how the study's findings can be translated into practice. What specific policy and programmatic recommendations can be made based on the study's findings, and how can these recommendations be implemented in a way that is culturally and contextually relevant?

Comment 45: The study aims to inform locally tailored, equity-driven interventions to promote maternal dietary diversity and improve health outcomes in rapidly urbanizing LMIC settings. However, the authors do not provide sufficient information on how the study's findings can be translated into practice. What specific policy and programmatic recommendations can be made based on the study's findings, and how can these recommendations be implemented in a way that is culturally and contextually relevant?

**Response to Comment 45:**
We sincerely thank the reviewer for this insightful comment. We agree that translating the study findings into practical, culturally appropriate interventions is essential for improving maternal nutrition. **Accordingly, we have strengthened the Discussion** by providing more specific policy and programmatic recommendations. In particular, we emphasize that context-specific strategies should integrate maternal nutrition education, improved access to affordable nutrient-rich foods, social protection, and women's economic empowerment. We further clarify that these interventions should be implemented through existing community health systems, including Female Community Health Volunteers (FCHVs), while incorporating locally available foods, culturally appropriate nutrition education, and community engagement to ensure that interventions are contextually relevant, acceptable, and sustainable. These revisions strengthen the practical implications of our findings for Nepal and other rapidly urbanizing low- and middle-income country settings.

Comment 46: What would be the justification of using the MDD-W indicator in this context, and how the results of this study can be compared with the other studies which have used different indicators? How the authors can rule out the possibility that the observed associations between dietary diversity and its determinants are due to residual confounding or reverse causality? What would be the potential generalizability of the study findings to other semi-urban settings in Nepal and other LMICs, and how the authors can justify the generalizability of the results? How the study's findings can be used to inform policy and programmatic decisions to improve maternal dietary diversity in semi-urban settings, and what specific recommendations can be made based on the study's results?

We sincerely thank the reviewer for this comprehensive comment. The issues raised have been addressed throughout the revised manuscript. Specifically, we have clarified the justification for using the validated FAO MDD-W indicator, acknowledged the limitations of the cross-sectional design and potential residual confounding in the Strengths and Limitations section, discussed the generalizability of the findings to comparable semi-urban settings, and strengthened the Discussion and Conclusion by providing policy and programmatic recommendations, including culturally appropriate, community-based interventions. We believe these revisions adequately address the reviewer's concerns.

Comment 47: The authors should provide more context on the specific semi-urban municipality of Nepal that was studied, and discuss the potential generalizability of the study findings to other settings. The authors should also provide more information on the potential implications of the study's findings for policy and programmatic decisions, and discuss the potential avenues on maternal dietary diversity in semi-urban settings. So, you must perform (1) Longitudinal studies to examine the causal relationships between dietary diversity and its determinants, (2) Intervention studies to evaluate the effectiveness of specific nutrition education programs and social and economic support interventions in improving dietary diversity among lactating mothers, (3) Qualitative studies to explore the cultural and contextual factors that influence dietary diversity among lactating mothers in semi-urban settings, and (4) Studies to examine the potential generalizability of the study findings to other semi-urban settings in Nepal and other LMICs.

We sincerely thank the reviewer for this valuable and comprehensive comment. The issues raised have been addressed throughout the revised manuscript. Specifically, we have provided additional context on the characteristics of the semi-urban study setting and discussed the potential generalizability of our findings to comparable semi-urban settings in Nepal and other low- and middle-income countries. We have also strengthened the Discussion and Conclusion by expanding the policy and programmatic implications of our findings and by recommending future longitudinal, intervention, qualitative, and multisite studies to further investigate maternal dietary diversity, evaluate context-specific interventions, explore sociocultural influences, and assess the generalizability of our findings.

Comment 48: The study's reliance on a cross-sectional design limitations its ability to establish causality between the variables under investigation. The authors' conclusions about the relationships between dietary diversity and various sociodemographic, economic, and household-level factors are thus based on correlations rather than causal links. For instance, the authors suggest that household food insecurity is a primary barrier to dietary diversity, but they do not provide longitudinal data to demonstrate how changes in food security status affect dietary diversity over time. Furthermore, the study's use of the MDD-W indicator as the primary outcome measure may not capture the complexity of dietary patterns among lactating mothers. The MDD-W indicator is based on the consumption of at least five out of ten specified food groups in the previous 24 hours, but it does not account for the quality or quantity of food consumed within each group. This oversimplification may lead to misleading conclusions about the adequacy of maternal diets. For example, a mother who consumes a diet rich in starchy staples but limited in animal-source foods, legumes, fruits, and vegetables may meet the MDD-W threshold but still be at risk of micronutrient deficiencies. The authors also propose that targeted nutrition interventions, such as behavior change communication, nutrition education, and women's empowerment programs, can improve dietary diversity among lactating mothers. However, the evidence for these interventions is largely based on studies conducted in rural or urban areas, and it is unclear whether these interventions would be effective in semi-urban settings. The unique socioeconomic and environmental characteristics of semi-urban areas, including mixed livelihoods, evolving food systems, and varying access to services, may require tailored interventions that address the specific needs and constraints of these populations.

Response to Comment 48:

We sincerely thank the reviewer for this thoughtful and comprehensive comment. We acknowledge the inherent limitations of the cross-sectional study design and have explicitly discussed the inability to infer causality, the potential for residual confounding, and the need for future longitudinal research in the Strengths and Limitations section. We have also discussed the strengths and limitations of the FAO Minimum Dietary Diversity for Women (MDD-W) indicator, including its role as a validated proxy for micronutrient adequacy and its inability to assess the quantity or quality of foods consumed. Furthermore, we have strengthened the Discussion and Conclusion by emphasizing the unique characteristics of semi-urban settings and expanding the policy and programmatic implications of our findings, including recommendations for context-specific, culturally appropriate interventions and future intervention studies. We believe these revisions adequately address the reviewer's concerns.

Comment 49: The study's sample size and selection criteria are not clearly justified. The authors do not provide information on how the sample size was determined or how participants were selected, which raises concerns about the representativeness of the sample and the potential for selection bias. Furthermore, the study's focus on lactating mothers in a single semi-urban municipality limits the generalizability of the findings to other populations and settings.

We sincerely thank the reviewer for this valuable comment. The issues raised have been addressed in the revised manuscript. The Methods section provides a detailed description of the sample size determination, including the sample size calculation, finite population correction, non-response adjustment, and the proportionate stratified random sampling procedure used to minimize selection bias and improve representativeness. We have also acknowledged in the Strengths and Limitations section that the study was conducted in a single semi-urban municipality, and therefore the findings should be generalized with caution to other settings. We believe these descriptions adequately address the reviewer's concerns.

Comment 50: The authors' conclusion that the study's findings can inform locally tailored, equity-driven interventions to promote maternal dietary diversity and improve health outcomes in rapidly urbanizing LMIC settings is overly optimistic. While the study provides some insights into the dietary diversity and correlates among lactating mothers in a semi-urban municipality of Nepal, the methodological limitations and lack of generalizability of the findings undermine the study's potential to inform policy and practice. Further research is needed to address the complex and context-specific challenges of promoting dietary diversity and improving maternal and child health in semi-urban settings.

We sincerely thank the reviewer for this thoughtful comment. We acknowledge the limitations of the cross-sectional design and the single-site setting, which may limit causal inference and generalizability. Accordingly, we have revised the Discussion, Strengths and Limitations, and Conclusion to present our findings more cautiously, emphasizing that they provide context-specific evidence to inform locally tailored interventions while highlighting the need for further longitudinal, intervention, and multisite studies before broader policy and programmatic recommendations can be made. We believe these revisions adequately address the reviewer's concerns.

Comment 51: The study's sample size calculation appears to be based on an estimated prevalence of minimum dietary diversity (45%) from a similar study done in a periurban area of Nepal. However, the authors do not provide sufficient justification for using this specific prevalence estimate, which may not accurately represent the study population. This raises questions about the generalizability of the findings. Furthermore, the study uses a 24-hour dietary recall method to assess dietary diversity, which may be subject to recall bias. While the authors attempt to minimize this bias by using probing questions and locally familiar food examples, the reliability of this method is still uncertain. It would be beneficial to explore alternative methods, such as food diaries or weighed food records, to validate the findings.

We sincerely thank the reviewer for this valuable comment. The Methods section has been clarified to justify the use of the estimated prevalence from a comparable peri-urban Nepali study for sample size calculation, as no prior data were available for the study area. We have also acknowledged the potential for recall bias associated with the 24-hour dietary recall method in the Strengths and Limitations section, while noting that trained interviewers, probing questions, and locally familiar food examples were used to minimize this bias. Furthermore, we have recommended that future studies consider complementary dietary assessment methods, such as repeated dietary recalls, food diaries, or weighed food records, to further validate dietary intake and diversity. We believe these revisions adequately address the reviewer's concerns.

Comment 52: The study also categorizes women's empowerment as a binary variable, which may oversimplify the complex and multifaceted nature of empowerment. The decision to dichotomize this variable may lead to loss of information and potential misclassification of participants. A more nuanced approach, such as using a continuous or ordinal scale, may provide a more accurate representation of women's empowerment.

**Response to Comment 52:**
We sincerely thank the reviewer for this valuable comment. We acknowledge that women's empowerment is a multidimensional construct and that dichotomizing the variable may not fully capture its complexity. In this study, the categorization was based on the predefined scoring approach of the validated measurement tool and was used to facilitate interpretation and statistical analysis. We have acknowledged this as a limitation in the manuscript and recommend that future studies consider using continuous or multidimensional measures of women's empowerment to better capture its complexity. We believe this adequately addresses the reviewer's concern.

Comment 53: The study controls for various sociodemographic variables, including wealth quintiles, which are collapsed into three categories. However, the authors do not provide a clear justification for this categorization, which may mask important differences between the wealthiest and poorest quintiles. It is essential to explore alternative categorizations or use continuous variables to capture the full range of wealth disparities.

We sincerely thank the reviewer for this valuable comment. The wealth quintiles were collapsed into three categories to ensure adequate sample sizes within each group and to improve the stability and interpretability of the regression analysis. We acknowledge that this approach may reduce the ability to detect differences across individual wealth quintiles and have noted this as a limitation in the manuscript. We recommend that future studies with larger sample sizes consider retaining all wealth quintiles or using alternative measures of socioeconomic status to better capture wealth disparities.

Comment 54: The study's regression analysis reveals several significant associations between independent variables and dietary diversity. However, the authors do not provide sufficient discussion on the potential mediating or moderating factors that may influence these relationships. For instance, the role of food security, nutrition knowledge, and women's empowerment in mediating the effect of sociodemographic variables on dietary diversity is not adequately explored.

We sincerely thank the reviewer for this valuable comment. We acknowledge that the relationships between sociodemographic factors and dietary diversity are likely to be complex and influenced by potential mediating and moderating factors. Accordingly, we have expanded the Discussion to emphasize the potential roles of household food security, nutrition knowledge, and women's empowerment in shaping dietary diversity and have recommended that future longitudinal and mediation analyses further investigate these pathways. We believe these revisions adequately address the reviewer's concerns.

Comment 55: The study's external validity is limited by its focus on a semi-urban municipality in Nepal. The findings may not be generalizable to other contexts, such as rural or urban areas, or to different cultural or socioeconomic settings. The authors should discuss the potential implications of their findings for other populations and settings, and provide recommendations for future studies to address these knowledge gaps.

We sincerely thank the reviewer for this valuable comment. We have acknowledged the limited generalizability of our findings in the Strengths and Limitations section and expanded the Discussion to describe the potential applicability of the findings to comparable semi-urban settings in Nepal and other low- and middle-income countries, while emphasizing caution when extrapolating to rural, urban, or culturally distinct populations. We have also recommended that future multisite studies across diverse geographic and socioeconomic settings be conducted to improve external validity and confirm the generalizability of our findings.

Comment 56: The sample size calculation appears to be based on an estimated prevalence of minimum dietary diversity of 45% from a similar study in a periurban area of Nepal. However, the applicability of this estimate to the current study's population is questionable, given the potential differences in socioeconomic and cultural contexts between the two settings.

We sincerely thank the reviewer for this valuable comment. The estimated prevalence of 45% was selected from the most comparable peri-urban study available at the time of study design because no prior dietary diversity estimates were available for the study area. We have clarified this rationale in the Methods section and acknowledge that differences in socioeconomic and cultural contexts may affect the applicability of this estimate. We therefore recommend that future studies use locally derived prevalence estimates where available.

Comment 57: The use of a finite population correction formula to adjust the sample size may not have fully accounted for the complexities of the study's sampling frame, which comprised 700 eligible lactating mothers identified through field enumeration. The fact that only 251 mothers completed the interviews, resulting in a response rate of 95.4%, may have introduced biases that were not adequately addressed. For instance, the exclusion of 12 mothers who declined to participate or were unavailable despite repeated follow-up visits may have led to an overrepresentation of more cooperative or accessible participants, potentially skewing the results.
Another concern lies in the assessment of dietary diversity using the MDD-W indicator, which relies on a 24-hour dietary recall method. While this approach may provide insight into the variety of foods consumed, it may not capture the nuances of dietary habits and preferences that are shaped by cultural, social, and economic factors. Additionally, the classification of foods into ten groups may oversimplify the complexity of dietary patterns, potentially masking important variations in food consumption that are relevant to maternal health outcomes.

We sincerely thank the reviewer for this thoughtful comment. We have clarified the sample size determination and sampling procedure in the Methods section and acknowledge the potential for non-response and selection bias in the Strengths and Limitations section. We also discuss the limitations of the 24-hour dietary recall and the FAO MDD-W indicator, recognizing that although MDD-W is a validated proxy for micronutrient adequacy, it does not capture the full complexity of dietary patterns or quantify food intake. We have recommended that future studies use complementary dietary assessment methods and include more comprehensive dietary measures to further validate and expand upon our findings.

Comment 58: The study's regression analysis, which aimed to identify the correlates of maternal dietary diversity, also raises questions about the selection of independent variables and the potential for multicollinearity. Although the authors reported that none of the variables exceeded the threshold for multicollinearity (VIF < 2), the inclusion of multiple sociodemographic variables, such as maternal age, educational attainment, and employment status, may have introduced redundancy and compromised the stability of the model estimates.

Note 1: The study's generalizability is limited by its focus on a specific semi-urban municipality in Nepal, which may not be representative of other contexts, either within or outside the country. The lack of consideration for potential contextual factors, such as seasonal variations in food availability, agricultural practices, and local food systems, may have further constrained the study's ability to capture the complexities of maternal dietary diversity in this setting. What is the potential impact of the finite population correction formula on the sample size, and how does it affect the generalizability of the results? How does the use of a 24-hour dietary recall method capture the nuances of dietary habits and preferences, and what are the potential biases associated with this approach? What is the justification for the selection of independent variables in the regression analysis, and how does the potential for multicollinearity affect the model estimates? How does the study's focus on a specific semi-urban municipality in Nepal limit its generalizability, and what are the implications for the development of context-specific interventions to promote maternal dietary diversity?

We sincerely thank the reviewer for this comprehensive comment. The issues raised have been addressed throughout the revised manuscript. Specifically, we have clarified the rationale for the selection of independent variables based on prior literature and theoretical relevance, confirmed that multicollinearity was assessed and found to be minimal (VIF < 2), acknowledged the limitations of the 24-hour dietary recall and the FAO MDD-W indicator, and discussed the limited generalizability of the findings due to the single semi-urban study setting. We have also highlighted the need for future longitudinal, multisite studies that consider contextual factors, such as seasonal food availability and local food systems, to better inform context-specific interventions. We believe these revisions adequately address the reviewer's concerns.

Comment 59: The demographic characteristics of the study population, as outlined in Table 2, show a skew towards younger, upper-caste, and Hindu lactating mothers. This raises questions about the representativeness of the sample, particularly given the ethnic and socioeconomic diversity of Nepal. It is essential to consider the potential biases and limitations of these demographics in the interpretation of the findings.

We sincerely thank the reviewer for this valuable comment. We acknowledge that the demographic composition of the study population reflects the characteristics of the study area and may limit the generalizability of the findings to more ethnically and socioeconomically diverse populations in Nepal. This limitation has been acknowledged in the Strengths and Limitations section, and we recommend that future studies include more diverse populations from multiple geographic settings to improve the representativeness and external validity of the findings.

Comment 60: The study's primary outcome, dietary diversity, as assessed by the MDD-W indicator, reveals that 68.1% of lactating mothers met the minimum threshold. However, the mean dietary diversity score of 5.03 (SD ± 1.25) warrants further exploration, as it suggests a narrow range of food consumption. The fact that all participants consumed starchy staples, such as rice, maize, and potatoes, is not surprising, given their cultural significance and widespread availability in Nepal.

We sincerely thank the reviewer for this valuable comment. We have expanded the **Discussion** to provide additional interpretation of the observed dietary diversity score and food group consumption patterns, emphasizing that although most mothers achieved the minimum MDD-W threshold, the limited diversity of nutrient-dense food groups indicates opportunities to further improve overall diet quality among lactating mothers.

Comment 61: The low intake of nutrient-dense food groups, such as eggs (29.1%), nuts and seeds (17.9%), and vitamin A-rich fruits and vegetables (15.5%), is concerning and may indicate micronutrient deficiencies among lactating mothers. This is particularly worrisome, as it may lead to "hidden hunger," with potential long-term consequences for both maternal and child health.

We sincerely thank the reviewer for this important comment. We have strengthened the **Discussion** by highlighting the low consumption of eggs, nuts and seeds, and vitamin A-rich fruits and vegetables, and discussing their potential implications for micronutrient deficiencies and hidden hunger among lactating mothers. We also emphasize the importance of promoting greater consumption of nutrient-dense foods through context-specific nutrition interventions and policies.

Comment 62: The study's methodology and data analysis also raise several questions. For instance, the MDD-W framework used to assess dietary diversity is based on a 24-hour recall period, which may not accurately capture usual dietary patterns. Moreover, the study does not account for potential seasonal variations in food availability and consumption, which could impact dietary diversity. How do the authors justify the generalizability of their results to the broader Nepalese population, given the demographic characteristics of the study sample? What steps were taken to validate the MDD-W framework in the context of this study, and how did the authors ensure that the 24-hour recall period accurately reflected usual dietary patterns? Can the authors provide further insight into the socioeconomic and cultural factors that contribute to the low intake of nutrient-dense food groups among lactating mothers in this population? How do the authors propose to address the potential micronutrient gaps and "hidden hunger" among lactating mothers, and what implications do these findings have for maternal and child health policies in Nepal?

We sincerely thank the reviewer for this comprehensive comment. The issues raised have been addressed throughout the revised manuscript. Specifically, we have acknowledged the limitations of the 24-hour dietary recall and the potential influence of seasonal variation in the **Strengths and Limitations** section, clarified that the FAO MDD-W is a validated indicator of dietary diversity and a proxy for micronutrient adequacy, discussed the limited generalizability of the findings beyond comparable semi-urban settings, and expanded the **Discussion** to describe the socioeconomic and cultural factors contributing to low consumption of nutrient-dense foods, the potential risk of hidden hunger, and the implications for maternal and child nutrition policies. We have also recommended future longitudinal and multisite studies using complementary dietary assessment methods to strengthen the evidence base.

Comment 63: The study's reliance on the MDD-W indicator as the primary outcome of interest is problematic. The MDD-W indicator, which assesses dietary diversity by measuring the consumption of at least five out of ten defined food groups, may not be a comprehensive measure of nutritional adequacy. The fact that 68.1% of lactating mothers met the minimum dietary diversity threshold, yet had low consumption of nutrient-dense food groups such as eggs, nuts and seeds, and vitamin A-rich fruits and vegetables, suggests that the MDD-W indicator may not capture the complexity of nutritional needs during lactation. This discrepancy raises questions about the validity of using the MDD-W indicator as a sole measure of dietary diversity in this context.

We sincerely thank the reviewer for this valuable comment. We acknowledge that the FAO Minimum Dietary Diversity for Women (MDD-W) is a proxy indicator for micronutrient adequacy and does not fully capture the quality or quantity of food consumed or the complexity of nutritional needs during lactation. We have clarified this limitation in the Discussion and Strengths and Limitations sections while emphasizing that MDD-W is a validated and internationally recommended indicator for population-level dietary assessment. We have also discussed the low consumption of nutrient-dense food groups and the potential risk of hidden hunger, and recommended that future studies incorporate complementary dietary assessment methods to provide a more comprehensive evaluation of maternal nutritional adequacy.
