# Supplementary material for: Maternal dietary diversity and its correlates in a semi-urban municipality of Nepal: A cross-sectional study
Source: PLOS Glob Public Health. 2026 Aug 25;6(8):e0005711. doi: 10.1371/journal.pgph.0005711 (PMC13505919; doi:10.1371/journal.pgph.0005711)
Supplement: Peer review history

# Peer review: Decision Letter 0

**DOI:** 10.1371/journal.pgph.0005711.r001

**Authors:** Dickson Amugsi

5 Mar 2026

PGPH-D-25-03816

Maternal dietary diversity and its correlates in a semi-urban municipality of Nepal: A cross-sectional study

PLOS Global Public Health

Dear Dr. Rimal,

Thank you for submitting your manuscript to PLOS Global Public Health. After careful consideration, we feel that it has merit but does not fully meet PLOS Global Public Health’s publication criteria as it currently stands. Therefore, we invite you to submit a revised version of the manuscript that addresses the points raised during the review process.

Please submit your revised manuscript by 16/03/2026. If you will need more time than this to complete your revisions, please reply to this message or contact the journal office at globalpubhealth@plos.org. When you're ready to submit your revision, log on to https://www.editorialmanager.com/pgph/ and select the 'Submissions Needing Revision' folder to locate your manuscript file.

Please include the following items when submitting your revised manuscript:

- A letter that responds to each point raised by the editor and reviewer(s). You should upload this letter as a separate file labeled 'Response to Reviewers'.
- A marked-up copy of your manuscript that highlights changes made to the original version. You should upload this as a separate file labeled 'Revised Manuscript with Track Changes'.
- An unmarked version of your revised paper without tracked changes. You should upload this as a separate file labeled 'Manuscript'.

Guidelines for resubmitting your figure files are available below the reviewer comments at the end of this letter.

We look forward to receiving your revised manuscript.

Kind regards,

Dickson Abanimi Amugsi, PhD

Academic Editor

PLOS Global Public Health

**Journal Requirements:**

1. Please ensure that your Ethics Statement is available in its entirety at the beginning of your Methods section, under a subheading 'Ethics Statement'.

2. We have noticed that you have uploaded Supporting Information files, but you have not included a list of legends. Please add a full list of legends for your Supporting Information files after the references list.

3. We note that your Data Availability Statement is currently as follows: All relevant data supporting the findings of this study are provided within the manuscript and its Supporting Information files.

Please confirm at this time whether or not your submission contains all raw data required to replicate the results of your study. Authors must share the “minimal data set” for their submission. PLOS defines the minimal data set to consist of the data required to replicate all study findings reported in the article, as well as related metadata and methods (https://journals.plos.org/plosone/s/data-availability#loc-minimal-data-set-definition).

For example, authors should submit the following data:

- The values behind the means, standard deviations and other measures reported;

- The values used to build graphs;

- The points extracted from images for analysis.

Authors do not need to submit their entire data set if only a portion of the data was used in the reported study.

If your submission does not contain these data, please either upload them as Supporting Information files or deposit them to a stable, public repository and provide us with the relevant URLs, DOIs, or accession numbers. For a list of recommended repositories, please see https://journals.plos.org/plosone/s/recommended-repositories.

If there are ethical or legal restrictions on sharing a de-identified data set, please explain them in detail (e.g., data contain potentially sensitive information, data are owned by a third-party organization, etc.) and who has imposed them (e.g., an ethics committee). Please also provide contact information for a data access committee, ethics committee, or other institutional body to which data requests may be sent. If data are owned by a third party, please indicate how others may request data access.

If the reviewer comments include a recommendation to cite specific previously published works, please review and evaluate these publications to determine whether they are relevant and should be cited. There is no requirement to cite these works unless the editor has indicated otherwise.

Please review your reference list to ensure that it is complete and correct. If you have cited papers that have been retracted, please include the rationale for doing so in the manuscript text, or remove these references and replace them with relevant current references. Any changes to the reference list should be mentioned in the rebuttal letter that accompanies your revised manuscript. If you need to cite a retracted article, indicate the article’s retracted status in the References list and also include a citation and full reference for the retraction notice.

**Additional Editor Comments (if provided):**

[Note: HTML markup is below. Please do not edit.]

Reviewers' comments:

Reviewer's Responses to Questions

**Comments to the Author**

1. Does this manuscript meet PLOS Global Public Health’s publication criteria?

Reviewer #1: Yes

Reviewer #2: Partly

**********

2. Has the statistical analysis been performed appropriately and rigorously?

Reviewer #1: Yes

Reviewer #2: Yes

**********

3. Have the authors made all data underlying the findings in their manuscript fully available (please refer to the Data Availability Statement at the start of the manuscript PDF file)?

The PLOS Data policy

Reviewer #1: Yes

Reviewer #2: Yes

**********

4. Is the manuscript presented in an intelligible fashion and written in standard English?

Reviewer #1: Yes

Reviewer #2: Yes

**********

Reviewer #1: The title and abstract are clear, concise, and appropriately reflect the scope and objectives of the study. The abstract provides a coherent overview of the background, methodology, key findings, and conclusions, allowing readers to quickly grasp the relevance and significance of the research. The introduction is well structured and supported by sufficient and relevant literature, effectively establishing the public health importance of maternal dietary diversity and clearly justifying the need for the study in the Nepalese semi-urban context.

In the methodology section, the authors should clearly describe how the study area was classified as a semi-urban municipality and specify the definition or criteria, along with appropriate references. Although the sources of the original questionnaire are mentioned, the authors should further report the reliability of the adapted instrument. Specifically, the Cronbach’s alpha values obtained from the pretesting should be provided to demonstrate the internal consistency and reliability of the questionnaire. Including this information would strengthen the methodological rigor of the study.

The results section is clearly presented, and the tables are well organized and easy to follow. However, the narrative accompanying Table 2 tends to repeat detailed information already provided in the table. The authors are encouraged to summarize and highlight only the key findings, rather than reiterating all tabulated values. The discussion section is satisfactory, with appropriate interpretation of the findings in relation to existing literature, and the conclusions are clear, focused, and adequately supported by the study results.

Reviewer #2: I recommend acceptance with minor revisions. The manuscript is strong and well-written, requiring only modest improvement to enhance clarity, expand the discussion of limitations and strengthen the policy relevance of the conclusions.

**********

what does this mean?). If published, this will include your full peer review and any attached files.

**Do you want your identity to be public for this peer review?**  If you choose “no”, your identity will remain anonymous but your review may still be made public.

For information about this choice, including consent withdrawal, please see our Privacy Policy

Reviewer #1: No

Reviewer #2: No

**********

[NOTE: If reviewer comments were submitted as an attachment file, they will be attached to this email and accessible via the submission site. Please log into your account, locate the manuscript record, and check for the action link "View Attachments". If this link does not appear, there are no attachment files.]

Attachment Submitted filename: Reviewer feedback.docx

# Author comment: Author response to Decision Letter 1

**DOI:** 10.1371/journal.pgph.0005711.r002

16 Mar 2026

Attachment Submitted filename: Response to reviewers.RR.docx

# Peer review: Decision Letter 1

**DOI:** 10.1371/journal.pgph.0005711.r003

**Authors:** Dickson Amugsi

25 May 2026

PGPH-D-25-03816R1

Maternal dietary diversity and its correlates in a semi-urban municipality of Nepal: A cross-sectional study

PLOS Global Public Health

Dear Dr. Rimal,

Thank you for submitting your manuscript to PLOS Global Public Health. After careful consideration, we feel that it has merit but does not fully meet PLOS Global Public Health’s publication criteria as it currently stands. Therefore, we invite you to submit a revised version of the manuscript that addresses the points raised during the review process.

Please submit your revised manuscript by 12/06/2026. If you will need more time than this to complete your revisions, please reply to this message or contact the journal office at globalpubhealth@plos.org. When you're ready to submit your revision, log on to https://www.editorialmanager.com/pgph/ and select the 'Submissions Needing Revision' folder to locate your manuscript file.

Please include the following items when submitting your revised manuscript:

- A letter that responds to each point raised by the editor and reviewer(s). You should upload this letter as a separate file labeled 'Response to Reviewers'.
- A marked-up copy of your manuscript that highlights changes made to the original version. You should upload this as a separate file labeled 'Revised Manuscript with Track Changes'.
- An unmarked version of your revised paper without tracked changes. You should upload this as a separate file labeled 'Manuscript'.

Guidelines for resubmitting your figure files are available below the reviewer comments at the end of this letter.

As the corresponding author, your ORCID iD is verified in the submission system and will appear in the published article. PLOS supports the use of ORCID, and we encourage all coauthors to register for an ORCID iD and use it as well. Please encourage your coauthors to verify their ORCID iD within the submission system before final acceptance, as unverified ORCID iDs will not appear in the published article. *Only*  the individual author can complete the verification step; PLOS staff *cannot*  verify ORCID iDs on behalf of authors.

We look forward to receiving your revised manuscript.

Kind regards,

Dickson Abanimi Amugsi, PhD

Academic Editor

PLOS Global Public Health

Journal Requirements:

If the reviewer comments include a recommendation to cite specific previously published works, please review and evaluate these publications to determine whether they are relevant and should be cited. There is no requirement to cite these works unless the editor has indicated otherwise.

Additional Editor Comments (if provided):

[Note: HTML markup is below. Please do not edit.]

Reviewers' comments:

Reviewer's Responses to Questions

**Comments to the Author**

Reviewer #1: All comments have been addressed

Reviewer #3: (No Response)

Reviewer #4: All comments have been addressed

Reviewer #5: All comments have been addressed

**********

publication criteria?

Reviewer #1: Yes

Reviewer #3: Yes

Reviewer #4: Yes

Reviewer #5: Yes

**********

3. Has the statistical analysis been performed appropriately and rigorously?

Reviewer #1: Yes

Reviewer #3: N/A

Reviewer #4: No

Reviewer #5: Yes

**********

4. Have the authors made all data underlying the findings in their manuscript fully available (please refer to the Data Availability Statement at the start of the manuscript PDF file)?

The PLOS Data policy

Reviewer #1: Yes

Reviewer #3: Yes

Reviewer #4: Yes

Reviewer #5: Yes

**********

5. Is the manuscript presented in an intelligible fashion and written in standard English?

Reviewer #1: Yes

Reviewer #3: Yes

Reviewer #4: No

Reviewer #5: Yes

**********

Reviewer #1: The authors have adequately addressed all the comments raised by the reviewers.

Reviewer #3: Manuscript ID: PGPH-D-25-03816R1

Title: Maternal dietary diversity and its correlates in a semi-urban municipality of Nepal: A cross-sectional study

General Assessment

This manuscript addresses an important and underexplored public health issue: maternal dietary diversity among lactating women in a semi-urban setting in Nepal. The topic is highly relevant to maternal and child nutrition, especially in low- and middle-income countries undergoing rapid urban transition. The study contributes useful contextual evidence regarding socioeconomic, household, and empowerment-related determinants of dietary diversity among lactating mothers.

The manuscript is generally well-structured, grounded in relevant literature, and employs recognized tools such as the MDD-W and HFIAS. The statistical analysis is mostly appropriate, and the discussion is adequately contextualized within regional and global evidence. The inclusion of empowerment, food security, and nutrition knowledge variables enhances the study's public health relevance.

However, several methodological, analytical, and reporting issues require clarification and revision before the manuscript can be considered for publication. Some inconsistencies in sample size calculations, operational definitions, interpretations of findings, and presentations of results require further attention. In addition, the manuscript would benefit from more critical interpretation of the findings, improved methodological transparency, and language refinement.

Major Comments

1. Sample Size Inconsistency and Population Correction

The manuscript reports that 700 eligible lactating mothers were identified in the municipality, but the finite population correction formula appears to use a total population size (N) of 2500 rather than 700.

This discrepancy should be clarified, as it affects the validity of the final sample size estimate.

Recommendation:

• Clearly explain the origin of N = 2500.

• Recalculate the corrected sample size if necessary.

• Ensure consistency throughout the methods section.

2. Operationalization of Women's Empowerment

Women's empowerment was categorized as "one or more decisions" versus "none."

This binary categorization oversimplifies empowerment, which is multidimensional and context-dependent.

Recommendation:

• Justify dichotomization.

• Discuss the limitations of this operational definition.

• Consider reporting the distribution of specific empowerment domains separately.

3. Cross-sectional Design and Causal Language

Several sections of the discussion and conclusion imply causal relationships between predictors and dietary diversity. For example:

• "Employment provides autonomy enabling women to make independent food choices…"

• "Nutrition education interventions improve dietary outcomes…"

Because the study is cross-sectional, causal interpretations should be avoided.

Recommendation:

• Use more cautious language, such as "associated with" rather than "improves" or "leads to."

• Reframe interpretations to reflect observational associations.

4. Dietary Assessment Limitations

The study used a single 24-hour recall, which may not adequately reflect habitual dietary intake.

Although this limitation is acknowledged later, the implications should be discussed more critically.

Recommendation:

Expand discussion on:

• day-to-day dietary variation,

• recall bias,

• seasonal variation,

• social desirability bias,

• inability to estimate nutrient adequacy quantitatively.

5. Potential Residual Confounding

The multivariable model included several predictors, but important potential confounders were not addressed, including:

• maternal BMI,

• postpartum duration,

• breastfeeding intensity,

• household food expenditure,

• cultural food taboos,

• maternal morbidity.

Recommendation:

Discuss residual confounding more explicitly in the limitations section.

6. Interpretation of "Hidden Hunger"

The manuscript states that low intake of nutrient-rich foods suggests "hidden hunger."

This is plausible, but should be interpreted cautiously because no micronutrient biomarkers or quantitative nutrient assessments were conducted.

Recommendation:

Rephrase to:

"may suggest potential micronutrient inadequacies"

Rather than implying confirmed hidden hunger.

7. ANC Visits and Statistical Interpretation

The adjusted odds ratio for ANC visits is:

aOR = 1.9 (95% CI: 1.0–3.4)

This borderline confidence interval should be interpreted cautiously.

Recommendation:

Avoid describing ANC attendance as a definitive predictor and acknowledge the marginal statistical significance.

Minor Comments

8. Clarify Time Period of Data Collection

The manuscript states:

• A study conducted from September to November 2020,

• But data collection occurred from 09/10/2020 to 24/03/2021.

These periods appear inconsistent.

Recommendation:

Clarify the exact duration of fieldwork.

9. Food Group Description

The list of food groups in the methods section appears merged:

"meat, poultry, fish, eggs, legumes, nuts, seeds…"

Recommendation:

Present the ten MDD-W groups more clearly and consistently according to the FAO classification.

10. Terminology Consistency

The manuscript alternates between:

• "dietary diversity,"

• "minimum dietary diversity,"

• "DDS,"

• "MDD-W."

Recommendation:

Use terminology consistently throughout.

11. Repetition in Discussion

Some points are repeated multiple times:

• semi-urban transition,

• education and wealth effects,

• LMIC urbanization context.

Recommendation:

Condense repetitive discussion paragraphs to improve readability.

12. Reference Formatting

Several references are incomplete or inconsistently formatted. Examples include:

• missing page ranges,

• inconsistent capitalization,

• URLs without access dates,

• formatting inconsistencies in organizational references.

Recommendation:

Ensure all references comply with PLOS formatting guidelines.

Strengths of the Study

The manuscript has several notable strengths:

1. Focus on an under-researched semi-urban population in Nepal.

2. Use of internationally recognized dietary diversity indicators (MDD-W).

3. Consideration of multiple socioeconomic and behavioral determinants.

4. High response rate (95.4%).

5. Use of stratified random sampling across all wards.

6. Inclusion of food security and empowerment dimensions.

7. Appropriate use of multivariable logistic regression with assessment of multicollinearity.

Reviewer #4: Comment 1: How do the dietary diversity and health outcomes of older lactating mothers differ from their younger counterparts, and what are the implications for public health interventions? Furthermore, the significant proportion of participants belonging to upper-caste groups and identifying as Hindu warrants an examination of the potential impact of sociocultural factors on dietary diversity and health outcomes. Specifically, what role do caste and religious affiliations play in shaping food choices and access to healthcare services in this semi-urban setting?

Comment 2: The study's findings on household characteristics, including the high percentage of male-headed households and land ownership, also merit closer examination. How do these factors influence the decision-making power and autonomy of lactating mothers in terms of their dietary choices and healthcare utilization? Additionally, the relatively even wealth distribution across quintiles may mask underlying inequalities in access to resources and healthcare services. What are the underlying mechanisms driving the observed patterns of healthcare utilization, including the low percentage of mothers who completed four or more antenatal care visits? A more nuanced analysis of the interplay between wealth, education, and empowerment is necessary to understand the complex dynamics at play. For example, how do the 43% of women categorized as empowered differ from their non-empowered counterparts in terms of their dietary diversity, healthcare utilization, and overall health outcomes?

Comment 3: What are the key factors contributing to the observed mean dietary diversity score of 5.03, and how do these factors vary across different socioeconomic and demographic subgroups? Moreover, the finding that only 23.1% of mothers completed four or more antenatal care visits highlights a critical gap in healthcare utilization. What are the underlying barriers to accessing antenatal care services, and how can these be addressed through targeted interventions? A more detailed analysis of the relationships between dietary diversity, healthcare utilization, and health outcomes is necessary to inform evidence-based policy and programmatic interventions. For instance, how do the dietary diversity scores of lactating mothers relate to their nutritional status, and what are the implications for the health and well-being of both mothers and their infants? A deeper understanding of the complex interplay between sociocultural, economic, and environmental factors is necessary to develop effective strategies for promoting maternal dietary diversity and improving health outcomes in this semi-urban municipality of Nepal.

Comment 4: The study's reliance on a cross-sectional design limits its ability to establish causal relationships between the predictor variables and maternal dietary diversity. How do the authors plan to address potential temporal relationships between these variables? Moreover, the sample size of 251 lactating mothers, although adequate for descriptive analysis, may not be sufficient to detect subtle yet significant correlations between the variables of interest. Did the authors conduct a priori power calculations to determine the required sample size?

Comment 5: The demographic characteristics of the study population, as presented in Table 2, reveal a notable skewness in the age distribution, with 69.7% of participants falling within the 18-25 year age range. How do the authors explain this uneven distribution, and what implications might it have on the representativeness of the sample? Furthermore, the dichotomization of certain variables, such as household food security and empowerment, may oversimplify the complexities of these constructs. Would the authors consider using more nuanced, continuous measures of these variables?

Comment 6: The study's use of a dietary diversity score, with a mean of 5.03 (SD ± 1.25), raises questions about the construct validity of this measure. How did the authors select the specific food groups included in the score, and what evidence supports the chosen cut-off point for minimum dietary diversity? Additionally, the lack of information about the timing and frequency of dietary assessments may introduce recall bias and limit the accuracy of the dietary data. Can the authors provide more details about their dietary assessment methodology and how they minimized potential sources of error?

Comment 7: The findings regarding the predictors of maternal dietary diversity, such as education and employment status, are intriguing but require further exploration. How do the authors propose to tease apart the relative contributions of these variables, given the high correlation between education and employment status? Moreover, the study's results indicate that only 23.1% of participants completed four or more antenatal care visits, which is concerning given the importance of ANC in promoting maternal and child health. What strategies do the authors suggest for improving ANC utilization and, by extension, maternal dietary diversity in this population?

Comment 8: “Recent global evidence suggests that targeted nutrition interventions can improve women’s dietary diversity in low- and middle-income countries” Add the following reference:

2025.Digital technology to advance global health by reviewing nutrition interventions and assessing needs in urban Syria. Discover Food 8, 416.

Comment 9: The manuscript's discussion of the study's implications for public health policy and practice is somewhat cursory. How do the authors envision their findings informing the development of targeted interventions to enhance maternal dietary diversity and improve health outcomes in this and similar populations? What specific recommendations do they propose for policymakers, healthcare providers, and other stakeholders to address the complex factors influencing maternal dietary diversity in this context?

Comment 10: The authors employed the Minimum Dietary Diversity for Women (MDD-W) indicator to assess dietary diversity among lactating mothers. However, it is essential to acknowledge that this indicator has been criticized for its limited ability to capture the nuances of dietary diversity, particularly in resource-poor settings. The MDD-W indicator focuses on the consumption of 10 predefined food groups, which may not accurately reflect the dietary habits of lactating mothers in Nepal. I would like to know whether the authors considered using alternative indicators, such as the Dietary Diversity Score (DDS) or the Food Variety Score (FVS), which may provide a more comprehensive understanding of dietary diversity.

Comment 11: The results indicate that 31.9% of participants did not achieve minimum dietary diversity, suggesting potential nutritional inadequacies. However, the authors do not provide a clear explanation for this finding. I would like to inquire about the potential factors contributing to this lack of dietary diversity, such as socioeconomic status, education level, or access to healthcare services. Additionally, it would be beneficial to know whether the authors collected data on the participants' dietary habits during pregnancy, as this may have implications for their dietary diversity during lactation. The results also show that the intake of nutrient-dense food groups, such as eggs, nuts and seeds, and vitamin A-rich fruits and vegetables, was substantially lower. This raises concerns about potential micronutrient gaps among lactating mothers. I would like to ask whether the authors considered assessing the micronutrient status of the participants, using biomarkers such as hemoglobin or serum retinol, to confirm the presence of micronutrient deficiencies.

Comment 12: Table 3 presents the proportion of lactating mothers who consumed each of the 10 food groups within the past 24 hours. However, it is unclear whether the authors accounted for the potential seasonal variability in food availability and consumption patterns. Nepal is a country with diverse geography and climate, which may impact the availability and accessibility of certain food groups. I would like to know whether the authors collected data during a specific season or whether they accounted for seasonal variations in their analysis.

Comment 13: The authors highlight the potential risk of "hidden hunger" due to the low consumption of micronutrient-rich foods. While this is an important concern, it is essential to consider the broader context of maternal nutrition in Nepal. I would like to inquire about the authors' plans to disseminate their findings to relevant stakeholders, such as policymakers, healthcare providers, and community leaders, to inform the development of targeted nutrition interventions.

Note 1: What alternative indicators were considered for assessing dietary diversity, and why was the MDD-W indicator chosen? What factors contributed to the lack of dietary diversity among 31.9% of participants, and how did these factors impact the results? Were data collected on the participants' dietary habits during pregnancy, and if so, how did these data inform the analysis? Were biomarkers used to assess the micronutrient status of participants, and if so, what were the results? How did the authors account for seasonal variability in food availability and consumption patterns? What plans do the authors have to disseminate their findings to relevant stakeholders, and how will these findings inform the development of targeted nutrition interventions?

Note 2: The study's finding that 31.9% of participants did not achieve minimum dietary diversity raises questions about the underlying factors contributing to these nutritional inadequacies. Is the low consumption of micronutrient-rich foods, such as eggs, nuts and seeds, and vitamin A-rich fruits and vegetables, a result of economic constraints, limited access to markets, or cultural preferences? Furthermore, the study's reliance on a 24-hour dietary recall may introduce recall bias, particularly among participants with limited literacy or numeracy skills. How did the researchers account for potential biases in the data collection process, and what measures were taken to ensure the accuracy and reliability of the dietary recall data?

Comment 14: Does the MDD-W indicator adequately account for the cultural significance of starchy staples, such as rice, maize, and potatoes, which are consumed by all participants, but may contribute to a high glycemic load and potentially exacerbate micronutrient deficiencies?

Comment 15: “These settings frequently undergo rapid demographic and socioeconomic changes, including migration, expansion of food markets, and shifts in dietary patterns” Add the following reference:

2025. From plate to planet: reconstructing sustainable food systems in Early Bronze Age Civilization. Human Ecology 53, 821-842.

Comment 15: How do the study's results inform our understanding of the effectiveness of existing nutrition interventions, such as the National Nutrition Policy and the Nepal Health Sector Strategy? What policy and programmatic reforms are needed to address the micronutrient gaps identified in this study, and how can these reforms be tailored to the specific cultural, economic, and environmental contexts of semi-urban municipalities in Nepal?

Comment 16: The manuscript's conclusion that nearly one-third of the population is at risk of nutritional inadequacies due to low dietary diversity warrants further investigation into the social and economic determinants of dietary diversity. What role do factors such as women's empowerment, household income, and access to education and healthcare play in shaping dietary diversity among lactating mothers? How do these factors intersect with cultural and environmental factors, such as food availability and agricultural practices, to influence dietary diversity and nutrition outcomes?

Comment 17: The authors should provide more detailed information on the sampling frame and the representativeness of the study sample. How was the sample of 251 lactating mothers selected, and what efforts were made to ensure that the sample is representative of the broader population of lactating mothers in Tarakeswor Municipality?

Comment 18: The authors should provide a clearer justification for the specific cutoff points used to define minimum dietary diversity. How did the authors determine that the chosen cutoff points accurately capture the nuances of dietary diversity among lactating mothers?

Comment 19: The multivariable logistic regression model adjusts for several sociodemographic and maternal characteristics. Nevertheless, it is unclear whether the authors controlled for other potential confounding variables, such as access to healthcare services, exposure to nutrition counseling, or the presence of comorbidities. Did the authors consider using propensity score matching or inverse probability weighting to address potential confounding?

Comment 20: The significant association between women's empowerment and dietary diversity is noteworthy. However, the authors should consider conducting a mediation analysis to elucidate the mechanisms underlying this relationship. For instance, does access to education or employment opportunities mediate the relationship between women's empowerment and dietary diversity?

Comment 21: The authors used a self-reported measure of maternal nutrition knowledge. However, this may be subject to social desirability bias or recall bias. Did the authors consider using a more objective measure of nutrition knowledge, such as a standardized questionnaire or a performance-based assessment?

Comment 22: “Understanding dietary diversity among lactating mothers in these contexts is vital for designing equitable nutrition programs” Add the following reference:

2025. Culinary chronicles of ancient Ebla: A multidisciplinary exploration of diet, nutrition, and health in a 3rd millennium BCE Syrian civilization. Journal of Anthropological Archaeology 78, 101689.

Comment 23: The association between attending four or more antenatal care visits and better dietary diversity is intriguing. However, the authors should explore the potential mechanisms underlying this relationship, such as the provision of nutrition counseling or the distribution of food supplements during ANC visits.

Comment 24: The strong association between food security and dietary diversity is not surprising. Nevertheless, the authors should consider examining the relationship between food security and dietary diversity in more detail, including the potential impact of food insecurity on dietary quality and nutrient intake.

Comment 25: The utilization of multivariable logistic regression to identify the correlates of minimum dietary diversity is commendable, but the modelling approach may have been enhanced by incorporating interaction terms to account for potential synergies between sociodemographic and maternal characteristics. For instance, the interplay between employment status and household socioeconomic position may have yield more nuanced insights into the determinants of dietary diversity.

Note 3: The study's reliance on self-reported data for variables such as food security and maternal nutrition knowledge may introduce biases, as respondents' perceptions of these constructs may not align with objective measures. Therefore, it is essential to consider the development of more robust instruments for assessing these variables, such as the use of standardized questionnaires or observational methods. Moreover, the finding that women's empowerment is a significant predictor of dietary diversity raises questions about the underlying mechanisms driving this relationship. Is it the case that empowered women are more likely to prioritize their own nutritional needs, or do they possess greater autonomy to make decisions about food choices within their households?

Note 4: The generalizability of the findings to other contexts, particularly in urban or rural areas with distinct sociocultural and economic profiles, is uncertain. Consequently, it is crucial to replicate this study in diverse settings to substantiate the results and identify potential variations in the factors associated with dietary diversity. Ultimately, a more comprehensive understanding of the complex interplay between maternal characteristics, household factors, and dietary diversity is essential for devising effective strategies to promote optimal nutrition among lactating mothers in Nepal and beyond.

Note 5: How can healthcare providers and policymakers leverage the association between women's empowerment and dietary diversity to inform the development of interventions aimed at promoting maternal nutrition? What role can community-based initiatives, such as nutrition education programs or support groups, play in enhancing dietary diversity among lactating mothers? By exploring these questions, the study could have provided a more concrete foundation for translating its findings into actionable recommendations for improving maternal health outcomes.

Comment 26: The mean DDS was reported as 5.03 (±1.25), with 68% of mothers meeting the MDD-W threshold. However, the process of calculating the DDS and the specific food groups considered under the MDD-W framework are not adequately described. Could the authors provide a detailed explanation of their methodology, including how they handled potential variations in food group classifications and portion sizes?

Comment 27: The study found that maternal education, employment status, household wealth, and food security were significant predictors of dietary diversity. Yet, the mechanisms through which these factors influence dietary choices are complex and multifaceted. How do the authors suggest policymakers and practitioners address these socioeconomic determinants to improve maternal dietary diversity, especially in semi-urban settings where access to resources can be limited and uneven?

Comment 28: What specific nutrition education strategies do the authors recommend integrating into antenatal and postnatal care services to enhance maternal dietary diversity, and how can these strategies be tailored to the semi-urban context of Nepal?

Comment 29: How do the authors account for the potential role of cultural beliefs and practices in shaping maternal dietary diversity in semi-urban Nepal, and what implications does this have for the design of culturally sensitive nutrition interventions?

Comment 30: The study highlights the need for multi-sectoral strategies that address both structural and behavioral barriers to improving maternal dietary diversity. What specific policy recommendations do the authors have for the government of Nepal and other low- and middle-income countries facing similar challenges, particularly in terms of integrating nutrition-sensitive interventions with social protection programs and livelihood support for women?

Comment 31: “Similarly, employment status was positively associated with dietary diversity” Add the following reference:

2024. A cross-sectional study on demographic characteristics, nutritional knowledge, and supplement use patterns. International Journal of Kinesiology & Sports Science 12 (4), 21-30.

Comment 32: How do the intersecting dynamics of urbanization, poverty, and gender norms influence maternal dietary practices, and what are the implications for the design of effective nutrition interventions?

Comment 33: What are the most effective strategies for delivering nutrition education and counseling to lactating mothers, and how can these interventions be integrated into existing healthcare systems and community-based programs? Additionally, how can policymakers and practitioners balance the need for culturally sensitive and context-specific interventions with the need for scalable and sustainable solutions that can be replicated across diverse settings?

Reviewer #5: Introduction

It looks good and reflects your work properly.

Abstract:

The abstract represents adjusted odds ratios from Table 4, as it appears internally inconsistent. Check Table 4 and rerun calculations and then write abstract again

Introduction:

The operational definition of semi-urban should be based on Nepalese administrative classification or a recognized urbanization framework, not a general website. The paragraph on semi-urban food environments is useful but should be linked more directly to the study municipality and available local data.

A study area map can be more useful to visualize the study area, and the research gap should be more clarified.

Sample size:

In line 145, even though they have stated that 700 eligible lactating mothers were identified across 11 wards, the finite population correction formula uses N = 2500. This is not explained. There are also calculation errors related to adjusted n. The authors must clarify the true sampling frame, redo the calculation, and revise the sampling section accordingly.

Contradiction between selected sample and completed sample:

In line 128, it is stated that 251 mothers were selected using proportionate stratified random sampling, but Table 1 says 263 were selected and then 251 completed interviews (Line 159). This should be corrected too; it may mislead the calculations.

Study dates:

In the study and design setting, Line (115-117) study period and data collection period are not matched and use a specific date format.

MDD-W food groups are incorrectly described in the Methods

Although table 3 uses the correct-looking groups, in line 169, you have written "oils and fats." So, check the MDD-W 10 standard food groups of the FAO.

Independent variables

The research can add more independent variables such as premarital conditions, number of children, age of giving birth, number of family members, etc. Education, age, wealth, food security, and empowerment categories should be described exactly as used in regression.

Table 4 appears mathematically impossible:

The final sample size is n = 251, and Table 2 reports dietary diversity as 171 diverse and 80 not diverse. However, many Table 4 rows have totals that exceed n = 251 or contradict Table 2. For example, the age-group rows appear to total 251 for <=24 years and 84 for >24 years, giving 335 observations. Family type, education, employment, food security, nutrition knowledge, and other variables show similar problems. This undermines all reported odds ratios and confidence intervals.

Variable coding:

There is inconsistent reporting of variable coding. The Methods, Table 2, and Table 4 classify age, wealth, education, and other factors in different ways. Before providing regression findings, the authors should clearly explain all recoding and collapsing choices.

The number of predictors compared to the number of result events may cause the regression model to be overfitted. You should describe the precise variables included in the model, provide justification for the model definition, and take into account a more economical model.

Regression model:

The number of predictors compared to the number of result events may cause the regression model to be overfitted. The authors should describe the precise variables included in the model, provide justification for the model definition, and take into account a more economical model.

Inconsistent interpretation of statistical significance

The ANC result should be reported with aOR, CI, and additionally an exact P value to make interpretation significant.

Conclusion:

Even though education and nutrition knowledge have larger reported adjusted odds ratios than food security, you stated food insecurity was the strongest barrier. So, careful justification is needed.

References:

Careful checking of the reference list is necessary. A number of references, such as the citation for Bennett's Law and URLs with tracking artifacts, are weak, inconsistent, or incomplete.

**********

what does this mean?). If published, this will include your full peer review and any attached files.

**Do you want your identity to be public for this peer review?**  If you choose “no”, your identity will remain anonymous but your review may still be made public.

For information about this choice, including consent withdrawal, please see our Privacy Policy

Reviewer #1: No

Reviewer #3: **Yes:**  Abdulganiyyu A. Ibrahim

Reviewer #4: **Yes:**  Loai Aljerf

Reviewer #5: **Yes:**  Md.Yousuf Ali

**********

[NOTE: If reviewer comments were submitted as an attachment file, they will be attached to this email and accessible via the submission site. Please log into your account, locate the manuscript record, and check for the action link "View Attachments". If this link does not appear, there are no attachment files.]

# Author comment: Author response to Decision Letter 2

**DOI:** 10.1371/journal.pgph.0005711.r004

30 Jun 2026

Attachment Submitted filename: Response to reviewers .docx

# Peer review: Decision Letter 2

**DOI:** 10.1371/journal.pgph.0005711.r005

**Authors:** Dickson Amugsi

21 Jul 2026

PGPH-D-25-03816R2

Maternal dietary diversity and its correlates in a semi-urban municipality of Nepal: A cross-sectional study

PLOS Global Public Health

Dear Dr. Rimal,

Thank you for submitting your manuscript to PLOS Global Public Health. After careful consideration, we feel that it has merit but does not fully meet PLOS Global Public Health’s publication criteria as it currently stands. Therefore, we invite you to submit a revised version of the manuscript that addresses the points raised during the review process.

Please ensure that your decision is justified on PLOS Global Public Health’s publication criteria and not, for example, on novelty or perceived impact.

Please submit your revised manuscript by 5/08/2026. If you will need more time than this to complete your revisions, please reply to this message or contact the journal office at globalpubhealth@plos.org. When you're ready to submit your revision, log on to https://www.editorialmanager.com/pgph/ and select the 'Submissions Needing Revision' folder to locate your manuscript file.

Please include the following items when submitting your revised manuscript:

- A letter that responds to each point raised by the editor and reviewer(s). You should upload this letter as a separate file labeled 'Response to Reviewers'.
- A marked-up copy of your manuscript that highlights changes made to the original version. You should upload this as a separate file labeled 'Revised Manuscript with Track Changes'.
- An unmarked version of your revised paper without tracked changes. You should upload this as a separate file labeled 'Manuscript'.

Guidelines for resubmitting your figure files are available below the reviewer comments at the end of this letter.

As the corresponding author, your ORCID iD is verified in the submission system and will appear in the published article. PLOS supports the use of ORCID, and we encourage all coauthors to register for an ORCID iD and use it as well. Please encourage your coauthors to verify their ORCID iD within the submission system before final acceptance, as unverified ORCID iDs will not appear in the published article. *Only*  the individual author can complete the verification step; PLOS staff *cannot*  verify ORCID iDs on behalf of authors.

We look forward to receiving your revised manuscript.

Kind regards,

Dickson Abanimi Amugsi, PhD

Academic Editor

PLOS Global Public Health

Journal Requirements:

If the reviewer comments include a recommendation to cite specific previously published works, please review and evaluate these publications to determine whether they are relevant and should be cited. There is no requirement to cite these works unless the editor has indicated otherwise.

Please review your reference list to ensure that it is complete and correct. If you have cited papers that have been retracted, please include the rationale for doing so in the manuscript text, or remove these references and replace them with relevant current references. Any changes to the reference list should be mentioned in the rebuttal letter that accompanies your revised manuscript. If you need to cite a retracted article, indicate the article’s retracted status in the References list and also include a citation and full reference for the retraction notice.

Additional Editor Comments (if provided):

Thank you for submitting your manuscript for publication. It has gone through several rounds of review. Before I make a final decision, I would like to invite you to carefully address the feedback provided by Reviewer 4 and then resubmit your manuscript for my final evaluation.

NB: Two reviewers have already recommended the manuscript for publication. Therefore, my final decision will depend on how well you address the feedback mentioned above.

[Note: HTML markup is below. Please do not edit.]

Reviewers' comments:

Reviewer's Responses to Questions

**Comments to the Author**

Reviewer #4: (No Response)

Reviewer #6: All comments have been addressed

**********

publication criteria?

Reviewer #4: No

Reviewer #6: Yes

**********

3. Has the statistical analysis been performed appropriately and rigorously?

Reviewer #4: No

Reviewer #6: Yes

**********

4. Have the authors made all data underlying the findings in their manuscript fully available (please refer to the Data Availability Statement at the start of the manuscript PDF file)?

The PLOS Data policy

Reviewer #4: No

Reviewer #6: Yes

**********

5. Is the manuscript presented in an intelligible fashion and written in standard English?

Reviewer #4: No

Reviewer #6: Yes

**********

**Review Comments to the Author**

Reviewer #4: I highly recommend “Completely Reject” due to the following reasons:

1- Poor response.

2- Chopping response.

3- Erratic report.

4- Incomplete study.

5- Unauthenticated results.

I reiterate my report after blasting All the authors’ responses and add new comments:

Comment 34: The manuscript provides a critical foundation for understanding the complex relationships between dietary diversity, nutrition, and health among lactating mothers in Nepal. However, to fully capture the nuances of these relationships and to inform effective policy and programmatic responses, further research is needed to address the methodological, theoretical, and contextual limitations of the study. By doing so, researchers and policymakers can work together to develop more effective and sustainable solutions to address the pressing issue of "hidden hunger" and promote optimal nutrition and health outcomes among lactating mothers and their children.

Comment 35: The study's findings may not be generalizable to other contexts, particularly in urban or rural areas with distinct sociocultural and economic profiles. The authors should discuss the potential limitations of their study in terms of external validity and the need for further research to replicate their findings in diverse settings.

Comment 36: The study employed a cross-sectional design, which, while useful for establishing associations, does not allow for the determination of causality. How do the authors plan to address the potential limitations of their study design in terms of implying causal relationships between the observed correlates and maternal dietary diversity?

Comment 37: Given the cross-sectional nature of the study, longitudinal research designs could provide valuable insights into how maternal dietary diversity changes over time and in response to interventions. What directions do the authors propose for investigating the dynamics of maternal dietary diversity in semi-urban settings, and how can such research contribute to the development of effective, sustainable nutrition programs?

Comment 38: The study's findings also highlight the need for more research on the relationships between dietary diversity, micronutrient intake, and maternal and child health outcomes in semi-urban settings. What are the potential consequences of "hidden hunger" and micronutrient deficiencies for maternal and child health, and how can nutrition interventions be designed to address these needs? Moreover, how can the study's findings be used to inform the development of national nutrition policies and programs, such as Nepal's Multi-Sector Nutrition Plan, and what are the implications for the design of targeted interventions in comparable settings undergoing rapid urban transitions?

Comment 39: The study relies on a cross-sectional design, which may not be suitable for establishing causal relationships between dietary diversity and its determinants. The authors should justify the choice of study design and discuss the potential limitations of using a cross-sectional approach.

Comment 40: The study uses the Minimum Dietary Diversity for Women (MDD-W) indicator, which is defined as the consumption of at least five out of ten specified food groups in the previous 24 hours. However, the authors do not provide sufficient information on how the food groups were defined and measured. For instance, how were the food groups classified, and what specific foods were included in each group?

Comment 41: The study also reports that more than half of Nepali women of reproductive age do not meet the MDD-W threshold, highlighting widespread dietary inadequacy and vulnerability to micronutrient deficiencies. However, the authors do not provide sufficient data to support this claim. What are the specific micronutrient deficiencies that are prevalent among lactating mothers in Nepal, and how do these deficiencies impact maternal and child health?

Comment 42: The study highlights the importance of combining nutrition education with broader social and economic support to improve maternal dietary practices. However, the authors do not provide sufficient evidence to support this claim. What specific nutrition education programs and social and economic support interventions have been shown to be effective in improving dietary diversity among lactating mothers in semi-urban settings?

Comment 43: The study notes that household food insecurity is a primary barrier to dietary diversity, but the authors do not provide sufficient information on how food insecurity was measured. What specific indicators were used to assess food insecurity, and how were these indicators validated?

Comment 44: The study also highlights the importance of understanding dietary diversity among lactating mothers in semi-urban settings, but the authors do not provide sufficient context on the specific semi-urban municipality of Nepal that was studied. What are the unique characteristics of this municipality, and how do these characteristics impact dietary diversity among lactating mothers?

Comment 45: The study aims to inform locally tailored, equity-driven interventions to promote maternal dietary diversity and improve health outcomes in rapidly urbanizing LMIC settings. However, the authors do not provide sufficient information on how the study's findings can be translated into practice. What specific policy and programmatic recommendations can be made based on the study's findings, and how can these recommendations be implemented in a way that is culturally and contextually relevant?

Comment 46: What would be the justification of using the MDD-W indicator in this context, and how the results of this study can be compared with the other studies which have used different indicators? How the authors can rule out the possibility that the observed associations between dietary diversity and its determinants are due to residual confounding or reverse causality? What would be the potential generalizability of the study findings to other semi-urban settings in Nepal and other LMICs, and how the authors can justify the generalizability of the results? How the study's findings can be used to inform policy and programmatic decisions to improve maternal dietary diversity in semi-urban settings, and what specific recommendations can be made based on the study's results?

Comment 47: The authors should provide more context on the specific semi-urban municipality of Nepal that was studied, and discuss the potential generalizability of the study findings to other settings. The authors should also provide more information on the potential implications of the study's findings for policy and programmatic decisions, and discuss the potential avenues on maternal dietary diversity in semi-urban settings. So, you must perform (1) Longitudinal studies to examine the causal relationships between dietary diversity and its determinants, (2) Intervention studies to evaluate the effectiveness of specific nutrition education programs and social and economic support interventions in improving dietary diversity among lactating mothers, (3) Qualitative studies to explore the cultural and contextual factors that influence dietary diversity among lactating mothers in semi-urban settings, and (4) Studies to examine the potential generalizability of the study findings to other semi-urban settings in Nepal and other LMICs.

Comment 48: The study's reliance on a cross-sectional design limitations its ability to establish causality between the variables under investigation. The authors' conclusions about the relationships between dietary diversity and various sociodemographic, economic, and household-level factors are thus based on correlations rather than causal links. For instance, the authors suggest that household food insecurity is a primary barrier to dietary diversity, but they do not provide longitudinal data to demonstrate how changes in food security status affect dietary diversity over time. Furthermore, the study's use of the MDD-W indicator as the primary outcome measure may not capture the complexity of dietary patterns among lactating mothers. The MDD-W indicator is based on the consumption of at least five out of ten specified food groups in the previous 24 hours, but it does not account for the quality or quantity of food consumed within each group. This oversimplification may lead to misleading conclusions about the adequacy of maternal diets. For example, a mother who consumes a diet rich in starchy staples but limited in animal-source foods, legumes, fruits, and vegetables may meet the MDD-W threshold but still be at risk of micronutrient deficiencies. The authors also propose that targeted nutrition interventions, such as behavior change communication, nutrition education, and women's empowerment programs, can improve dietary diversity among lactating mothers. However, the evidence for these interventions is largely based on studies conducted in rural or urban areas, and it is unclear whether these interventions would be effective in semi-urban settings. The unique socioeconomic and environmental characteristics of semi-urban areas, including mixed livelihoods, evolving food systems, and varying access to services, may require tailored interventions that address the specific needs and constraints of these populations.

Comment 49: The study's sample size and selection criteria are not clearly justified. The authors do not provide information on how the sample size was determined or how participants were selected, which raises concerns about the representativeness of the sample and the potential for selection bias. Furthermore, the study's focus on lactating mothers in a single semi-urban municipality limits the generalizability of the findings to other populations and settings.

Comment 50: The authors' conclusion that the study's findings can inform locally tailored, equity-driven interventions to promote maternal dietary diversity and improve health outcomes in rapidly urbanizing LMIC settings is overly optimistic. While the study provides some insights into the dietary diversity and correlates among lactating mothers in a semi-urban municipality of Nepal, the methodological limitations and lack of generalizability of the findings undermine the study's potential to inform policy and practice. Further research is needed to address the complex and context-specific challenges of promoting dietary diversity and improving maternal and child health in semi-urban settings.

Comment 51: The study's sample size calculation appears to be based on an estimated prevalence of minimum dietary diversity (45%) from a similar study done in a periurban area of Nepal. However, the authors do not provide sufficient justification for using this specific prevalence estimate, which may not accurately represent the study population. This raises questions about the generalizability of the findings. Furthermore, the study uses a 24-hour dietary recall method to assess dietary diversity, which may be subject to recall bias. While the authors attempt to minimize this bias by using probing questions and locally familiar food examples, the reliability of this method is still uncertain. It would be beneficial to explore alternative methods, such as food diaries or weighed food records, to validate the findings.

Comment 52: The study also categorizes women's empowerment as a binary variable, which may oversimplify the complex and multifaceted nature of empowerment. The decision to dichotomize this variable may lead to loss of information and potential misclassification of participants. A more nuanced approach, such as using a continuous or ordinal scale, may provide a more accurate representation of women's empowerment.

Comment 53: The study controls for various sociodemographic variables, including wealth quintiles, which are collapsed into three categories. However, the authors do not provide a clear justification for this categorization, which may mask important differences between the wealthiest and poorest quintiles. It is essential to explore alternative categorizations or use continuous variables to capture the full range of wealth disparities.

Comment 54: The study's regression analysis reveals several significant associations between independent variables and dietary diversity. However, the authors do not provide sufficient discussion on the potential mediating or moderating factors that may influence these relationships. For instance, the role of food security, nutrition knowledge, and women's empowerment in mediating the effect of sociodemographic variables on dietary diversity is not adequately explored.

Comment 55: The study's external validity is limited by its focus on a semi-urban municipality in Nepal. The findings may not be generalizable to other contexts, such as rural or urban areas, or to different cultural or socioeconomic settings. The authors should discuss the potential implications of their findings for other populations and settings, and provide recommendations for future studies to address these knowledge gaps.

Comment 56: The sample size calculation appears to be based on an estimated prevalence of minimum dietary diversity of 45% from a similar study in a periurban area of Nepal. However, the applicability of this estimate to the current study's population is questionable, given the potential differences in socioeconomic and cultural contexts between the two settings.

Comment 57: The use of a finite population correction formula to adjust the sample size may not have fully accounted for the complexities of the study's sampling frame, which comprised 700 eligible lactating mothers identified through field enumeration. The fact that only 251 mothers completed the interviews, resulting in a response rate of 95.4%, may have introduced biases that were not adequately addressed. For instance, the exclusion of 12 mothers who declined to participate or were unavailable despite repeated follow-up visits may have led to an overrepresentation of more cooperative or accessible participants, potentially skewing the results.

Another concern lies in the assessment of dietary diversity using the MDD-W indicator, which relies on a 24-hour dietary recall method. While this approach may provide insight into the variety of foods consumed, it may not capture the nuances of dietary habits and preferences that are shaped by cultural, social, and economic factors. Additionally, the classification of foods into ten groups may oversimplify the complexity of dietary patterns, potentially masking important variations in food consumption that are relevant to maternal health outcomes.

Comment 58: The study's regression analysis, which aimed to identify the correlates of maternal dietary diversity, also raises questions about the selection of independent variables and the potential for multicollinearity. Although the authors reported that none of the variables exceeded the threshold for multicollinearity (VIF < 2), the inclusion of multiple sociodemographic variables, such as maternal age, educational attainment, and employment status, may have introduced redundancy and compromised the stability of the model estimates.

Note 1: The study's generalizability is limited by its focus on a specific semi-urban municipality in Nepal, which may not be representative of other contexts, either within or outside the country. The lack of consideration for potential contextual factors, such as seasonal variations in food availability, agricultural practices, and local food systems, may have further constrained the study's ability to capture the complexities of maternal dietary diversity in this setting. What is the potential impact of the finite population correction formula on the sample size, and how does it affect the generalizability of the results? How does the use of a 24-hour dietary recall method capture the nuances of dietary habits and preferences, and what are the potential biases associated with this approach? What is the justification for the selection of independent variables in the regression analysis, and how does the potential for multicollinearity affect the model estimates? How does the study's focus on a specific semi-urban municipality in Nepal limit its generalizability, and what are the implications for the development of context-specific interventions to promote maternal dietary diversity?

Comment 59: The demographic characteristics of the study population, as outlined in Table 2, show a skew towards younger, upper-caste, and Hindu lactating mothers. This raises questions about the representativeness of the sample, particularly given the ethnic and socioeconomic diversity of Nepal. It is essential to consider the potential biases and limitations of these demographics in the interpretation of the findings.

Comment 60: The study's primary outcome, dietary diversity, as assessed by the MDD-W indicator, reveals that 68.1% of lactating mothers met the minimum threshold. However, the mean dietary diversity score of 5.03 (SD ± 1.25) warrants further exploration, as it suggests a narrow range of food consumption. The fact that all participants consumed starchy staples, such as rice, maize, and potatoes, is not surprising, given their cultural significance and widespread availability in Nepal.

Comment 61: The low intake of nutrient-dense food groups, such as eggs (29.1%), nuts and seeds (17.9%), and vitamin A-rich fruits and vegetables (15.5%), is concerning and may indicate micronutrient deficiencies among lactating mothers. This is particularly worrisome, as it may lead to "hidden hunger," with potential long-term consequences for both maternal and child health.

Comment 62: The study's methodology and data analysis also raise several questions. For instance, the MDD-W framework used to assess dietary diversity is based on a 24-hour recall period, which may not accurately capture usual dietary patterns. Moreover, the study does not account for potential seasonal variations in food availability and consumption, which could impact dietary diversity. How do the authors justify the generalizability of their results to the broader Nepalese population, given the demographic characteristics of the study sample? What steps were taken to validate the MDD-W framework in the context of this study, and how did the authors ensure that the 24-hour recall period accurately reflected usual dietary patterns? Can the authors provide further insight into the socioeconomic and cultural factors that contribute to the low intake of nutrient-dense food groups among lactating mothers in this population? How do the authors propose to address the potential micronutrient gaps and "hidden hunger" among lactating mothers, and what implications do these findings have for maternal and child health policies in Nepal?

Comment 63: The study's reliance on the MDD-W indicator as the primary outcome of interest is problematic. The MDD-W indicator, which assesses dietary diversity by measuring the consumption of at least five out of ten defined food groups, may not be a comprehensive measure of nutritional adequacy. The fact that 68.1% of lactating mothers met the minimum dietary diversity threshold, yet had low consumption of nutrient-dense food groups such as eggs, nuts and seeds, and vitamin A-rich fruits and vegetables, suggests that the MDD-W indicator may not capture the complexity of nutritional needs during lactation. This discrepancy raises questions about the validity of using the MDD-W indicator as a sole measure of dietary diversity in this context.

Reviewer #6: Reviewer Recommendation

I carefully reviewed the revised manuscript and the authors' responses to the previous review comments. I am pleased to note that all of my comments and suggestions have been satisfactorily addressed in this revised version. The authors have made substantial improvements to the manuscript, enhancing its scientific rigor, clarity, methodological transparency, and overall quality.

The manuscript now presents a well-structured and valuable contribution to the field of maternal nutrition and public health. The study addresses an important public health issue, employs appropriate methodology, and the findings have clear implications for policy and practice.

Based on the satisfactory revisions, I have no further comments and recommend that the manuscript be accepted for publication in its current form.

**********

what does this mean?). If published, this will include your full peer review and any attached files.

**Do you want your identity to be public for this peer review?**  If you choose “no”, your identity will remain anonymous but your review may still be made public.

For information about this choice, including consent withdrawal, please see our Privacy Policy

Reviewer #4: No

Reviewer #6: No

**********

[NOTE: If reviewer comments were submitted as an attachment file, they will be attached to this email and accessible via the submission site. Please log into your account, locate the manuscript record, and check for the action link "View Attachments". If this link does not appear, there are no attachment files.]

# Author comment: Author response to Decision Letter 3

**DOI:** 10.1371/journal.pgph.0005711.r006

23 Jul 2026

Attachment Submitted filename: Response to reviewers.docx

# Editor report: Decision Letter 3

**DOI:** 10.1371/journal.pgph.0005711.r007

**Authors:** Dickson Amugsi

26 Jul 2026

Maternal dietary diversity and its correlates in a semi-urban municipality of Nepal: A cross-sectional study

PGPH-D-25-03816R3

Dear Rimal,

We are pleased to inform you that your manuscript 'Maternal dietary diversity and its correlates in a semi-urban municipality of Nepal: A cross-sectional study' has been provisionally accepted for publication in PLOS Global Public Health.

Before your manuscript can be formally accepted you will need to complete some formatting changes, which you will receive in a follow up email. A member of our team will be in touch with a set of requests.

Please note that your manuscript will not be scheduled for publication until you have made the required changes, so a swift response is appreciated.

IMPORTANT: The editorial review process is now complete. PLOS will only permit corrections to spelling, formatting or significant scientific errors from this point onwards. Requests for major changes, or any which affect the scientific understanding of your work, will cause delays to the publication date of your manuscript.

If your institution or institutions have a press office, please notify them about your upcoming paper to help maximize its impact. If they'll be preparing press materials, please inform our press team as soon as possible -- no later than 48 hours after receiving the formal acceptance. Your manuscript will remain under strict press embargo until 2 pm Eastern Time on the date of publication. For more information, please contact globalpubhealth@plos.org.

Thank you again for supporting Open Access publishing; we are looking forward to publishing your work in PLOS Global Public Health.

Best regards,

Dickson Abanimi Amugsi, PhD

Academic Editor

PLOS Global Public Health

***********************************************************

Reviewer Comments (if any, and for reference):
